# Stereoselective, nitro-Mannich/lactamisation cascades for the direct synthesis of heavily decorated 5-nitropiperidin-2-ones and related heterocycles

**DOI:** 10.3762/bjoc.8.64

**Published:** 2012-04-16

**Authors:** Pavol Jakubec, Dane M Cockfield, Madeleine Helliwell, James Raftery, Darren J Dixon

**Affiliations:** 1 The Department of Chemistry, Chemistry Research Laboratory, University of Oxford, Mansfield Road, Oxford OX1 3TA, UK; 2 School of Chemistry, The University of Manchester, Oxford Road, Manchester, M13 9PL, UK

**Keywords:** cascade, imine, Michael addition, nitro-Mannich, organocatalysis, piperidine alkaloids

## Abstract

A versatile nitro-Mannich/lactamisation cascade for the direct stereoselective synthesis of heavily decorated 5-nitropiperidin-2-ones and related heterocycles has been developed. A highly enantioenriched substituted 5-nitropiperidin-2-one was synthesised in a four component one-pot reaction combining an enantioselective organocatalytic Michael addition with the diastereoselective nitro-Mannich/lactamisation cascade. Protodenitration and chemoselective reductive manipulation of the heterocycles was used to install contiguous and fully substituted stereocentres in the synthesis of substituted piperidines.

## Introduction

**Figure 1:**
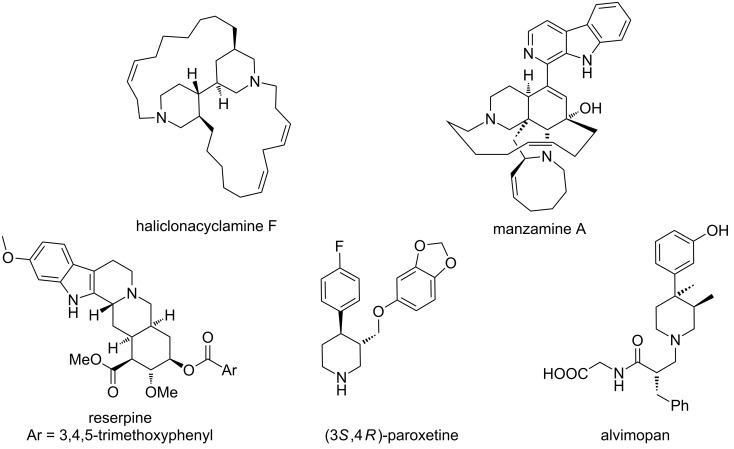
Biologically active natural products and drugs containing the piperidine ring.

The piperidine ring is a common motif found in many biologically active natural products and drugs. The structures of these compounds range from the architecturally complex polycyclic ring systems, such as those found in the alkaloids haliclonacyclamine F [1], manzamine A [2–6], and reserpine [7–8] (Figure 1), to relatively simple piperidines found in pharmaceutical compounds, such as paroxetine [9–10] and alvimopan [11].

The abundance of this motif in desirable targets has led to considerable interest from the synthetic community [12–19]. Common synthetic approaches to incorporate this motif include nucleophilic additions to pyridine rings and further manipulation [20–25], intramolecular iminium ion cyclisation [26–29], reduction of unsaturated heterocycles [30–32], ring closure via intramolecular nucleophilic substitution [33–37], cascade reactions of enamines/imines and aldehydes [38–41], and ring-closing metathesis followed by hydrogenation [42–48]. Arguably the most general route employs cycloadditions and subsequent manipulation of the partially unsaturated ring system [49–53]. We believed a powerful entry to piperidine rings and related heterocyclic structures could employ a nitro-Mannich/lactamisation cascade of γ-nitro ester starting materials with imines (cyclic or acyclic, preformed or formed in situ) as a key step. Not only could this approach allow the rapid generation of structural complexity, but the products would be amenable to further synthetic transformations. Furthermore, the γ-nitro ester starting materials are accessible in an enantioenriched form by using an organocatalytic Michael addition methodology, which was developed by our group and others [54–59]. In pursuit of this we have successfully harnessed the power of the nitro-Mannich/lactamisation cascade in a formal synthesis of (3*S*,4*R*)-paroxetine [60], in the construction of architecturally complex polycyclic alkaloid structures [61] and more recently as a key complexity building step in the total synthesis of nakadomarin A [62–65]. Herein we wish to report our full findings in this synthetically powerful cyclisation cascade.

**Scheme 1:**
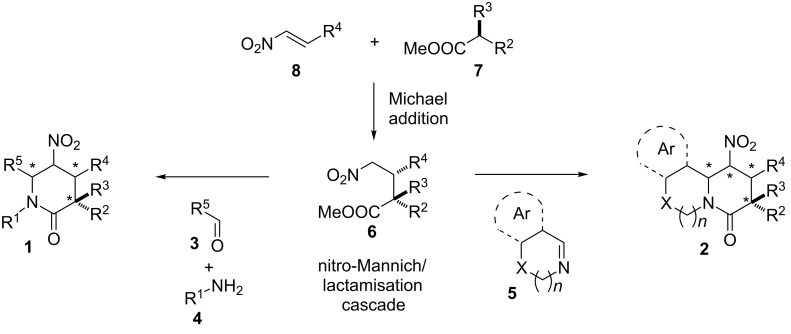
A general strategy to 5-nitropiperidin-2-ones and related heterocycles.

The first example of a simple nitro-Mannich/lactamisation cascade was reported independently by Mühlstädt and Jain in the mid-1970s [66–67]. The condensation of methyl 4-nitrobutanoate **6** (Scheme 1; R^2^ = R^3^ = R^4^ = H) with aromatic aldehydes **3** (R^5^ = Ar) and ammonium acetate provided access to simple 6-aryl-substituted 5-nitropiperidin-2-ones **1** (R^1^ = R^2^ = R^3^ = R^4^ = H, R^5^ = Ar). The power of this transformation was not immediately recognised and only in the last two decades has the cascade been successfully applied to the synthesis of simple biologically active compounds and their precursors, such as (±)-CP-99,994 [68–69], inhibitors of farnesyltransferase [70–71], selective dipeptidyl peptidase IV inhibitors [72–73], and functionalised bispidines [74]. Very recently, a related cascade inspired by the original work of Jain incorporating C–C bond formation was accomplished through a nitro-Mannich reaction [75–80] of nitro carbonyl compounds with imines, followed by ring-closure condensation [81–83]. Despite improvements of, and developments to, the nitro-Mannich/lactamisation cascade during the last few decades, we recognised, that further enhancement of the method was necessary to transform it into a general synthetic tool of use in both medicinal chemistry and natural-product synthesis.

## Results and Discussion

**Scheme 2:**
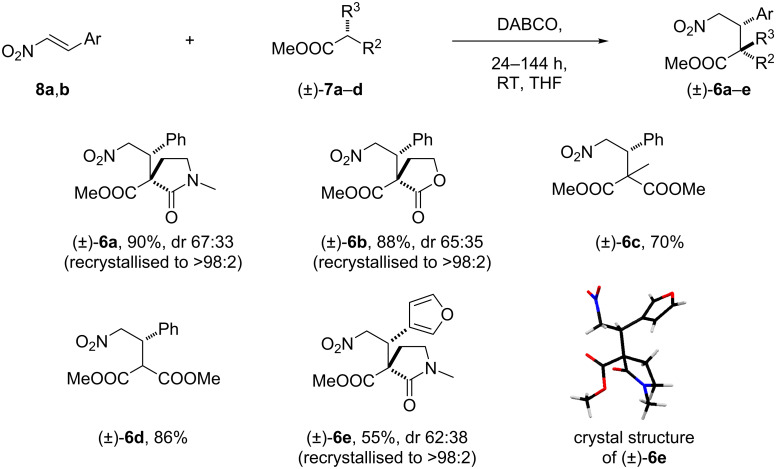
The synthesis of Michael adduct model substrates for the nitro-Mannich/lactamisation cascade.

To allow us to further explore the nitro-Mannich/lactamisation cascade, a range of Michael adducts **6a–e** were synthesised on a gram scale by the reaction of active methylene or methine carbon acids with nitro olefins in the presence of DABCO (20–30 mol %) in THF (Scheme 2). Where diastereoisomers were created in the Michael addition step and stereocontrol was poor, the diastereomeric mixtures were recrystallised to afford single diastereomers **6a**, **b**, **e**. The relative stereochemistry of the major diastereomer **6e** was assigned unambiguously by single-crystal X-ray analysis.

**Scheme 3:**
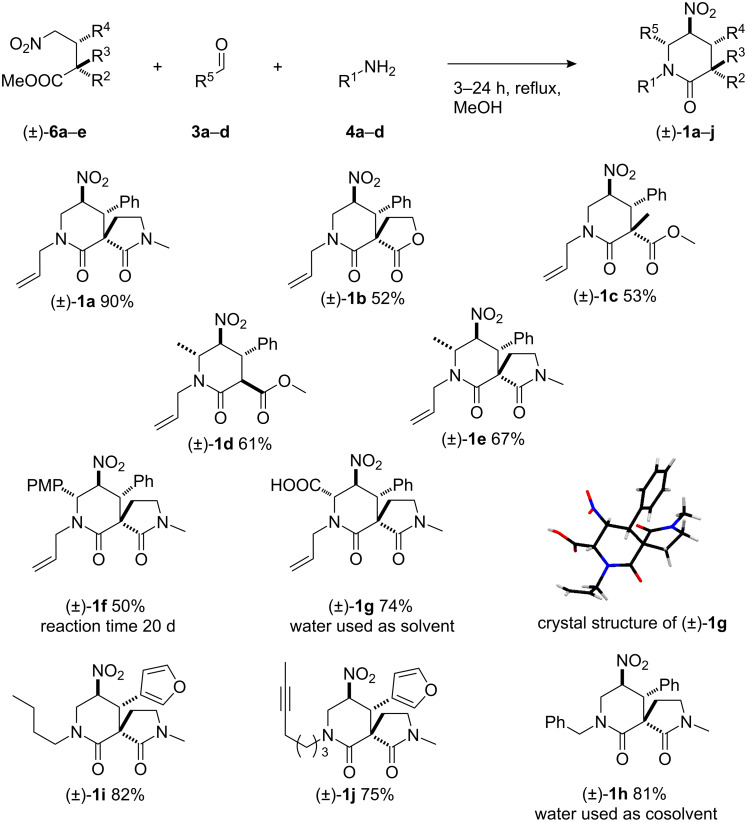
Nitro-Mannich/lactamisation cascade with in situ formed imines.

With a range of suitable test substrates in hand, formaldehyde-derived imines were then investigated in the nitro-Mannich/lactamisation reaction. Aqueous formaldehyde (**3a**) and allylamine (**4a**) were added to a methanol solution of lactam **6a** and the mixture heated under reflux for 4 hours until judged to be complete by TLC. Pleasingly, the desired δ-lactam product **1a** was isolated in 90% yield as a *single diasteromer* (Scheme 3) [84]. Under identical reaction conditions the other Michael adducts, lactone **6b** and ester **6c**, provided moderate yields of the desired δ-lactams **1b** and **1c** as single diastereoisomers in both cases. The diastereoselectivity in the latter case is notable, as the quaternary stereogenic centre is created in the lactamisation step. The relative stereochemical configurations of **1a–c** were established by ^1^H NMR spectroscopic analysis. For more details on the elucidation of the relative configuration see [61] and Supporting Information File 1. To incorporate substituents at the 6 position of the piperidine ring in **1**, imines derived from aldehydes other than formaldehyde were required in the reaction. Thus acetaldehyde (**3b**), anisaldehyde (**3c**) and glyoxylic acid (**3d**) were chosen as representative aliphatic, aromatic and functionalised aldehydes, respectively, and reacted with Michael adducts **6a** and **6d** under the conditions described above with allylamine. High diastereoselectivities were observed in each case and the reaction products **1d–g** were obtained in moderate to good yields (50–74%). The relative stereochemistry of **1g** was assigned unambiguously by single-crystal X-ray analysis. Similarly, variation at position 1 required the use of an alternative amine for in situ imine formation. Thus replacement of allylamine (**4a**) with benzylamine (**4b**) in the reaction afforded the desired product **1h** in good yield and as a single diastereoisomer (Scheme 3).

The use of substrate **6e** allowed us to investigate further variations at positions 1 and 4; piperidin-2-ones **1i** and **1j** were formed as single diastereoisomers in good yields (82% and 75%) when nitro-Mannich/lactamisation cascades were carried out with formaldehyde (**3a**) and butylamine (**4c**) or hept-5-yn-1-amine (**4d**), respectively. To extend the cascade methodology to the potential construction of architecturally complex piperidine-ring-containing polycyclic natural products, the successful employment of preformed cyclic imines was required.

**Figure 2:**
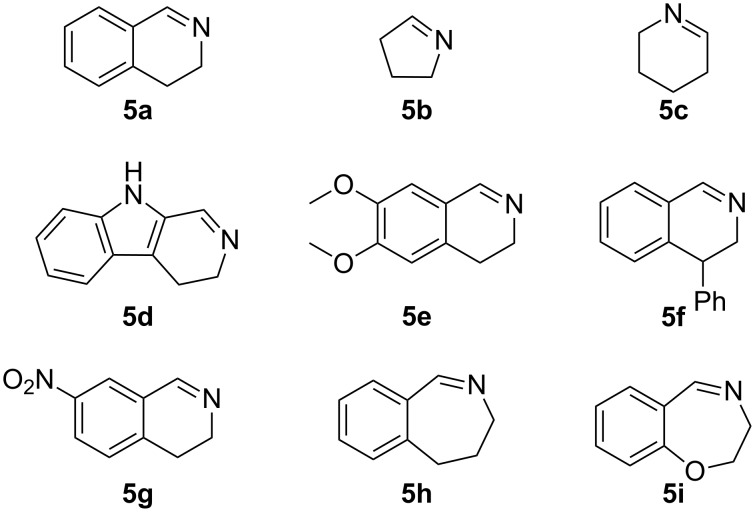
Cyclic imines employed in nitro-Mannich/lactamisation cascade.

**Scheme 4:**
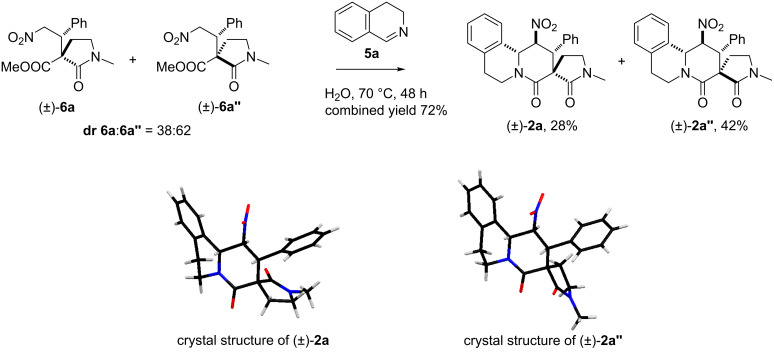
Nitro-Mannich/lactamisation cascade of diastereomeric Michael adducts **6a**, **6a’’** with cyclic imine **5a**.

Accordingly, the imine **5a** (Figure 2) was synthesised from commercially available 2-phenylethylamine [85] and reacted with the chromatographically inseparable mixture of diastereomeric Michael adducts **6a** and **6a’’**, under slightly modified conditions (water was used instead of MeOH as the solvent). Pleasingly the reaction proceeded smoothly and only two, **2a** and **2a’’**, of the possible eight diastereoisomeric tetracyclic compounds were obtained in good combined yield (70%, Scheme 4). Chromatographic separation followed by single-crystal X-ray diffraction studies of both isomers allowed unambiguous determination of the relative stereochemical configurations in each case. For more details of the elucidation of the relative configuration see Supporting Information File 1. The products were epimeric *only* at the quaternary centre and therefore both new stereogenic centres were created with high stereocontrol in each case.

**Scheme 5:**
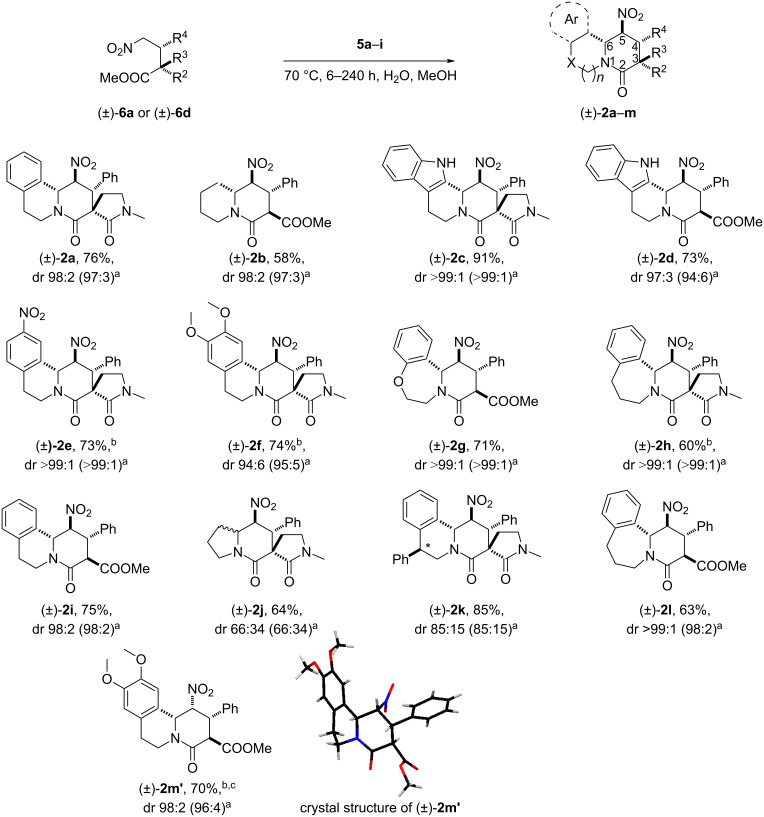
Nitro-Mannich/lactamisation cascade with cyclic imines. ^a^Diastereomeric ratio in a crude reaction mixture, ^b^H_2_O/MeOH 1:1 mixture used as a solvent, ^c^minor diastereomer **2m** isolated in 5% yield.

Imines **5a–5i** [61,86–94], chosen so as to afford common target motifs in the products [95–102], were synthesised and reacted with diastereomerically pure Michael adduct **6a** and Michael adduct **6d** following the conditions described above. Employing the optimal reaction conditions, products **2a**–**2l**, which possess 4,5*-*trans relative stereochemistry, were formed in moderate to good yields and with high diastereoselectivities as described in our previous work (Scheme 5) [61].

**Scheme 6:**
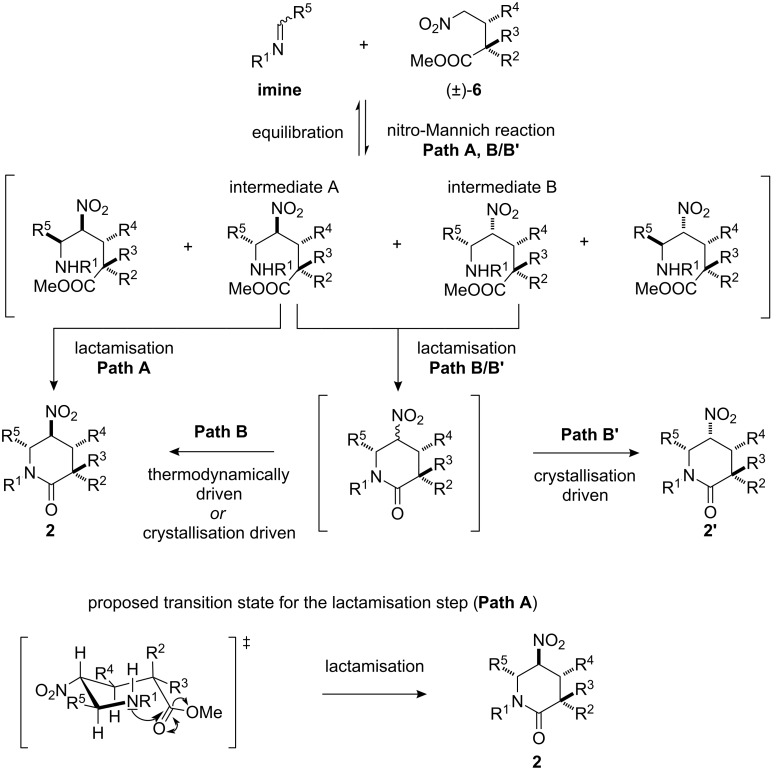
Possible explanations for the observed high stereoselectivities in the nitro-Mannich/lactamisation cascade.

Interestingly, however, when diastereomerically pure Michael adduct **6d** was reacted with imine **5e**, the nitropiperidinone **2m’**, possessing 4,5-cis relative stereochemistry [103], was isolated in 70% yield as a single diastereomer (Scheme 5). This one exceptional case together with the generally high diastereocontrol in the formation of piperidinones **1a–j** and **2a–l** is interesting and worthy of further commentary. With the knowledge that the retro-Michael reaction does not occur under standard reaction conditions (Scheme 4) and assuming that the final step of the cascade (the δ-lactam ring formation) is irreversible, there are at least three possible explanations for the high diastereocontrol in the formation of **1a–j** and **2a–l**:

- The first is that the nitro-Mannich step is highly diastereoselective and lactamisation occurs subsequently without any effect on the stereochemical outcome of the cascade.

- The second is that the nitro-Mannich reaction [78–80] is fast and reversible (but not necessarily stereoselective), and only one of the diastereomeric nitro-Mannich products preferentially cyclises in the irreversible lactamisation step to the (likely) most thermodynamically stable product (Scheme 6, Path A).

- The third is similar to the second, but the two direct nitro-Mannich products A and B with the observed configurations at the 6 position preferentially lactamise, and there is a postcyclisation epimerisation at the stereogenic carbon bearing the nitro group allowing equilibration to the (likely) most thermodynamically stable product (thermodynamic control, Path B) or a crystallisation-induced diastereoselectivity to give products **2** or **2’** with the nitro group occupying an axial or equatorial position, respectively [104–108] (Scheme 6, Path B/B’).

A further scrutiny of each hypothesis was, unfortunately, hampered by our failure to isolate or identify in situ the direct nitro-Mannich products from the reaction mixtures or to prepare them separately using standard procedures for a nitro-Mannich reaction with imines [109]. The first hypothesis, however, was not supported by the low diastereoselectivity in the formation of **2j**, in which presumably the relatively fast irreversible cyclisation outcompetes the equilibration processes. Considering the relatively broad range of imines and Michael adducts involved in the stereoselective cascade, we believe that the second or third explanations are the most plausible and that the observed diastereoselectivities in the formation of products **1a–j**, **2a–l** can be explained by following either Path A or B (Scheme 6).

**Scheme 7:**
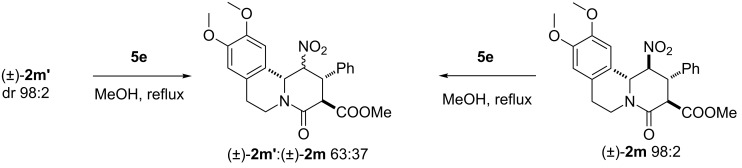
Thermodynamically-driven epimerisation of 5-nitropiperidin-2-ones **2m** and **2m’**.

**Figure 3:**
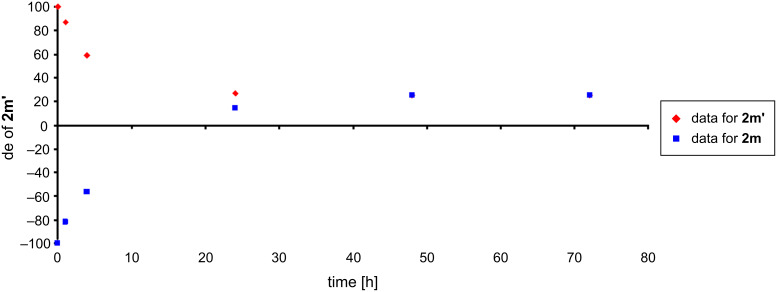
Thermodynamically driven epimerisation of 5-nitropiperidin-2-ones **2m** and **2m’**; identical diastereomeric excess measured for both diastereomers after 48 h and 72 h.

The formation of product **2m’** with its exceptional 4,5-*cis* relative stereochemistry, can be explained by following path B’ (Scheme 6). In this case the observed diastereoselectivity is believed to be driven by preferential crystallisation of the 4,5-*cis*-configured diastereoisomer in the reaction flask rather than thermodynamic equilibration. As such, this reaction represents an example of a crystallisation-induced diastereomeric transformation (CIDT) [104–108]. This is supported by the observation that **2m** and **2m’**, when exposed separately to simulated reaction conditions, epimerised at C5 to afford an identical 63:37 thermodynamic mixture of **2m**/**2m’**(Scheme 7; Figure 3) [110].

**Scheme 8:**
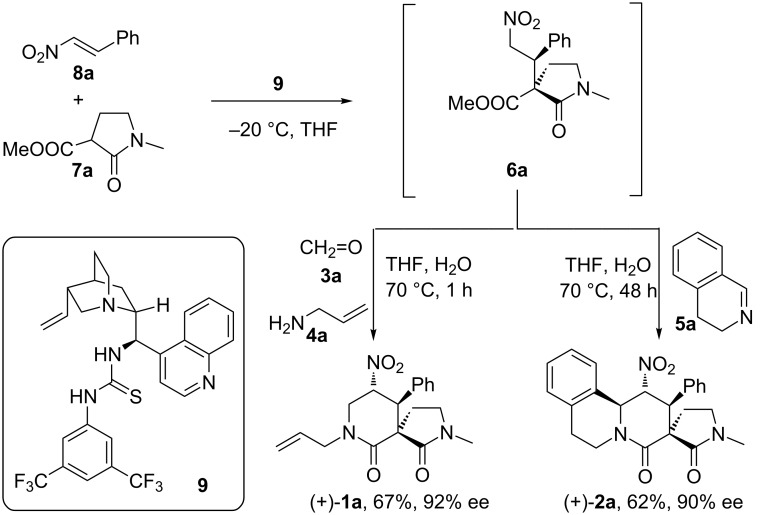
One-pot three/four-component enantioselective Michael addition/nitro-Mannich/lactamisation cascade.

With all of the necessary variations to the nitro-Mannich/lactamisation cascade having been tested, optimised and scoped, we looked at the possibility of combining it with a catalytic asymmetric synthesis of a particular Michael adduct, so as to construct a one-pot enantio- and diastereoselective four-component coupling reaction (Scheme 8).

As described in our previous communication [61], the employment of bifunctional catalyst **9** [54–55] in a highly stereoselective two-stage one-pot cascade led to the formation of enantiomerically highly enriched spirocycle (+)-**1a** (Scheme 8). In a repeat of the process but with the intention of targeting a piperidin-2-one ring-containing polycyclic scaffold, cyclic imine **5a** was added at the second stage. Tetracyclic spiro-lactam **2a** was isolated in high enantiomeric purity (90% ee) in good chemical yield (62%, Scheme 8) [111–112].

**Scheme 9:**
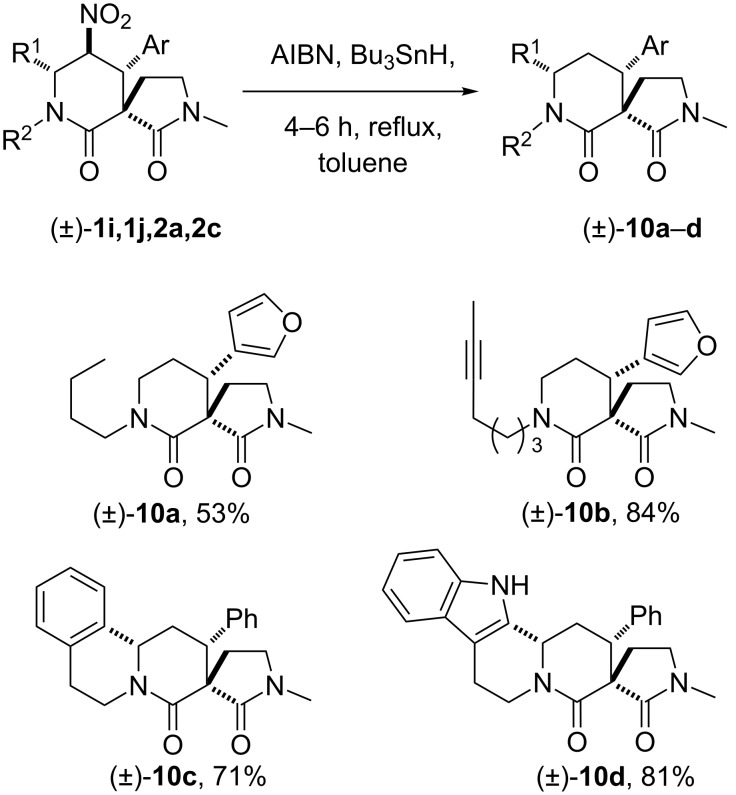
Protodenitration of 5-nitropiperidin-2-ones.

**Scheme 10:**
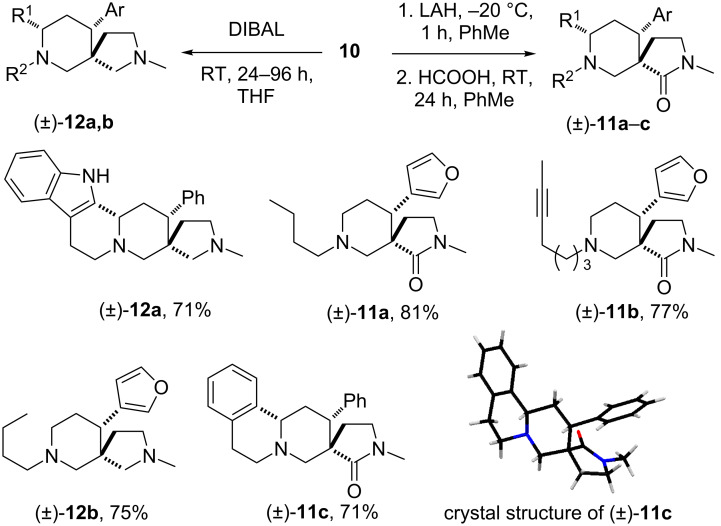
Various reductions of denitrated heterocycles.

For the products of the nitro-Mannich/lactamisation cascade to be of use in alkaloid natural-product synthesis (or even simple stereoselective piperidine synthesis), controlled, reductive manipulation of both the nitro group and the lactam carbonyl were required. Although Nef-type oxidation followed by exhaustive reduction of the resulting carbonyl group was considered, Ono’s radical procedure [113–116] was initially investigated. With some modification and optimisation, this was found to be compatible with the piperidin-2-one scaffold. Thus treatment of **2a** and **2c** with tributyltin hydride and AIBN in toluene under reflux smoothly afforded the protodenitrated products **10c** and **10d** in good yield (average 76% yield, Scheme 9). Other examples of successful nitro-group removal were also achieved when substrates **1i** and **1j**, lacking additional rings but bearing sensitive moieties (triple bond and furan moiety), were exposed to identical reaction conditions. The piperidin-2-ones **10a** and **10b** were obtained in 53% and 84%, respectively. The reduction of both piperidin-2-one and pyrrolidin-2-one heterocycles to piperidine or pyrrolidine rings by using a range of reagents is well-documented in the literature [117–118]. However, we believed that a controlled, chemoselective reduction would offer more options in any synthesis, and thus several commercially available reducing agents were screened in order to achieve selective reduction of only one lactam carbonyl. A notable find was that, by short exposure of denitrated heterocycle **10a–c** to LiAlH_4_ in THF followed by quenching and treatment with HCOOH, spirocycles **11a–c** were obtained in good yields. The chemoselectivity of the reduction was unambiguously confirmed by single-crystal X-ray diffraction studies of **11c**. Furthermore, the use of an excess of DIBAL at room temperature smoothly afforded the diamines **12a** and **12b** (Scheme 10).

## Conclusion

In summary, a versatile nitro-Mannich/lactamisation cascade for the direct synthesis of heavily decorated 5-nitropiperidin-2-ones and related heterocycles has been developed. A highly enantioenriched substituted 5-nitropiperidin-2-one was synthesised in a four-component one-pot cascade combining an enantioselective Michael addition with the diastereoselective nitro-Mannich/lactamisation cascade. Protodenitration and chemoselective reductive manipulation of the heterocycles could be used to install contiguous and fully substituted stereocentres in the synthesis of architecturally complex multicyclic alkaloid structures. The first applications of the developed methodology were disclosed recently as the total syntheses of paroxetine [60] and nakadomarin A [61–65] were successfully finished by employing the strategy as a fundamental synthetic tool. Further development is ongoing in our laboratory and the results will be disclosed in due course.

## Supporting Information

File 1 General experimental, copies of ^1^H and ^13^C NMR spectra for all new compounds (**1a–j, 2a, 2a’’, 2m, 2m’, 6b–e, 10a–d, 11a–c, 12a,b**).

File 2 X-ray crystal structure of compound **2m’**.

File 3 X-ray crystal structures of compounds **1g**, **2**, **2a’’**, **6e** and **11c**.
