# Supplementary material for: Stereoselective, nitro-Mannich/lactamisation cascades for the direct synthesis of heavily decorated 5-nitropiperidin-2-ones and related heterocycles
Source: Beilstein J Org Chem. 2012 Apr 16;8:567–78. doi: 10.3762/bjoc.8.64 (PMC3343283; doi:10.3762/bjoc.8.64)
Supplement: File 1 — General experimental, copies of 1H and 13C NMR spectra for all new compounds (1a–j, 2a, 2a’’, 2m, 2m’, 6b–e, 10a–d, 11a–c, 12a,b). [file Beilstein_J_Org_Chem-08-567-s001.pdf]

Supporting Information

for

Stereoselective, nitro-Mannich/lactamisation cascades for the direct synthesis of heavily decorated 5-nitropiperidin-2-ones and related heterocycles

Pavol Jakubec[1], Dane M. Cockfield[2], Madeleine Helliwell[2], James Raftery[2] and Darren J. Dixon[*1]

Address: [1]The Department of Chemistry, Chemistry Research Laboratory, University of Oxford, Mansfield Road, Oxford OX1 3TA, U.K. and [2]School of Chemistry, The University of Manchester, Oxford Road, Manchester, M13 9PL

E-mail: Darren Dixon - Darren.dixon@chem.ox.ac.uk

*Corresponding author

General experimental, copies of ^{1}H and ^{13}C NMR spectra for all new compounds

(**1a**–**j**, **2a**, **2a''**, **2m**, **2m'**, **6b**–**e**, **10a**–**d**, **11a**–**c**, **12a**,**b**)

Content

1. General experimental S4

1.1. Solvents and reagents S4
1.2. Chromatography S4
1.3. Spectroscopy S4
1.4. Single-crystal X-Ray structure determination S4
1.5. Starting material S5

2. Practical experimental S5

2.1. General procedure A for Michael addition of nucleophiles **7** to nitroolefin **8** S5
2.1.1. Synthesis and characterization of **6b** S5
2.1.2. Synthesis and characterization of **6c** S6
2.1.3. Synthesis and characterization of **6d** S6
2.1.4. Synthesis and characterization of **6e** S6
2.2. General procedure B for nitro-Mannich/lactamization cascade S7
2.2.1. Synthesis and characterization of **1a** S7
2.2.2. Enantioselective "one-pot" synthesis of (+)**-1a** and its characterization S8
2.2.3. Synthesis and characterization of **1b** S8
2.2.4. Synthesis and characterization of **1c** S9
2.2.5. Synthesis and characterization of **1d** S9
2.2.6. Synthesis and characterization of **1e** S10
2.2.7. Synthesis and characterization of **1f** S10
2.2.8. Synthesis and characterization of **1g** S11
2.2.9. Synthesis and characterization of **1h** S11
2.2.10. Synthesis and characterization of **1i** S12
2.2.11. Synthesis and characterization of **1j** S12
2.2.12. Synthesis and characterization of **2a** S13
2.2.13. Synthesis and characterization of **2a** and **2a''** S13
2.2.14. Synthesis and characterization of **2m** and **2m'** S14
2.3. General procedure C for the nitro-group removal S15
2.3.1. Synthesis and characterization of **10a** S15
2.3.2. Synthesis and characterization of **10b** S16
2.3.3. Synthesis and characterization of **10c** S17
2.3.4. Synthesis and characterization of **10d** S17
2.4. General procedure D for the chemoselective reduction of **10** S18
2.4.1. Synthesis and characterization of **11a** S18
2.4.2. Synthesis and characterization of **11b** S19
2.4.3. Synthesis and characterization of **11c** S19
2.4.4. Synthesis and characterization of **12a** S20
2.4.5. Synthesis and characterization of **12b** S20

3. Elucidation of relative configuration S21

3.1. nOes S21
3.1.1. nOes, compound **1c** S21
3.2. Single-crystal X-ray crystallography S22
3.2.1. X-ray crystal structure of compound **6e** S22
3.2.2. X-ray crystal structure of compound **1g** S24

3.2.3. X-ray crystal structure of compound **2a** S26
3.2.4. X-ray crystal structure of compound **2a’’** S28
3.2.5. X-ray crystal structure of compound **2m’** S30
3.2.6. X-ray crystal structure of compound **11c** S31

4. References S33

5. ^{1}H and ^{13}C NMR spectra S34

5.1. ^{1}H and ^{13}C NMR spectra of **6b** S34
5.2. ^{1}H and ^{13}C NMR spectra of **6c** S36
5.3. ^{1}H and ^{13}C NMR spectra of **6d** S38
5.4. ^{1}H and ^{13}C NMR spectra of **6e** S40
5.5. ^{1}H and ^{13}C NMR spectra of **1a** S42
5.6. ^{1}H and ^{13}C NMR spectra of **1b** S44
5.7. ^{1}H and ^{13}C NMR spectra of **1c** S46
5.8. ^{1}H and ^{13}C NMR spectra of **1d** S48
5.9. ^{1}H and ^{13}C NMR spectra of **1e** S50
5.10. ^{1}H and ^{13}C NMR spectra of **1f** S52
5.11. ^{1}H and ^{13}C NMR spectra of **1g** S54
5.12. ^{1}H and ^{13}C NMR spectra of **1h** S56
5.13. ^{1}H and ^{13}C NMR spectra of **1i** S58
5.14. ^{1}H and ^{13}C NMR spectra of **1j** S60
5.15. ^{1}H and ^{13}C NMR spectra of **2a** S62
5.16. ^{1}H and ^{13}C NMR spectra of **2a’’** S64
5.17. ^{1}H and ^{13}C NMR spectra of **2m** S66
5.18. ^{1}H and ^{13}C NMR spectra of **2m’** S68
5.19. ^{1}H and ^{13}C NMR spectra of **10a** S70
5.20. ^{1}H and ^{13}C NMR spectra of **10b** S72
5.21. ^{1}H and ^{13}C NMR spectra of **10c** S74
5.22. ^{1}H and ^{13}C NMR spectra of **10d** S76
5.23. ^{1}H and ^{13}C NMR spectra of **11a** S78
5.24. ^{1}H and ^{13}C NMR spectra of **11b** S80
5.25. ^{1}H and ^{13}C NMR spectra of **11c** S82
5.26. ^{1}H and ^{13}C NMR spectra of **12a** S84
5.27. ^{1}H and ^{13}C NMR spectra of **12b** S86

6. HPLC trace S88

6.1. HPLC trace of (±)-**1a** S88
6.2. HPLC trace of (+)-**1a** S89

1. General experimental

All reactions were performed under an atmosphere of dry nitrogen unless otherwise stated. All glass apparatus were oven dried and cooled under vacuum before use.

1.1. Solvents and reagents

Bulk solutions were evaporated under reduced pressure by using a Büchi rotary evaporator. Tetrahydrofuran and diethyl ether were distilled from sodium benzophenone ketyl radical. Dichloromethane and toluene were distilled from calcium hydride prior to use. Petroleum ether refers to the distilled light petroleum of fraction (40–65 °C). Reagents used were obtained from commercial suppliers and used without purification

1.2. Chromatography

Column chromatography was carried out by using Merck Kieselgel 60 silica gel (230–400 mesh). All reactions were followed by thin-layer chromatography (TLC) where practical, with Merck Kieselgel 60 F_{254} (230–400 mesh) fluorescent treated silica, which were visualised under UV light (250 nm) or by staining with aqueous acidic ammonium molybdate or aqueous basic potassium permanganate solutions, as appropriate.

1.3. Spectroscopy

All ^{1}H and ^{13}C NMR spectra were recorded on a Bruker 500, 400 MHz or Varian 300 MHz spectrometer and are quoted in ppm for measurements against a TMS internal standard. Unless otherwise stated all experiments were carried out in *d*-chloroform as solvent. Chemical shifts (δ) are given in parts per million (ppm), and coupling constants (*J*) are given in hertz (Hz). The ^{1}H NMR spectra are reported as follows: σ/ppm (multiplicity (s = singlet, d = doublet, t = triplet, q = quartet, sxt = sextet, m = multiplet, bs = broad singlet, "d" = apparent doublet, "dd" = apparent doublet of doublet, "dt" = apparent doublet of triplet), number of protons, coupling constants *J*/Hz (where appropriate) and assignment). DEPT 135 and two-dimensional (COSY, HMQC, HMBC) NMR spectroscopy were used where appropriate to assist the assignment of signals in the ^{1}H and ^{13}C NMR spectra. n.O.e experiments were used to determine the relative configuration. Low-resolution mass spectrometry (EI, CI) was recorded on a Fissions VG Trio 2000 quadrupole mass spectrometer. High-resolution mass spectra (accurate mass) were recorded on a Thermo Finnigan Mat 95XP mass spectrometer. Infrared spectra were recorded on an ATI Mattson: Genesis Series FTIR spectrometer from a thin film deposited onto a sodium chloride plate. Only selected maximum absorbances (ν_{max}) are reported. Optical rotations were recorded by using an Optical Activity AA-1000 polarimeter; $[\alpha]_D$ values are reported in 10^{-1} deg cm^2 g^{-1}; concentration (c) is given in g/100 mL at 589 nm.

1.4. Single-crystal X-ray structure determination

Diffraction data were collected at low temperature [1] on a Bruker SMART CCD diffractometer (**1g**, **2a** and **2a''**) or a Nonius Kappa CCD diffractometer (**6e** and **11c**); raw frames were integrated with SAINT [Bruker SMART, SADABS and SAINT, Bruker AXS Inc., Madison, Wisconsin, USA.] or DENZO-SMN [2] as appropriate. Inter-frame scaling and absorption corrections were carried out with SADABS [3] (**1g**, **2a** and **2a''**) or SCALEPACK (**6e** 18 and **11c**). The structures were solved by direct methods with SIR2004 [4] (**1g**), SHELXS [3] (**2a** and **2a''**) or SIR92 (**6e** and **11c**). Compounds **1g, 2a** and **2a''** were refined with SHELXL [3]; all nonhydrogen atoms were refined with anisotropic displacement parameters and hydrogen atoms were positioned geometrically. Compounds **6e** and **11c** were refined with the CRYSTALS suite [5].

CCDC-867211 (for **6e**), CCDC-867208 (for **1g**), CCDC-867209 (for **2a**), CCDC-867210 (for **2a''**), CCDC-867207 (for **2m'**), CCDC-867212 (for **11c**), contain the supplementary crystallographic data for this paper. These data can be obtained free of charge from The Cambridge Crystallographic Data Centre via www.ccdc.cam.ac.uk/data_request/cif.

1.5. Starting materials

Electrophile **8b** (Ar = furan-2-yl) [6], nucleophiles **7a** [7], **7b** [8], Michael adduct **6a** [7], cyclic imines **5a** [9], **5e** [10], amine **4d** [11], nitro-Mannich /lactamization products, **2a-k** [7], and catalyst **9** [12] were prepared according to literature procedures and our previously published procedures. Nucleophiles **7c**, **7d,** nitroolefin **8a** are commercially available.

2. Practical experimental

2.1. General procedure A for Michael addition of nucleophiles 7 to nitroolefin 8

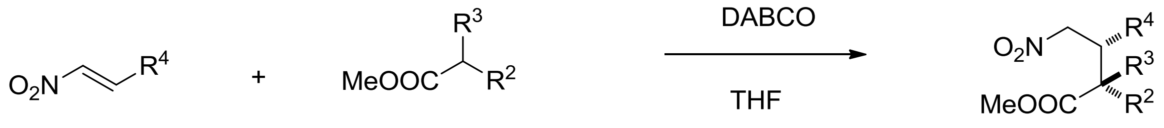

8 (±)-**7** (±)-**6**

A solution of nitroolefin **8**, nucleophile **7** and DABCO in THF was stirred at RT until TLC analysis showed that all starting material had been consumed. The mixture was then concentrated in vacuo and the crude product was purified by flash column chromatography to yield the title compound **6**.

2.1.1. Synthesis and characterization of 6b

(±)-Methyl (3*S*)-3-[(1*R*)-2-nitro-1-phenylethyl]-2-oxotetrahydrofuran-3-carboxylate (**6b**)

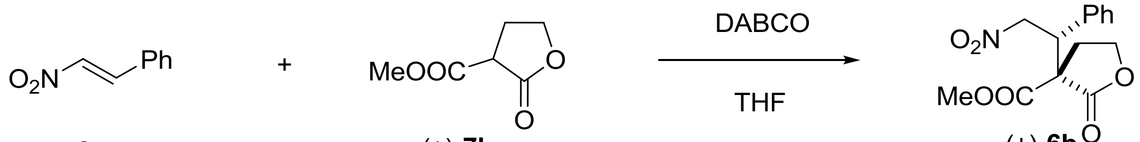

8a (±)-**7b** (±)-**6b**

According to the general procedure A (for 0.67 mmol, 0.100 g of **8a** used 0.74 mmol, 0.106 g of **7b**; 0.13 mmol, 0.015 g of DABCO and 1.3 mL of THF; 24 h) (±)-**6b** (0.173 g, 88%, dr 65:35) was obtained after column chromatography (Et_2O) as a colourless solid. Recrystallization of the mixture of diastereomers (Et_2O) afforded the single diastereomer (±)-**6b** as a colourless solid.
Mp 105–106 °C; IR (film) 2958 (C-H), 1770 (C=O), 1739 (C=O), 1556, 1380 (NO_2); ^{1}H NMR (500 MHz, $CDCl_3$) δ 2.27–2.33 (m, 1H, $C\underline{H_A}H_BCH_2O$), 2.52–2.57 (m, 1H, $CH_A\underline{H_B}CH_2O$), 3.63 (dt, 1H, *J* = 8.8, 4.4 Hz, $CH_2C\underline{H_A}H_BO$), 3.86 (s, 3H, $OC\underline{H_3}$), 4.16–4.21 (m, 1H, $CH_2CH_A\underline{H_B}O$), 4.31 (dd, 1H, *J* = 11.0, 3.5 Hz, $C\underline{H}Ph$), 4.98 (dd, 1H, *J* = 13.9, 3.5 Hz, $C\underline{H_A}H_BNO_2$), 5.28 (dd, 1H, *J* = 13.9, 11.0 Hz, $CH_A\underline{H_B}NO_2$), 7.29–7.39 (m, 5H, $\underline{H}$-Ar); ^{13}C NMR (125 MHz, $CDCl_3$) δ 31.8 ($\underline{C}H_2CH_2O$), 46.5 ($\underline{C}HPh$), 53.7 ($\underline{C}H_3O$), 56.8 (C_{quat}), 66.2 ($CH_2\underline{C}H_2O$), 76.5 ($\underline{C}H_2NO_2$), 128.9, 129.0, 129.3, 134.3 ($\underline{C}$-Ar), 169.7, 173.8 ($\underline{C}$=O); HRMS–ES+ *m*/*z*: $[M + NH_4]^+$ calculated for $C_{14}H_{19}N_2O_6$, 311.1238; found, 311.1231.

2.1.2. Synthesis and characterization of 6c

(±)-Dimethyl methyl(2-nitro-1-phenylethyl)propanedioate (**6c**)

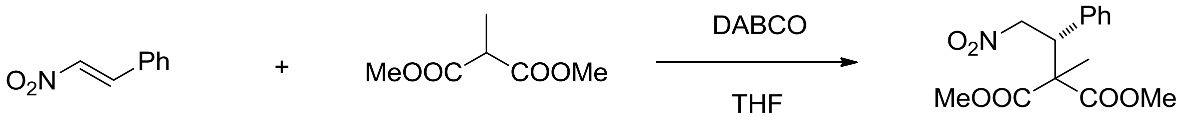

8a (±)-**7c** (±)-**6c**

According to general procedure A (for 6.7 mmol, 1.000 g of **8a** used 20.1 mmol, 2.937 g, 2.675 mL of methyl dimethylmalonate **7c**; 1.3 mmol, 0.150 g of DABCO and 6.7 mL of THF; 6 days). (±)-**6c** (1.381 g, 70%) was obtained after flash column chromatography (Et_2O/PE 1:2) as a colourless solid. Mp 106–107 °C. All spectral data are in agreement with those published in the literature [13,14].

2.1.3. Synthesis and characterization of 6d

(±)-Dimethyl (2-nitro-1-phenylethyl)propanedioate (**6d**)

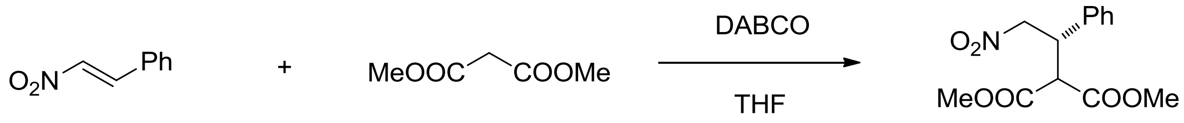

8a (±)-**7d** (±)-**6d**

According to general procedure A (for 6.7 mmol, 1.000 g of **8a** used 20.1 mmol, 2.655 g, 2.297 mL of **7d**; 1.3 mmol, 0.150 g of DABCO and 6.7 mL of THF; 2 days). **6d** (1.623 g, 86%) was obtained after flash column chromatography (PE/Et_2O 1:1) as a colourless solid.
Mp 60–61 °C (lit. [15]: mp 63 °C); all spectral data are in agreement with those published in the literature [13].

2.1.4. Synthesis and characterization of 6e

(±)-Methyl (3*S*)-3-[(1*R*)-1-(furan-3-yl)-2-nitroethyl]-2-oxotetrahydrofuran-3-carboxylate (**6e**)

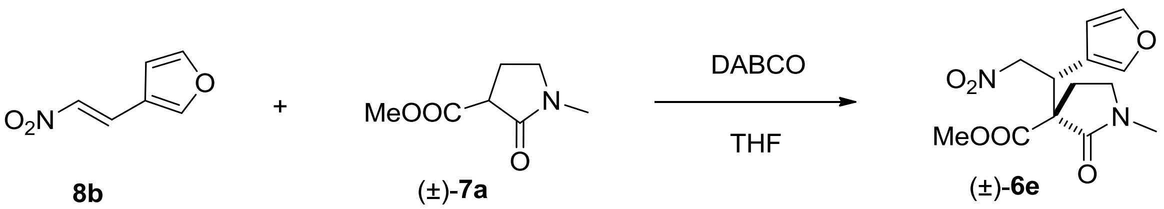

8b (±)-**7a** (±)-**6e**

According to the general procedure A (for 32.3 mmol, 4.500 g of **8b** used 42.1 mmol, 6.609 g of **7a**; 9.7 mmol, 1.088 g of DABCO and 45 mL of THF; 72 h; the reaction was performed with exclusion of light). **6e** (5.253 g, 55%, dr 62:38) was obtained after column chromatography ($Et_2O \rightarrow Et_2O$/AcOEt 80:20) as an off-white solid. Recrystallisation of the mixture of diastereomers (EtOH) afforded the single diastereomer (±)-**6e** as a colourless solid.
Mp 116–117 °C; IR (film) 2956, 2889 (C-H), 1735 (C=O), 1688 (C=O), 1553, 1380 (NO_2); ^1H NMR (500 MHz, $CDCl_3$) δ 2.08–2.14 (m, 1H, $C\underline{H}_{A}H_{B}CH_{2}N$), 2.32 (ddd, 1H, *J* = 13.3, 8.8, 4.4 Hz, $CH_{A}\underline{H}_{B}CH_{2}N$), 2.81–2.85 (m, 4H, $CH_{2}C\underline{H}_{A}H_{B}N$, $C\underline{H}_{3}N$), 3.31 ("dt", 1H, *J* = 9.0, 6.0 Hz, $CH_{2}CH_{A}\underline{H}_{B}N$), 3.81 (s, 3H, $C\underline{H}_{3}O$), 4.19 (dd, 1H, *J* = 11.0, 3.2 Hz, $C\underline{H}CH_{A}H_{B}NO_{2}$), 4.89 (dd, 1H, *J* = 13.2, 3.2 Hz, $CHC\underline{H}_{A}H_{B}NO_{2}$), 5.10 (dd, 1H, *J* = 13.2, 11.0 Hz, $CHCH_{A}\underline{H}_{B}NO_{2}$), 6.29 (s, 1H, $\underline{H}$-Ar), 7.38–7.39 (m, 2H,

H-Ar); ^{13}C NMR (125 MHz, $CDCl_3$) δ 28.3 (CH_2CH_2N), 30.1 (CH_3N), 38.5 (CHAr), 46.8 (CH_2CH_2N), 53.2 (CH_3O), 57.3 (C_{quat}), 77.1 (CH_2NO_2), 109.3, 119.5, 141.8, 143.8 (C-Ar), 170.4, 171.6 (C=O); HRMS–ES+ (*m*/*z*): $[M + Na]^+$ calculated for $C_{13}H_{16}N_2NaO_6$, 319.0925; found, 319.0918.

2.2. General procedures B for nitro-Mannich / lactamisation cascade

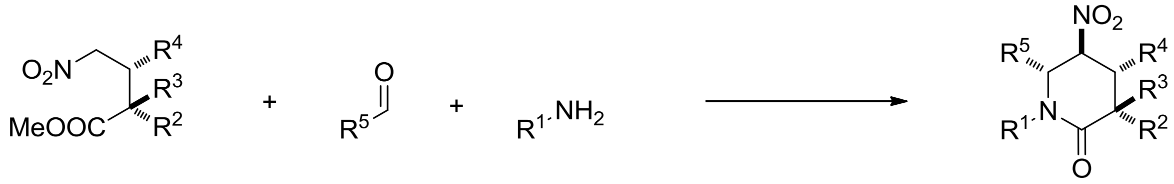

(±)-**6a-e** **3a-d** **4a-d** (±)-**1a-j**

Michael adduct **6** (0.49 mmol) was placed in a suitable solvent (MeOH or water, 3.5 mL, c = 0.14 M) at rt. To the mixture was added amine **4** (2 equiv, 0.98 mmol) and aldehyde **3** (2 equiv, 0.98 mmol), and the mixture was heated under reflux in MeOH (at 70 °C in water) until the starting material had been consumed by TLC analysis. The mixture was then concentrated in vacuo and subjected to flash column chromatography.

2.2.1. Synthesis and characterization of 1a

(±)-(5*R*,9*S*,10*R*)-2-Methyl-9-nitro-10-phenyl-7-(prop-2-en-1-yl)-2,7-diazaspiro[4.5]decane-1,6-dione (**1a**)

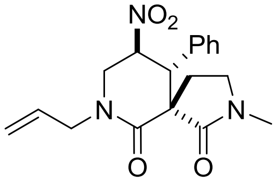

(±)-**1a**

According to the general procedure B (for 0.49 mmol, 150 mg of **6a** used 0.98 mmol, 74 μL of allylamine (**4a**); 0.98 mmol, 74 μL of formaldehyde (**3a**, 37% solution in water) and 3.5 mL of MeOH; under reflux; 4 h). **1a** (153 mg, 90%) was obtained as a colourless solid after flash column chromatography (Et_2O).

Mp 145–146 °C; IR (film) 2923 (C-H), 1680 (C=O), 1650 (C=O), 1551, 1351 (NO_2); ^{1}H NMR (500 MHz, $CDCl_3$) δ 1.80–1.85 (m, 1H, $CH_AH_BCH_2N$), 2.07–2.12 (m, 1H, $CH_2CH_AH_BN$), 2.58 (s, 3H, CH_3N), 2.91–2.97 (m, 1H, $CH_AH_BCH_2N$), 3.09–3.14 (m, 1H, $CH_2CH_AH_BN$), 3.58 (d, 1H, J = 11.7 Hz, PhCHCH), 3.72 (dd, 1H, J = 12.0, 8.8 Hz, $CH_AH_BCHNO_2$), 3.96 (dd, 1H, J = 15.1, 5.7 Hz, $CH_ACH_BCH{=}CH_2$), 4.07 (dd, 1H, J = 12.0, 6.6 Hz, $CH_AH_BCHNO_2$), 4.12 (dd, 1H, J = 15.1, 5.7 Hz, $CH_ACH_BCH{=}CH_2$), 5.21–5.28 (m, 2H, $CH{=}CH_2$), 5.69–5.77 (m, 1H, $CH{=}CH_2$), 6.27 (ddd, 1H, J = 11.7, 8.8, 6.6 Hz, $CHNO_2$), 7.20–7.26 (m, 5H, H-Ar); ^{13}C NMR (125 MHz, $CDCl_3$) δ 27.0 (CH_2CH_2N), 29.8 (CH_3N), 47.0 (CH_2CH_2N), 48.9 (CH_2CHNO_2), 50.0 ($CH_2CH{=}CH_2$), 51.0 (CHPh), 56.2 (C_{quat}), 81.5 ($CHNO_2$), 118.4, 128.6, 128.7, 131.0, 133.9 (C-Ar, $CH_2CH{=}CH_2$), 167.8, 171.1 (C=O); HRMS–ES+ (*m*/*z*): $[M + Na]^+$ calculated for $C_{18}H_{21}N_3NaO_4$, 366.1424; found, 366.1436; Anal. calcd for $C_{18}H_{21}N_3O_4$: C, 62.96; H, 6.16; N, 12.24; found: C, 62.87; H, 6.22; N, 12.17%.

2.2.2. Enantioselective "one-pot" synthesis of (+)-1a and its characterization

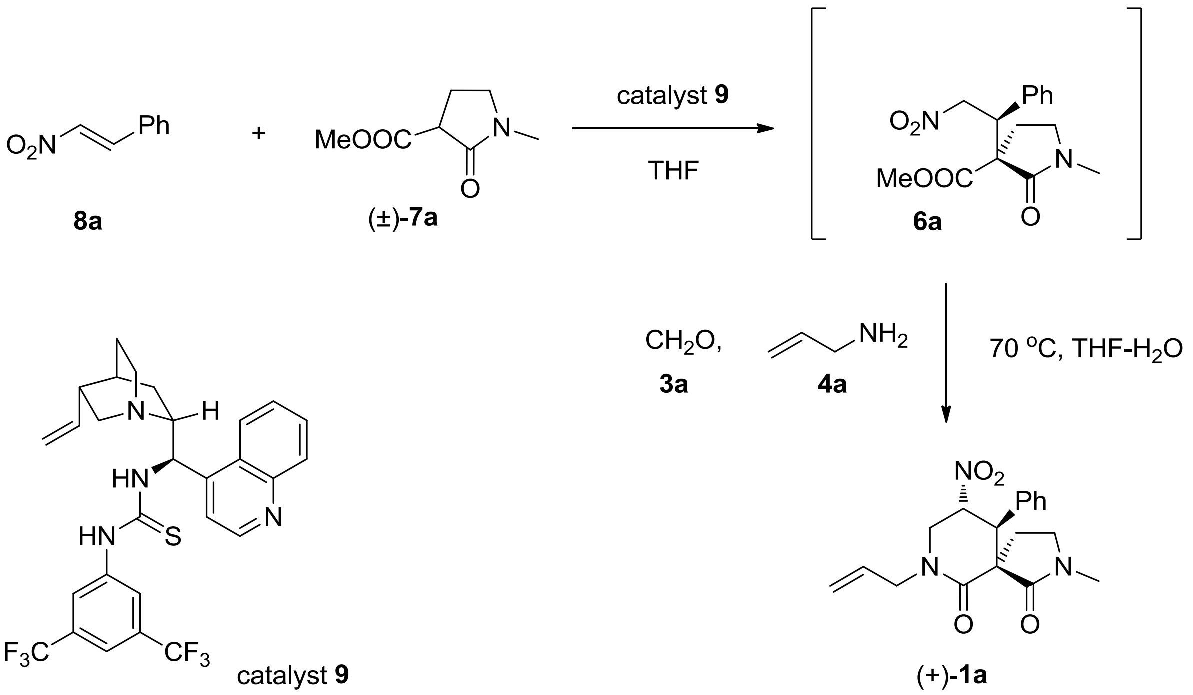

A solution of nitrostyrene **8a** (0.67 mmol, 0.100 g), ester **7a** (1.3 equiv, 0.87 mmol, 0.137 g) and catalyst **9** (0.2 equiv, 0.13 mmol, 0.076 g) in THF (1 mL) was stirred at −20 °C. After starting material had been consumed (TLC monitoring, 14 d) water (4 mL), formaldehyde (**3a**, 2 equiv, 1.34 mmol, 101 μL of 37% solution in water) and allylamine (**4a**, 2 equiv, 1.34 mmol, 101 μL) were added and the mixture was stirred at 70 °C. After 1 h the mixture was cooled to rt and concentrated in vacuo. The crude product was purified by flash column chromatography (Et_2O) to obtain (+)-**1a** as a colourless solid (0.153 g, 67%, 92% ee). Recrystallization (MeOH/Et_2O) afforded (+)-**1a** in 98% ee.

For (+)-**1a**, 98% ee the spectroscopic data was identical to that of the racemate. Mp 128–129 °C, $[\alpha]_D^{25}$ = + 24.4 (*c* 0.6, $CHCl_3$).

2.2.3. Synthesis and characterization of 1b

(±)-(5*S*,9*S*,10*R*)-9-Nitro-10-phenyl-7-(prop-2-en-1-yl)-2-oxa-7-azaspiro[4.5]decane-1,6-dione (**1b**)

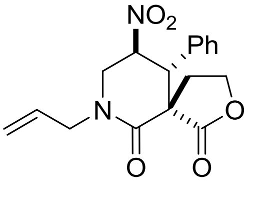

(±)-**1b**

According to the general procedure B (for 0.34 mmol, 0.100 g of **6b** used 0.61 mmol, 46 μL, of allylamine (**4a**); 0.61 mmol, 46 μL, of formaldehyde (**3a**, 37% solution in water) and 2.5 ml of MeOH; under reflux; 8 h). **1b** (0.058 g, 52%) was obtained after column chromatography (Et_2O) as a colourless solid. Mp 135–137 °C; IR (film) 2924 (C-H), 1756 (C=O), 1651 (C=O), 1556, 1351 (NO_2); ^1H NMR (500 MHz, $CDCl_3$) δ 2.14–2.19 (m, 1H, C$H_A$$H_BCH_2$O), 3.12–3.18 (m, 1H, C$H_A$$H_BCH_2$O), 3.42–3.47 (m, 1H, C$H_2CH_A$$H_B$O), 3.74 (d, 1H, *J* = 11.7 Hz, CHPh), 3.82 (dd, 1H, *J* = 12.6, 8.2 Hz, C$H_A$$H_BCHNO_2$), 4.02 (dd, 1H, *J* = 15.1, 6.0 Hz, NC$H_A$$H_B$CH=C$H_2$), 4.17–4.22 (m, 2H, NC$H_A$$H_B$CH=C$H_2$, C$H_A$$H_BCHNO_2$), 4.31–4.36 (m, 1H, C$H_2CH_A$$H_B$O), 5.29–5.32 (m, 2H, C$H_2$=CH), 5.75–5.82 (m, 1H, CH=CH_2), 6.18 (ddd, 1H, *J* = 11.7, 7.9, 7.1 Hz, CHNO_2), 7.29–7.42 (m, 5H, H-Ar); ^{13}C NMR (125 MHz,

$CDCl_3$) δ 30.7 ($\underline{C}H_2CH_2O$), 48.8 ($\underline{C}H_2CHNO_2$), 50.0 ($\underline{C}H_2CH{=}CH_2$), 50.3 ($\underline{C}HPh$), 55.3 ($C_{quat}$), 67.6 ($CH_2\underline{C}H_2O$), 81.8 ($\underline{C}HNO_2$), 119.1, 128.6, 129.4, 129.5, 130.8, 133.4 ($CH_2\underline{C}H{=}CH_2$, $\underline{C}$-Ar), 165.7, 174.8 ($\underline{C}{=}O$); HRMS–ES+ (*m*/*z*): $[M + Na]^+$ calcd for $C_{17}H_{18}N_2NaO_5$, 353.1108; found, 353.1105.

2.2.4. Synthesis and characterization of 1c

(±)-Methyl (3*S*,4*R*,5*S*)-3-methyl-5-nitro-2-oxo-4-phenyl-1-(prop-2-en-1-yl)piperidine-3-carboxylate (**1c**)

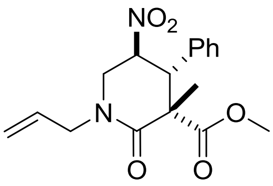

(±)-**1c**

According to the general procedure B (for 1.70 mmol, 0.500 g of **6c** used 2.20 mmol, 165 µL, of allylamine (**4a**); 2.20 mmol, 165 µL, of formaldehyde (**3a**, 37% solution in water) and 12.0 mL of MeOH; under reflux; 4 h). **1c** (0.297 g, 53%) was obtained after column chromatography (Et_2O) as a colourless solid. Mp 86–88 °C; IR (film) 2950 (C-H), 1750 (C=O), 1649 (C=O), 1556, 1350 (NO_2); ^1H NMR (500 MHz, $CDCl_3$) δ 1.43 (s, 3H, $C_{quat}C\underline{H_3}$), 3.51–3.53 (m, 4H, $C\underline{H_3}O$, $C\underline{H}Ph$), 3.78 (dd, 1H, *J* = 12.0, 9.8 Hz, $C\underline{H_A}H_BCHNO_2$), 3.86–3.91 (m, 2H, $CH_A\underline{H_B}CHNO_2$, $C\underline{H_A}H_BCH{=}CH_2$), 4.16 (dd, 1H, *J* = 15.3, 5.5 Hz, $CH_A\underline{H_B}CH{=}CH_2$), 5.21–5.24 (m, 2H, $CH{=}C\underline{H_2}$), 5.68–5.75 (m, 1H, $C\underline{H}{=}CH_2$), 5.90 (ddd, 1H, *J* = 12.3, 9.8, 6.0 Hz, $C\underline{H}NO_2$), 7.06–7.08 (m, 2H, $\underline{H}$-Ar), 7.20–7.26 (m, 3H, $\underline{H}$-Ar); ^{13}C NMR (125 MHz, $CDCl_3$) δ 21.1 ($\underline{C}H_3$), 49.0 ($\underline{C}H_2CHNO_2$), 49.9 ($\underline{C}H_2CH{=}CH_2$), 52.4 ($C\underline{H_3}O$), 52.7 ($C\underline{H}Ph$), 55.2 ($C_{quat}$), 81.4 ($\underline{C}HNO_2$), 118.6, 128.3, 128.8, 131.1, 133.5 ($\underline{C}H_2{=}\underline{C}H$, $\underline{C}$-Ar), 167.3, 170.7 ($\underline{C}{=}O$); HRMS–ES+ (*m*/*z*): $[M + H]^+$ calculated for $C_{17}H_{21}N_2O_5$, 333.1445; found, 333.1454.

2.2.5. Synthesis and characterization of 1d

(±)-Methyl (3*R*,4*S*,5*S*,6*R*)-6-methyl-5-nitro-2-oxo-4-phenyl-1-(prop-2-en-1-yl)piperidine-3-carboxylate (**1d**)

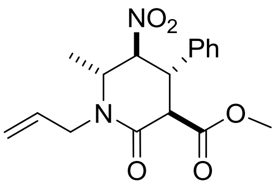

(±)-1d

According to the general procedure B (for 0.49 mmol, 0.138 g of **6d** used 2.20 mmol, 0.126 g, 165 µL of allylamine (**4a**); 2.20 mmol, 0.097 g, 123 µL, of acetaldehyde (**3b**) and 3.8 mL of MeOH; under reflux; 4 h). **1d** (0.099 g, 61%) was obtained after column chromatography (Et_2O) as a colourless solid.
Mp 115–116 °C; IR (film) 2953 (C-H), 1746 (C=O), 1648 (C=O), 1557, 1350 (NO_2); ^1H NMR (500 MHz, $CDCl_3$) δ 1.38 (d, 3H, *J* = 6.3 Hz, $C\underline{H_3}CH$), 3.51 (s, 3H, $C\underline{H_3}O$), 3.65 (d, 1H, *J* = 12.9 Hz, $C\underline{H}COOCH_3$), 3.75 (dd, 1H, *J* = 15.1, 7.3 Hz, $C\underline{H_A}CH_BCH{=}CH_2$), 4.00 (dd, 1H, *J* = 12.9, 11.0 Hz, $C\underline{H}Ph$), 4.04–4.10 (m, 1H, $CH_3C\underline{H}$), 4.39–4.44 (m, 1H, $CH_AC\underline{H_B}CH{=}CH_2$), 4.73 (dd, 1H, *J* = 11.0, 7.6 Hz, $C\underline{H}NO_2$), 5.20–5.24 (m, 2H, $CH_2CH{=}C\underline{H_2}$), 5.71–5.79 (m, 1H, $CH_2C\underline{H}{=}CH_2$), 7.11–7.14 (m, 2H, $\underline{H}$-Ar), 7.22–7.29 (m, 3H, $\underline{H}$-Ar); ^{13}C NMR (125 MHz, $CDCl_3$) δ 19.6 ($\underline{C}H_3CH$), 45.1 ($\underline{C}H$), 46.7 ($\underline{C}H$), 52.7 ($\underline{C}H_2CH{=}CH_2$), 53.4 ($O\underline{C}H_3$), 54.7 ($\underline{C}H$), 92.6 ($\underline{C}HNO_2$), 119.1, 127.4, 128.8, 129.3, 132.1, 135.3

($\underline{C}$-Ar, $CH_2\underline{C}H=\underline{C}H_2$), 167.9, 171.0 ($\underline{C}$=O); HRMS–ES+ (*m*/*z*): $[M + Na]^+$ calculated for $C_{17}H_{20}N_2NaO_5$, 355.1264; found, 355.1269.

2.2.6. Synthesis and characterization of 1e

(±)-(5*R*,8*R*,9*S*,10*R*)-2,8-Dimethyl-9-nitro-10-phenyl-7-(prop-2-en-1-yl)-2,7-diazaspiro[4.5]decane-1,6-dione (**1e**)

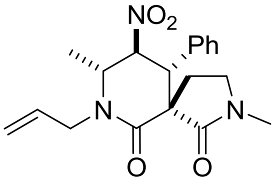

(±)-1e

According to the general procedure B (for 0.49 mmol, 0.150 g of **6a** used 0.98 mmol, 74 µL, of allylamine (**4a**); 0.98 mmol, 55 µL, of acetaldehyde (**3b**) and 3.8 mL of MeOH; under reflux; 24 h). **1e** (0.117 g, 67%) was obtained after column chromatography (Et_2O) as a colourless solid.
Mp 118–120 °C; IR (film) 2926 (C-H), 1685 (C=O), 1647 (C=O), 1553, 1338 (NO_2); ^{1}H NMR (500 MHz, $CDCl_3$) δ 1.59 (d, 3H, *J* = 6.0 Hz, $C\underline{H_3}CH$), 1.82–1.87 (m, 1H, $C\underline{H_A}H_BCH_2N$), 2.11–2.16 (m, 1H, $CH_2C\underline{H_A}H_BN$), 2.70 (s, 3H, $C\underline{H_3}N$), 3.01–3.07 (m, 1H, $CH_A\underline{H_B}CH_2N$), 3.17–3.21 (m, 1H, $CH_2H_A\underline{H_B}N$), 3.61 (d, 1H, *J* = 12.6 Hz, $C\underline{H}Ph$), 3.96 (dd, 1H, *J* = 15.8 Hz, *J* = 6.6 Hz, $C\underline{H_A}CH_BCH=CH_2$), 4.02–4.07 (m, 1H, $C\underline{H}CH_3$), 4.39 (dd, 1H, *J* = 15.8 Hz, *J* = 4.1 Hz, $CH_AC\underline{H_B}CH=CH_2$), 5.25–5.31 (m, 2H, $CH=C\underline{H_2}$), 5.80–5.88 (m, 1H, $C\underline{H}=CH_2$), 6.16 (dd, 1H, *J* = 12.6, 8.5 Hz, $C\underline{H}NO_2$), 7.21–7.39 (m, 5H, $\underline{H}$-Ar); ^{13}C NMR (125 MHz, $CDCl_3$) δ 19.5 ($\underline{C}H_3CH$), 27.1 ($\underline{C}H_2CH_2N$), 30.0 ($\underline{C}H_3N$), 47.2 ($CH_2\underline{C}H_2N$), 47.4 ($\underline{C}H_2CH=CH_2$), 49.8 ($\underline{C}HPh$), 55.5 ($CH_3\underline{C}H$), 55.9 (C_{quat}), 89.2 ($\underline{C}HNO_2$), 118.0, 128.8, 128.9, 132.3, 133.8 ($\underline{C}$-Ar, $CH_2\underline{C}H=\underline{C}H_2$), 167.9, 171.0 ($\underline{C}$=O); HRMS–ES+ (*m*/*z*): $[M + Na]^+$ calculated for $C_{19}H_{23}N_3NaO_4$, 380.1581; found, 380.1582.

2.2.7. Synthesis and characterization of 1f

(±)-(5*R*,8*R*,9*S*,10*R*)-7-Allyl-8-(4-methoxyphenyl)-2-methyl-9-nitro-10-phenyl-2,7-diazaspiro[4.5]decane-1,6-dione (**1f**)

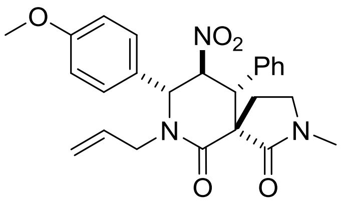

(±)-1f

According to the general procedure B (for 0.98 mmol, 0.300 g of **6a** used 1.96 mmol, 147 µL of allylamine (**4a**); 1.96 mmol, 0.267 g of anisaldehyde (**3c**) and 7.2 mL of MeOH; under reflux; 20 d). **1f** (0.221 g, 50%) was obtained after filtration of the solid precipitation from suspension and washing of the organics with MeOH (3 mL), as a colourless solid.
Mp 180–181 °C; IR (film) 2932 (C-H), 1677 (C=O), 1648 (C=O), 1554 (NO_2); ^{1}H NMR (500 MHz, $CDCl_3$) δ 1.91–1.96 (m, 1H, $C\underline{H_A}H_BCH_2N$), 2.08–2.13 (m, 1H, $CH_2C\underline{H_A}H_BN$), 2.72 (s, 3H, $C\underline{H_3}N$), 3.10–3.22 (m, 3H, $CH_A\underline{H_B}CH_2N$, $CH_2CH_A\underline{H_B}N$, $C\underline{H_A}H_BCH=CH_2$), 3.70 (d, 1H, *J* = 12.3 Hz, $C\underline{H}Ph$), 3.82 (s, 3H, $C\underline{H_3}O$), 4.56 (dd, 1H, *J* = 15.1, 3.8 Hz, $CH_A\underline{H_B}CH=CH_2$), 4.93 (d, 1H, *J* = 9.5 Hz,

N$C\underline{H}CHNO_2$), 5.00 ("d", 1H, *J* = 17.0 Hz, $CH_2CH{=}C\underline{H_A}H_B$), 5.19 ("d", 1H, *J* = 10.1 Hz, $CH_2CH{=}CH_A\underline{H_B}$), 5.68–5.76 (m, 1H, $CH_2CH{=}C\underline{H_2}$), 6.50 (dd, 1H, *J* = 12.3, 9.5 Hz, $C\underline{H}NO_2$), 6.90–7.41 (m, 9H, $\underline{H}$-Ar); ^{13}C NMR (125 MHz, $CDCl_3$) δ 27.6 ($\underline{C}H_2CH_2N$), 30.0 ($\underline{C}H_3N$), 47.1 ($CH_2\underline{C}H_2N$), 47.2 ($\underline{C}H_2CH{=}CH_2$), 50.7 ($\underline{C}HPh$), 55.2 ($\underline{C}H_3O$), 56.3 ($C_{quat}$), 63.7 ($N\underline{C}HCHNO_2$), 89.4 ($\underline{C}HNO_2$), 114.7, 118.6, 126.0, 128.1, 128.7, 128.9, 129.4, 131.6, 133.4, 160.2 ($\underline{C}$-Ar, $CH_2\underline{C}H{=}\underline{C}H_2$), 168.0, 171.2 ($\underline{C}{=}O$); HRMS–ES+ (*m*/*z*): $[M + H]^+$ calculated $C_{25}H_{28}N_3O_5$, 450.2023; found, 450.2030; Anal. calcd for $C_{25}H_{27}N_3O_5$: C, 66.80; H, 6.05; N, 9.35; found: C, 66.71; H, 6.08; N, 9.33%.

2.2.8. Synthesis and characterization of 1g

(±)-(5*R*,8*S*,9*S*,10*R*)-2-Methyl-9-nitro-1,6-dioxo-10-phenyl-7-(prop-2-en-1-yl)-2,7-diazaspiro[4.5]decane-8-carboxylic acid (**1g**)

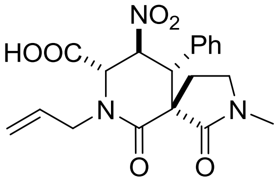

(±)-1g

Michael adduct **6a** (0.65 mmol, 0.200 g) was suspended in water (4.8 mL), glyoxylic acid (**3d**, 1.3 mmol, 0.120 g) and allylamine (**4a**, 2.6 mmol, 195 μL) were added and the mixture was stirred at 70 °C. After 3 h, the solution was cooled to rt and the pH was adjusted to ~2. The resulting suspension was stirred for 0.5 h at rt, and the insoluble precipitation was filtered off and washed with water, Et_2O (2 × 10 mL) to yield **1g** (0.187 g, 74%) as a colourless solid.

Mp 203–205 °C (CH_3CN); IR (film) 2923 (C-H), 1678 (C=O), 1665 (C=O), 1649 (C=O), 1557, 1357 (NO_2); ^{1}H NMR (500 MHz, *d*$_6$-DMSO) δ 1.67–1.73 (m, 1H, $C\underline{H_A}H_BCH_2N$), 2.06–2.11 (m, 1H, $CH_A\underline{H_B}CH_2N$), 2.60 (s, 3H, $C\underline{H_3}N$), 2.74–2.81 (m, 1H, $CH_2C\underline{H_A}H_BN$), 3.15–3.19 (m, 1H, $CH_2H_A\underline{H_B}N$), 3.58 (dd, 1H, *J* = 15.6, 7.1 Hz, $C\underline{H_A}CH_BCH{=}CH_2$), 3.85 (d, 1H, *J* = 12.3 Hz, $C\underline{H}Ph$), 4.69 (dd, 1H, *J* = 15.6, 4.6 Hz, $CH_AC\underline{H_B}CH{=}CH_2$), 4.79 (d, 1H, *J* = 5.7 Hz, $NC\underline{H}COOH$), 5.23–5.33 (m, 2H, $CH_2CH{=}C\underline{H_2}$), 5.80–5.88 (m, 1H, $C\underline{H}{=}CH_2$), 6.23 (dd, 1H, *J* = 12.3, 5.7 Hz, $C\underline{H}NO_2$), 7.25–7.30 (m, 2H, $\underline{H}$-Ar), 7.36–7.41 (m, 3H, $\underline{H}$-Ar), 13.73 (bs, 1H, $COO\underline{H}$); ^{13}C NMR (125 MHz, *d*$_6$-DMSO) δ 26.3 ($\underline{C}H_2CH_2N$), 29.6 ($\underline{C}H_3N$), 46.5 ($\underline{C}H_2N$), 48.9 ($\underline{C}H_2N$), 49.5 ($\underline{C}HPh$), 55.3 (C_{quat}), 60.4 ($\underline{C}HCOOH$), 87.0 ($\underline{C}HNO_2$), 118.1, 128.8, 128.9, 132.4, 134.3 ($\underline{C}$-Ar, $CH_2\underline{C}H{=}\underline{C}H_2$), 168.0, 169.4, 170.0 ($\underline{C}{=}O$); HRMS–ES+ (*m*/*z*): $[M + H]^+$ calculated for $C_{19}H_{22}N_3O_6$, 388.1503; found, 388.1509; Anal. calcd for $C_{19}H_{21}N_3O_6$: C, 58.91; H, 5.46; N, 10.85; found: C, 58.95; H, 5.52; N, 10.85%.

2.2.9. Synthesis and characterization of 1h

(±)-(5*R*,9*S*,10*R*)-7-Benzyl-2-methyl-9-nitro-10-phenyl-2,7-diazaspiro[4.5]decane-1,6-dione (**1h**)

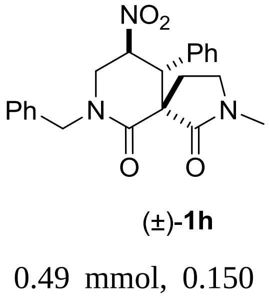

(±)-**1h**

According to the general procedure B (for 0.49 mmol, 0.150 g of **6a** used 0.98 mmol, 107 μL of benzylamine (**4b**); 0.98 mmol, 74 μL of formaldehyde (**3a**, 37% solution in water); 0.05 mmol, 0.014 g

of sodium dodecylsulfate; 3.5 mL of MeOH and 0.5 mL of water; at 70 °C; 24 h). **1h** (0.156 g, 81%) was obtained after flash column chromatography (Et_2O) as a colourless solid.
Mp 170–171 °C; IR (film) 2925 (C-H), 1682 (C=O), 1651 (C=O), 1553, 1352 (NO_2); ^{1}H NMR (500 MHz, $CDCl_3$) δ 1.92–1.97 (m, 1H, $C\underline{H}_AH_BCH_2N$), 2.12–2.17 (m, 1H, $CH_A\underline{H}_BCH_2N$), 2.67 (s, 3H, $C\underline{H}_3N$), 3.07–3.12 (m, 1H, $CH_2C\underline{H}_AH_BN$), 3.17–3.22 (m, 1H, $CH_2H_A\underline{H}_BN$), 3.67 (d, 1H, *J* = 12.0 Hz, $C\underline{H}Ph$), 3.75 (dd, 1H, *J* = 12.0 Hz, *J* = 9.5 Hz, $C\underline{H}_AH_BCHNO_2$), 4.05 (dd, 1H, *J* = 12.0, 6.6 Hz, $CH_A\underline{H}_BCHNO_2$), 4.55 (d, 1H, *J* = 14.8 Hz, $NC\underline{H}_AH_BPh$), 4.92 (d, 1H, *J* = 14.8 Hz, $NCH_A\underline{H}_BPh$), 6.33 (ddd, 1H, *J* = 12.0 Hz, *J* = 9.5, 6.6 Hz, $C\underline{H}NO_2$), 7.28–7.41 (m, 10H, $\underline{H}$-Ar); ^{13}C NMR (125 MHz, $CDCl_3$) δ 27.3 ($\underline{C}H_2CH_2N$), 29.9 ($\underline{C}H_3N$), 47.1 ($CH_2\underline{C}H_2N$), 49.0 ($C\underline{H_2}CHNO_2$), 51.0 ($N\underline{C}H_2Ph$), 51.2 ($CH\underline{C}HPh$), 56.4 (C_{quat}), 81.2 ($\underline{C}HNO_2$), 127.8, 127.8, 128.7, 128.8, 128.8, 128.9, 133.8, 135.4 ($\underline{C}$-Ar), 168.3, 171.2 ($\underline{C}$=O); HRMS–ES+ (*m*/*z*): $[M + Na]^+$ calculated for $C_{22}H_{23}N_3NaO_4$, 416.1581; found, 416.1577.

2.2.10. Synthesis and characterization of 1i

(±)-(5*R*,9*S*,10*R*)-7-Butyl-10-(furan-3-yl)-2-methyl-9-nitro-2,7-diazaspiro[4.5]decane-1,6-dione (**1i**)

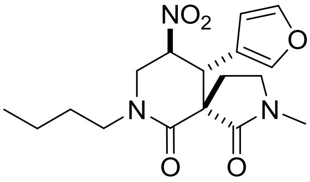

(±)-**1i**

According to general procedure B (for 5.40 mmol, 1.600 g of **6e** used 10.80 mmol, 0.790 g, 1.072 mL of butylamine (**4c**); 10.80 mmol, 811 µL of formaldehyde (**3a**, 37% solution in water) and 38 mL of MeOH; under reflux; 3 h) **1i** (1.541 g, 82%) was obtained after flash column chromatography (Et_2O) and trituration (Et_2O) as a colourless solid.
Mp 137 °C (Et_2O); IR (film) 2958, 2933, 2874 (C-H), 1683 (C=O), 1647 (C=O), 1555, 1348 (NO_2); ^{1}H NMR (500 MHz, $CDCl_3$) δ 0.94 (t, 3H, *J* = 7.3 Hz, $C\underline{H_3}CH_2CH_2$), 1.34 (sxt, 2H, *J* = 7.4 Hz, $CH_3C\underline{H_2}CH_2$), 1.51–1.62 (m, 2H, $CH_3CH_2C\underline{H_2}$), 1.87–1.93 (m, 1H, $C\underline{H}_AH_BCH_2NCH_3$), 2.75 (s, 3H, $C\underline{H_3}N$), 2.78–2.87 (m, 2H, $CH_A\underline{H}_BC\underline{H}_AH_BNCH_3$), 3.38–3.47 (m, 3H, $CH_2CH_A\underline{H}_BN$, $CH_2C\underline{H_2}N$), 3.57 (d, 1H, *J* = 11.7 Hz, $CHC\underline{H}Ar$), 3.79 (dd, 1H, *J* = 12.1, 9.0 Hz, $C\underline{H}_AH_BCHNO_2$), 4.07 (dd, 1H, *J* = 12.1, 6.5 Hz, $CH_A\underline{H}_BCHNO_2$), 6.18 (ddd, 1H, *J* = 11.6, 9.0, 6.5 Hz, $C\underline{H}NO_2$), 6.36 (s, 1H, $\underline{H}$-Ar), 7.35–7.37 (m, 2H, $\underline{H}$-Ar); ^{13}C NMR (125 MHz, $CDCl_3$) δ 13.8 ($\underline{C}H_3CH_2$), 20.0 ($CH_3\underline{C}H_2$), 27.6 ($\underline{C}H_2CH_2NCH_3$), 28.8 ($CH_3CH_2\underline{C}H_2$), 30.1 ($\underline{C}H_3N$), 42.4 ($CH\underline{C}HAr$), 47.7, 47.9 ($CH_2\underline{C}H_2NCH_3$, $CH_2\underline{C}H_2N$), 49.3 ($C\underline{H_2}CHNO_2$), 55.6 (C_{quat}), 81.9 ($\underline{C}HNO_2$), 109.5, 118.8, 141.7, 143.8 ($\underline{C}$-Ar), 167.8 ($CH_2N\underline{C}$=O), 171.4 ($CH_3N\underline{C}$=O); HRMS–ES+ (*m*/*z*): $[M + Na]^+$ calculated for $C_{17}H_{23}N_3NaO_5$, 372.1530; found, 372.1521.

2.2.11. Synthesis and characterization of 1j

(±)-(5*R*,9*S*,10*R*)-10-(3-Furyl)-7-(hept-5-yn-1-yl)-2-methyl-9-nitro-2,7-diazaspiro[4.5]decane-1,6-dione (**1j**)

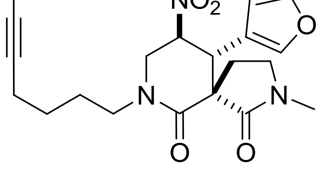

(±)-**1j**

According to general procedure B (for 6.60 mmol, 1.950 g of **6e** used 13.2 mmol, 1.468 g of amine **4d**; 13.2 mmol, 988 µL of formaldehyde (**3a**, 37% solution in water) and 48.0 mL of MeOH; under reflux; 4

h) **1j** (1.920 g, 75%) was obtained after flash column chromatography (Et_2O → Et_2O/MeOH 3:1) as a pale-yellow oil.
IR (film) 2923, 2865 (C-H), 1684 (C=O), 1648 (C=O), 1555, 1349 (NO_2); ^1H NMR (400 MHz, $CDCl_3$) δ 1.43–1.50 (m, 2H, $CH_2C\underline{H_2}CH_2CH_2N$), 1.64–1.73 (m, 2H, $CH_2CH_2C\underline{H_2}CH_2N$), 1.75 (t, 3H, *J* = 2.7 Hz, $C{\equiv}CC\underline{H_3}$), 1.85–1.92 (m, 1H, $C\underline{H_A}H_BCH_2NCH_3$), 2.13–2.19 (m, 2H, $C\underline{H_2}C{\equiv}C$), 2.72–2.85 (m, 5H, $NC\underline{H_3}$, $CH_A\underline{H_B}C\underline{H_A}H_BNCH_3$), 3.37–3.50 (m, 3H, $CH_2CH_2C\underline{H_2}N$, $CH_2CH_A\underline{H_B}NCH_3$), 3.55 (d, 1H, *J* = 11.6 Hz, $ArC\underline{H}CH$), 3.79 (dd, 1H, *J* = 12.1, 9.1 Hz, $C\underline{H_A}H_BCHNO_2$), 4.04 (dd, 1H, *J* = 12.1, 6.7 Hz, $CH_A\underline{H_B}CHNO_2$), 6.16 (ddd, 1H, *J* = 11.6, 9.1, 6.3 Hz, $C\underline{H}NO_2$), 6.34 (d, 1H, *J* = 1.5 Hz, $\underline{H}$-Ar), 7.34–7.35 (m, 2H, $\underline{H}$-Ar); ^{13}C NMR (100 MHz, $CDCl_3$) δ 3.4 ($\underline{C}H_3C{\equiv}C$), 18.3 ($CH_3C{\equiv}C\underline{C}H_2$), 25.8, 25.9 ($CH_2\underline{C}H_2\underline{C}H_2CH_2N$), 27.6 ($C\underline{H_2}CH_2NCH_3$), 30.0 ($\underline{C}H_3N$), 42.4 ($Ar\underline{C}HCH$), 47.5, 47.6 ($CH_2\underline{C}H_2N$, $CH_2\underline{C}H_2N$), 49.2 ($\underline{C}H_2CHNO_2$), 55.6 (C_{quat}), 76.0 ($CH_3\underline{C}{\equiv}C$), 78.4 ($CH_3C{\equiv}\underline{C}$), 81.8 ($\underline{C}HNO_2$), 109.4, 118.7, 141.7, 143.7 ($\underline{C}$-Ar), 167.8, ($CH_2N\underline{C}{=}O$), 171.3 ($CH_3N\underline{C}{=}O$); HRMS–ES+ (*m*/*z*): $[M + Na]^+$ calculated for $C_{20}H_{25}N_3NaO_5$, 410.1686; found, 410.1675.

2.2.12. Synthesis and characterization of 2a

(±)-(1*S*,2*R*,3*R*,11b*R*)-1'-Methyl-1-nitro-2-phenyl-1,6,7,11b-tetrahydro-2*H*,2'*H*-spiro[pyrido[2,1-*a*]isoquinoline-3,3'-pyrrolidine]-2',4-dione (**2a**)

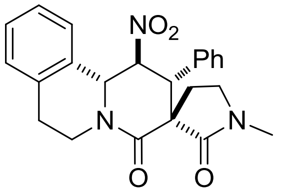

(±)-2a

A mixture of **6a** (0.65 mmol, 200 mg) and imine **5a** (1.31 mmol, 0.171 mg) in water (5.0 mL) was stirred at 70 °C. After 48 h the suspension was cooled to rt, and the insoluble solid was filtered off, washed successively with water (2 mL) and Et_2O (5 mL), and dried to yield **2a** (0.216 g, 76%) as a colourless solid. Mp 215–216 °C; IR (film) 2933 (C-H), 1695 (C=O), 1643 (C=O), 1556, 1361 (NO_2); ^1H NMR (500 MHz, $CDCl_3$) δ 1.77–1.83 (m, 1H, $C\underline{H_A}H_BCH_2N$), 2.05–2.10 (m, 1H, $CH_2C\underline{H_A}H_BN$), 2.57 (s, 3H, $C\underline{H_3}N$), 2.78 (dt, 1H, *J* = 15.8 Hz, *J* = 4.7 Hz, $C\underline{H_A}H_BCH_2N$), 2.92–2.98 (m, 1H, $CH_A\underline{H_B}CH_2N$), 3.02–3.14 (m, 2H, $CH_A\underline{H_B}CH_2N$, $CH_2CH_A\underline{H_B}N$), 3.20–3.26 (m, 1H, $CH_2C\underline{H_A}H_BN$), 3.73 (d, 1H, *J* = 12.0 Hz, $C\underline{H}Ph$), 4.41 (dt, 1H, *J* = 12.3, 5.0 Hz, $CH_2CH_A\underline{H_B}N$), 5.35 (d, 1H, *J* = 8.5 Hz, $NC\underline{H}CH$), 6.48 (dd, 1H, *J* = 12.0, 8.5 Hz, $C\underline{H}NO_2$), 7.09–7.36 (m, 9H, $\underline{H}$-Ar); ^{13}C NMR (125 MHz, $CDCl_3$) δ 27.2 ($\underline{C}H_2CH_2N$), 28.3 ($\underline{C}H_2CH_2N$), 29.9 ($\underline{C}H_3N$), 42.9 ($\underline{C}H_2N$), 47.1 ($\underline{C}H_2N$), 51.2 ($\underline{C}HPh$), 55.6 (C_{quat}), 58.5 ($N\underline{C}HCH$), 87.4 ($\underline{C}HNO_2$), 123.7, 127.2, 128.0, 128.8, 128.8, 129.0, 131.4, 133.3, 134.2, 135.7 ($\underline{C}$-Ar), 167.7, 171.0 ($\underline{C}{=}O$); HRMS–ES+ (*m*/*z*): $[M + Na]^+$ calculated for $C_{23}H_{23}N_3NaO_4$, 428.1581; found, 428.1586.

2.2.13. Synthesis and characterization of 2a and 2a''

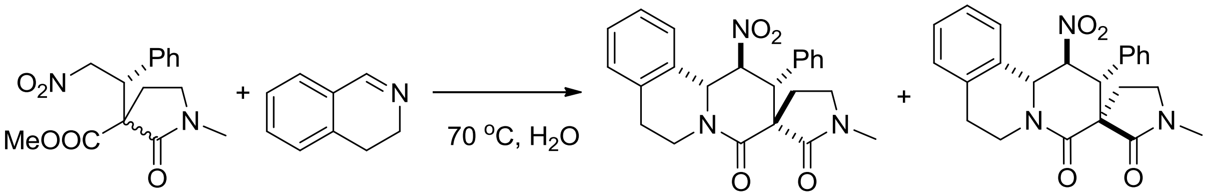

(±)-**6a, 6a"** **5a** (±)-**2a** (±)-**2a"**

An epimeric mixture of **6a**, **6a''** (1.63 mmol, 500 mg of the epimeric mixture, dr **6a**:**6a''** 38:62) and imine **5a** (3.27 mmol, 0.428 g) in water (13.0 mL) was stirred at 70 °C. After 48 h the suspension was cooled to rt, and the insoluble solid was filtered off, washed successively with water (4 mL) and Et_2O (5

mL), and dried to yield an epimeric mixture of **2a** and **2a''** (0.510 g, 72%, dr 38:62) as a colourless solid. The mixture of diastereomers was separated by flash column chromatography (EtOAc) to yield **2a** (0.197 g, 28%) and **2a''** (0.301 g, 42%).

(±)-(1*S*,2*R*,3*S*,11b*R*)-1'-Methyl-1-nitro-2-phenyl-1,6,7,11b-tetrahydro-2*H*,2'*H*-spiro[pyrido[2,1-*a*]isoquinoline-3,3'-pyrrolidine]-2',4-dione (**2a''**)

Mp 206–207 °C; IR (film) 2926 (C-H), 1690 (C=O), 1643 (C=O), 1556, 1361 (NO_2); ^{1}H NMR (500 MHz, $CDCl_3$) δ 1.91–1.98 (m, 1H, $CH_AH_BCH_2N$), 2.17–2.22 (m, 1H, $CH_AH_BCH_2N$), 2.34–2.39 (m, 1H, $CH_2CH_AH_BN$) 2.65 (s, 3H, CH_3N), 2.76–2.81 (m, 1H, $CH_AH_BCH_2N$), 2.91–2.97 (m, 1H, $CH_AH_BCH_2N$), 3.04–3.09 (m, 1H, $CH_2CH_AH_BN$), 3.24 ("dt", 1H, *J* = 8.5, 5.7 Hz, $CH_2CH_AH_BN$), 4.49 (d, 1H, *J* = 11.7 Hz, CHCHPh), 4.55 (dt, 1H, *J* = 8.2, 4.4 Hz, $CH_2CH_AH_BN$), 5.13 (dd, 1H, *J* = 11.7, 9.1 Hz, $CHNO_2$), 5.72 (d, 1H, *J* = 9.1 Hz, NCHCH), 7.12–7.27 (m, 9H, H-Ar); ^{13}C NMR (125 MHz, $CDCl_3$) δ 26.0 (CH_2CH_2N), 27.9 (CH_2CH_2N), 29.2 (CH_3N), 40.7, 46.1 (CH_2N), 47.3 (CHPh), 55.6 (C_{quat}), 57.0 (NCHCH), 88.7 ($CHNO_2$), 124.1, 126.3, 127.4, 127.6, 127.8, 128.1, 131.4, 132.8, 135.2 (C-Ar), 167.5, 170.7 (C=O); HRMS–ES+ (*m*/*z*): $[M + Na]^+$ calculated for $C_{23}H_{23}N_3NaO_4$, 428.1581; found, 428.1572; Anal. calcd for $C_{23}H_{23}N_3O_4$: C, 68.13; H, 5.72; N, 10.36; found: C, 67.93; H, 5.72; N, 10.28%.

2.2.14. Synthesis and characterization of 2m and 2m'

According to general procedure B (for 0.50 mmol, 0.141 g of **6d** used 1.00 mmol, 0.195 g of imine **4d**; 3.0 mL of MeOH; 3.0 mL of water under reflux; 24 h) **2m** (0.011 g, 5%) and **2m'** (0.155 g, 70%) were obtained after flash column chromatography (PE/EtOAc 1:2) as off-white solids [16].

Methyl (1*S*,2*S*,3*R*,11b*R*)-9,10-dimethoxy-1-nitro-4-oxo-2-phenyl-1,3,4,6,7,11b-hexahydro-2*H*-pyrido[2,1-*a*]isoquinoline-3-carboxylate (**2m**)

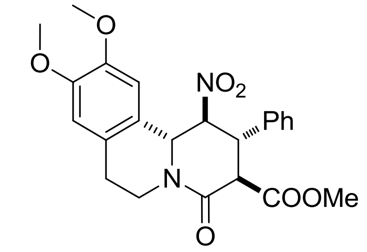

(±)-**2m**

^{1}H NMR (400 MHz, $CDCl_3$) δ 2.74 (dt, 1H, *J* = 15.3, 3.1 Hz, $NCH_2CH_AH_B$), 2.91–3.08 (m, 2H, $NCH_2CH_AH_B$, $NCH_AH_BCH_2$), 3.67 (s, 3H, $COOCH_3$), 3.77 (s, 3H, $ArOCH_3$), 3.79 (d, 1H, *J* = 10.1 Hz, $CHCOOCH_3$), 3.87 (s, 3H, $ArOCH_3$), 4.27 ("t" 1H, *J* = 10.5 Hz, CHCHPh), 4.73–4.78 (m, 1H, $NCH_AH_BCH_2$), 4.96 (dd, 1H, *J* = 11.1, 9.3 Hz, $CHNO_2$), 5.37 (d, 1H, *J* = 9.1 Hz, NCHCH), 6.56 (s, 1H, H-Ar), 6.67 (s, 1H, H-Ar), 7.19–7.21 (m, 2H, H-Ar), 7.31–7.37 (m, 3H, H-Ar); ^{13}C NMR (100 MHz, $CDCl_3$) δ 28.3 (CH_2CH_2N), 41.3 (CH_2CH_2N), 46.0 (CHCHPh), 53.0 ($COOCH_3$), 54.1 ($CHCOOCH_3$), 55.9, 55.9 (2 ×$ArOCH_3$), 58.2 (NCHCH), 92.8 ($CHNO_2$), 108.0, 111.7, 123.7, 127.4, 128.1, 128.9, 129.4, 135.8, 148.8, 148.1 (C-Ar), 164.0, 168.5 (C=O); HRMS–ES+ (*m*/*z*): $[M + Na]^+$ calculated for $C_{23}H_{24}N_2NaO_7$, 463.1476; found, 463.1479.

Methyl (1*R*,2*S*,3*R*,11b*R*)-9,10-dimethoxy-1-nitro-4-oxo-2-phenyl-1,3,4,6,7,11b-hexahydro-2*H*-pyrido[2,1-*a*]isoquinoline-3-carboxylate (**2m'**)

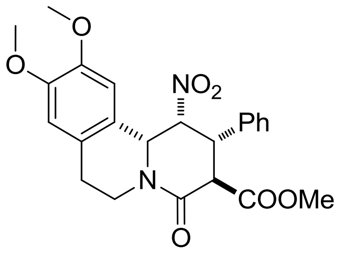

(±)-**2m'**

IR (film) 2952, 2838 (C-H), 1742 (C=O), 1643 (C=O), 1553, 1362 (NO_2); ^{1}H NMR (400 MHz, $CDCl_3$) δ 2.59–2.63 (m, 1H, $NCH_2C\underline{H}_AH_B$), 2.78–2.95 (m, 2H, $NCH_2CH_A\underline{H}_B$, $NC\underline{H}_AH_BCH_2$), 3.65 (s, 3H, $COOC\underline{H}_3$), 3.80 (s, 3H, $ArOC\underline{H}_3$), 3.84 (s, 3H, $ArOC\underline{H}_3$), 4.16 (dd, 1H, *J* = 12.6, 3.5 Hz, $C\underline{H}Ph$), 4.35 (d, 1H, *J* = 12.6 Hz, $C\underline{H}COOCH_3$), 5.08–5.12 (m, 1H, $NCH_A\underline{H}_BCH_2$), 5.36 (d, 1H, *J* = 4.0 Hz, NCH), 5.49 ("t", 1H, 4.0, 3.7 Hz, $C\underline{H}NO_2$), 6.60 (s, 1H, $\underline{H}$-Ar), 6.61 (s, 1H, $\underline{H}$-Ar), 7.18–7.22 (m, 2H, $\underline{H}$-Ph), 7.33–7.40 (m, 3H, $\underline{H}$-Ph); ^{13}C NMR (100 MHz, $CDCl_3$) δ 27.8 ($\underline{C}H_2CH_2N$), 39.7 ($CH_2\underline{C}H_2N$), 43.1 ($\underline{C}HPh$), 49.4 ($\underline{C}HCOOCH_3$), 52.8 ($COO\underline{C}H_3$), 55.7 ($ArO\underline{C}H_3$), 56.0 ($ArO\underline{C}H_3$), 58.4 ($\underline{C}HN$), 88.6 ($\underline{C}HNO_2$), 107.6, 111.8, 121.1, 127.0, 128.7, 128.9, 129.3, 134.9, 147.9, 148.5, 163.9 ($\underline{C}$-Ar), 169.9 (2 × $\underline{C}$=O); HRMS–ES+ (*m*/*z*): $[M + Na]^+$ calculated for $C_{23}H_{24}N_2NaO_7$, 463.1476; found, 463.1474.

2.3. General procedure C for the nitro-group removal

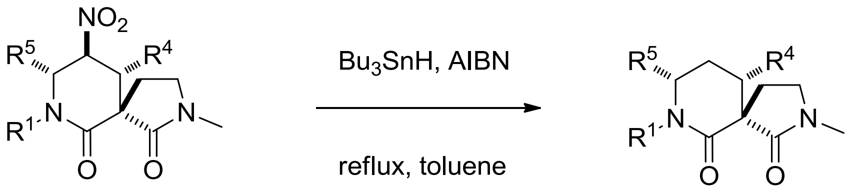

(±)-**1**,**2** (±)-**10**

A lactam **1** or **2** was suspended in toluene (c = 0.05 M) under an atmosphere of N_2. Bu_3SnH (5 equiv) and AIBN (0.2 equiv) were added, the mixture was degassed and filled with N_2. This operation was repeated three times. The mixture was stirred under reflux until all of the starting material had been consumed (TLC monitoring, typically 3–7 hours). The mixture was cooled to rt and concentrated in vacuo. The residue was purified by column chromatography to yield spirocycle **10**.

2.3.1. Synthesis and characterisation of 10a

(±)-(5*R*,10*R*)-7-Butyl-10-(furan-3-yl)-2-methyl-2,7-diazaspiro[4.5]decane-1,6-dione (**10a**)

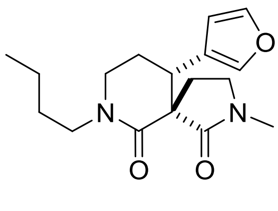

(±)-**10a**

Lactam **1i** (3.29 mmol, 1.150 g) was suspended in toluene (66 mL, c = 0.05 M) under an atmosphere of N_2. Bu_3SnH (5 equiv, 16.45 mmol, 4.788 g, 4.425 mL) and AIBN (0.2 equiv, 0.66 mmol, 0.108 g) were added, the suspension was degassed and filled with N_2. This operation was repeated three times. The mixture was stirred under reflux for 3 h before being cooled to rt and concentrated in vacuo. Petroleum ether (20 mL) was added, and, after 15 min at rt, the insoluble solid was filtered off and washed (petroleum ether, 15 mL). The filtration cake was purified by column chromatography (Et_2O) to yield spirocycle **10a** (0.531 g, 53%) as a colourless solid.

Mp 129–132 °C; IR (film) 2956, 2932, 2872 (C-H), 1682 (C=O), 1633 (C=O); ^{1}H NMR (500 MHz, $CDCl_3$) δ 0.92 (t, 3H, *J* = 7.3 Hz, $C\underline{H_3}CH_2CH_2$), 1.34 (sxt, 2H, *J* = 7.5 Hz, $CH_3C\underline{H_2}CH_2$), 1.50–1.63 (m, 2H, $CH_3CH_2C\underline{H_2}$), 1.70–1.74 (m, 1H, $C\underline{H_A}H_BCHAr$), 1.82–1.87 (m, 1H, $C\underline{H_A}H_BCH_2NCH_3$), 2.67–2.73 (m, 4H, $C\underline{H_3}N$, $CH_2C\underline{H_A}H_BNCH_3$), 2.81–2.86 (m, 1H, $CH_A\underline{H_B}CH_2NCH_3$), 2.90 (dd, 1H, *J* = 13.2, 2.8 Hz, $CH_2C\underline{H}Ar$), 3.14–3.23 (m, 1H, $CH_A\underline{H_B}CHAr$), 3.30–3.49 (m, 5H, $CH_2CH_A\underline{H_B}NCH_3$, $NC\underline{H_2}CH_2CHAr$, $CH_2CH_2C\underline{H_2}N$), 6.36 (s, 1H, $\underline{H}$-Ar), 7.32–7.33 (m, 2H, $\underline{H}$-Ar); ^{13}C NMR (125 MHz, $CDCl_3$) δ 13.8 ($\underline{C}H_3CH_2$), 20.1 ($CH_3\underline{C}H_2$), 25.5 ($\underline{C}H_2CHAr$), 28.9 ($\underline{C}H_2CH_2NCH_3$), 29.0 ($CH_3CH_2\underline{C}H_2$), 29.8 ($\underline{C}H_3N$), 39.9 ($CH_2\underline{C}HAr$), 47.4, 47.5, 47.9 ($CH_2\underline{C}H_2NCH_3$, $CH_2\underline{C}H_2N$, $CH_2\underline{C}H_2N$), 55.5 (C_{quat}), 110.1, 124.4, 139.7, 142.9 ($\underline{C}$-Ar), 169.5 ($CH_2N\underline{C}$=O), 171.4 ($CH_3N\underline{C}$=O); HRMS–ES+ (*m*/*z*): $[M + Na]^+$ calculated for $C_{17}H_{24}N_2NaO_3$, 327.1679; found, 327.1681.

2.3.2. Synthesis and characterization of 10b

(±)-(5*R*,10*R*)-10-(3-Furyl)-7-(hept-5-yn-1-yl)-2-methyl-2,7-diazaspiro[4.5]decane-1,6-dione (**10b**)

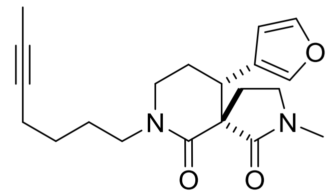

(±)-**10b**

According to general procedure C (for 4.90 mmol, 1.900 g of **1j** used 0.98 mmol, 0.160 g of AIBN; 24.50 mmol, 6.59 mL of Bu_3SnH and 98 mL of toluene; under reflux; 6h) **10b** (1.408 g, 84%) was obtained after column chromatography (Et_2O → EtOAc) as a pale yellow solid.

Mp 59–62 °C; IR (film) 2932, 2864 (C-H), 1682 (C=O), 1633 (C=O); ^{1}H NMR (500 MHz, $CDCl_3$) δ 1.46–1.52 (m, 2H, $CH_2C\underline{H_2}CH_2CH_2N$), 1.65–1.77 (m, 6H, $C{\equiv}CC\underline{H_3}$, $CH_2CH_2C\underline{H_2}CH_2N$, $C\underline{H_A}CH_BCHAr$), 1.82–1.87 (m, 1H, $C\underline{H_A}H_BCH_2NCH_3$), 2.14–2.18 (m, 2H, $C\underline{H_2}C{\equiv}C$), 2.67–2.72 (m, 4H, $NC\underline{H_3}$, $CH_2C\underline{H_A}H_BNCH_3$), 2.83 (ddd, 1H, *J* = 12.7, 9.4, 5.7 Hz, $CH_A\underline{H_B}CH_2NCH_3$), 2.91 (dd, 1H, *J* = 13.2, 2.8 Hz, $C\underline{H}Ar$), 3.14–3.23 (m, 1H, $CH_A\underline{H_B}CHAr$), 3.32 (dt, 1H, *J* = 9.1, 4.5 Hz, $CH_2CH_A\underline{H_B}NCH_3$), 3.37–3.49 (m, 4H, $NC\underline{H_2}CH_2CHAr$, $CH_2CH_2C\underline{H_2}N$), 6.36 (s, 1H, $\underline{H}$-Ar), 7.32–7.33 (m, 2H, $\underline{H}$-Ar); ^{13}C NMR (125 MHz, $CDCl_3$) δ 3.5 ($\underline{C}H_3C{\equiv}C$), 18.5 ($CH_3C{\equiv}C\underline{C}H_2$), 25.5 ($\underline{C}H_2CHAr$), 26.1, 26.2 ($CH_2\underline{C}H_2\underline{C}H_2CH_2N$), 28.9 ($C\underline{H_2}CH_2NCH_3$), 29.8 ($\underline{C}H_3N$), 39.8 ($CH_2\underline{C}HAr$), 47.4, 47.5, 47.6 ($CH_2\underline{C}H_2N$, $CH_2\underline{C}H_2N$, $CH_2\underline{C}H_2N$), 55.5 (C_{quat}), 75.7 ($CH_3\underline{C}{\equiv}C$), 78.8 ($CH_3C{\equiv}\underline{C}$), 110.1, 124.3, 139.7, 142.9, ($\underline{C}$-Ar), 169.6, ($CH_2N\underline{C}$=O), 172.5 ($CH_3N\underline{C}$=O); HRMS–ES+ (*m*/*z*): $[M + Na]^+$ calculated for $C_{20}H_{26}N_2NaO_3$, 365.1836; found, 365.1836.

2.3.3. Synthesis and characterization of 10c

(±)-(2*R*,3*R*,11b*S*)-1'-Methyl-2-phenyl-1,6,7,11b-tetrahydro-2H,2'H-spiro[pyrido[2,1-*a*]isoquinoline-3,3'-pyrrolidine]-2',4-dione (**10c**)

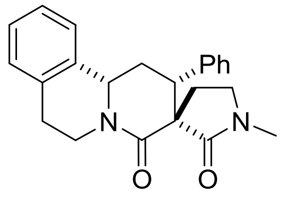

(±)-10c

A lactam **2a** (1.28 mmol, 520 mg) was suspended in toluene (26 mL, c = 0.05 M) under an atmosphere of N_2, then Bu_3SnH (5 equiv, 6.40 mmol, 1866 mg, 1.76 mL) and AIBN (0.2 equiv, 0.26 mmol, 42 mg) were added, and the suspension was degassed and filled with N_2. The operation was repeated three times. The mixture was stirred under reflux for 5 h. The mixture was cooled to rt and concentrated in vacuo. Petroleum ether (20 mL) was added, and the insoluble solid was filtered off and washed (petroleum ether, 2 × 5 mL). The filtration cake was purified by column chromatography (EtOAc) to yield spirocycle **10c** (0.328 g, 71%) as an off-white solid.

Mp 218–220 °C (dec., EtOAc); IR (film) 2930, 2873 (C-H), 1682 (C=O), 1632 (C=O); ^{1}H NMR (500 MHz, $CDCl_3$) δ 1.96–2.00 (m, 1H, $C\underline{H_A}H_BCH_2NCH_3$), 2.12–2.16 (m, 1H, $CH_2C\underline{H_A}H_BNCH_3$), 2.41–2.45 (m, 1H, $NCHC\underline{H_A}H_BCH$), 2.54 (s, 3H, $NC\underline{H_3}$), 2.78 (dt, 1H, *J* = 15.5, 3.3 Hz, $PhC\underline{H_A}H_BCH_2N$), 2.98–3.14 (m, 4H, $CH_A\underline{H_B}CH_A\underline{H_B}NCH_3$, $PhCH_A\underline{H_B}C\underline{H_A}H_BN$), 3.18 (dd, 1H, *J* = 13.6, 2.2 Hz, $CH_2C\underline{H}Ar$), 3.28–3.35 (m, 1H, $NCHCH_A\underline{H_B}CH$), 4.82–4.89 (m, 2H, $NC\underline{H}CH_2$, $PhCH_2CH_A\underline{H_B}N$), 7.16–7.21 (m, 4H, $\underline{H}$-Ph), 7.29–7.32 (m, 5H, $\underline{H}$-Ph); ^{13}C NMR (125 MHz, $CDCl_3$) δ 28.9, 29.0 ($\underline{C}H_2CH_2N$, $\underline{C}H_2CH_2N$), 29.6 ($\underline{C}H_3N$), 32.5 ($CH\underline{C}H_2CH$), 40.8 ($PhCH_2\underline{C}H_2N$), 46.9 ($CH_2\underline{C}H_2NCH_3$), 47.8 ($CH_2\underline{C}HPh$), 56.3 ($C_{quat}$), 56.8 ($N\underline{C}HCH_2$), 124.9, 126.4, 126.6, 127.6, 128.4, 128.5, 128.8, 135.0, 136.9, 140.1 ($\underline{C}$-Ar), 169.5, ($CH_2N\underline{C}$=O), 172.1 ($CH_3N\underline{C}$=O); HRMS–ES+ (*m*/*z*): $[M + Na]^+$ calculated for $C_{23}H_{24}N_2NaO_2$, 383.1730; found, 383.1725.

2.3.4. Synthesis and characterization of 10d

(±)-(2*R*,3*R*,12b*S*)-1'-methyl-2-phenyl-1,2,6,7,12,12b-hexahydro-2'*H*-spiro[indolo[2,3-*a*]quinolizine-3,3'-pyrrolidine]-2',4-dione (**10d**)

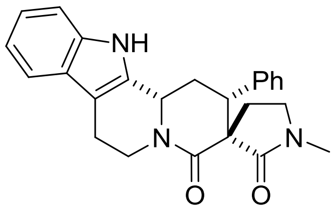

(±)-**10d**

Spirocycle **2c** was suspended in toluene (20 mL) under an atmosphere of N_2. Bu_3SnH (5.56 mmol, 1.471 mL) and AIBN (0.22 mmol, 0.037 g) were added and the suspension was degassed and filled with N_2. This operation was repeated three times. The suspension was stirred under reflux until all of the starting material had been consumed (4 h). The suspension was cooled to rt, and the solid precipitate was filtered off, washed with toluene (10 mL) and petroleum ether (2 × 20 mL), and dissolved in chloroform. The solution was concentrated in vacuo to yield spirocycle **10d** as a colourless solid (0.361 g, 81%).

Mp 287–290 °C; IR (film) 2911 (C-H), 1669 (C=O), 1613 (C=O); ^{1}H NMR (500 MHz, $CDCl_3$) δ 1.96–2.01 (m, 1H, $C\underline{H_A}H_BCH_2N$), 2.15–2.19 (m, 1H, $CH_2C\underline{H_A}H_BN$), 2.28–2.33 (m, 1H, $CHC\underline{H_A}CH_BCH$),

2.53 (s, 3H, $C\underline{H_3}N$), 2.73–2.80 (m, 1H, $C\underline{H_A}H_BCH_2N$), 2.88–3.05 (m, 3H, $CH_A\underline{H_B}CH_2N$, $CH_A\underline{H_B}CH_2N$, $CH_2C\underline{H_A}H_BN$), 3.09–3.15 (m, 2H, $C\underline{H}Ph$, $CH_2CH_A\underline{H_B}N$), 3.41 (m, 1H, $CHCH_A\underline{H_B}CH$), 4.91 (dd, 1H, J = 11.4, 4.4 Hz, $NC\underline{H}CH_2$), 5.13–5.21 (m, 1H, $CH_2CH_A\underline{H_B}N$), 7.09–7.17 (m, 2H, $\underline{H}$-Ar), 7.26–7.31 (m, 6H, $\underline{H}$-Ar), 7.49 (d, 1H, J = 7.9 Hz, $\underline{H}$-Ar), 7.89 (s, 1H, $N\underline{H}$); ^{13}C NMR (125 MHz, $CDCl_3$) δ 21.0 ($\underline{C}H_2CH_2N$), 28.9 ($\underline{C}H_2CH_2N$), 29.7 ($\underline{C}H_3N$), 31.0 ($CH\underline{C}H_2CH$), 41.2, 47.0 (2 × $CH_2\underline{C}H_2N$), 47.3 ($\underline{C}HPh$), 54.2 ($N\underline{C}HCH_2$), 56.7 (C_{quat}), 109.7, 110.9, 118.5, 119.7, 122.0, 126.9, 127.8, 128.5, 128.5, 132.7, 136.3, 139.7 ($\underline{C}$-Ar), 169.6, 171.9 ($\underline{C}$=O); HRMS–ES+ (*m*/*z*): $[M + H]^+$ calculated for $C_{25}H_{26}N_3O_2$, 400.2020; found, 400.2017.

2.4. General procedure D for the chemoselective reduction of 10

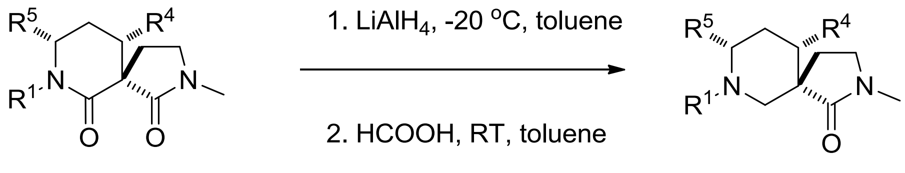

(±)-**10** (±)-**11**

$LiAlH_4$ (3 equiv, 1 M solution in THF) was added dropwise to a solution of **10** in toluene (c = 0.05 M) at –78 ºC. The mixture was warmed to –20 ºC and stirred for 1 h. The mixture was cooled to –78 ºC, formic acid (71 equiv) was added dropwise with care and the mixture was warmed to rt with stirring. After 24 h Rochelle salt (20% solution in water, 20 mL per 1 mmol of **10**) was added, the pH was adjusted to 10 over 10 min by addition of solid K_2CO_3 until two clear phases appeared. Et_2O was added (10 mL per mmol of **10**), the emulsion was extracted, the organic phase was separated and the water phase was further extracted (2 × 10 mL of CH_2Cl_2 per mmol of **10**). The combined organics were dried (Na_2SO_4) and concentrated in vacuo. The residue was purified by column chromatography (Et_2O → Et_2O/MeOH 90:10) to afford **11**.

2.4.1. Synthesis and characterization of 11a

(±)-(5*S*,10*R*)-7-Butyl-10-(furan-3-yl)-2-methyl-2,7-diazaspiro[4.5]decan-1-one (**11a**)

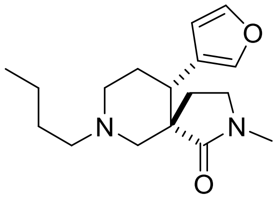

(±)-**11a**

According to general procedure D (for 0.66 mmol, 0.200 g of **10a** used 1.97 mmol, 1.97 mL of 1 M $LiAlH_4$ in THF; 49.93 mmol, 1.884 mL of formic acid and 12.8 mL of toluene) **11a** (0.155 g, 81%) was obtained as a pale yellow oil.

IR (film) 2956, 2928, 2873 (C-H), 1676 (C=O); ^1H NMR (400 MHz, $CDCl_3$) δ 0.90 (t, 3H, J = 7.3 Hz, $C\underline{H_3}CH_2CH_2$), 1.26–1.35 (m, 2H, $CH_3C\underline{H_2}CH_2$), 1.46–1.65 (m, 4H, $CH_3CH_2C\underline{H_2}$, $C\underline{H_A}H_BCH_2NCH_3$, $CH_2C\underline{H_A}H_BCHAr$), 1.89–2.06 (m, 3H, $NC\underline{H_A}H_BC_{quat}$, $C\underline{H_A}H_BCH_A\underline{H_B}CHAr$), 2.26–2.41 (m, 3H, $CH_2C\underline{H}Ar$, $CH_2CH_2C\underline{H_2}N$), 2.65–2.80 (m, 5H, $C\underline{H_3}N$, $CH_A\underline{H_B}C\underline{H_A}H_BNCH_3$), 3.03–3.13 (m, 3H, $NCH_A\underline{H_B}C_{quat}$, $CH_2CH_A\underline{H_B}NCH_3$, $CH_A\underline{H_B}CH_2CHAr$), 6.37 (s, 1H, $\underline{H}$-Ar), 7.30–7.31 (m, 2H, $\underline{H}$-Ar); ^{13}C NMR (100 MHz, $CDCl_3$) δ 14.1 ($\underline{C}H_3CH_2$), 20.8 ($CH_3\underline{C}H_2$), 28.9, 29.3, 29.7, 31.1 ($CH_3CH_2\underline{C}H_2$, $\underline{C}H_2CHAr$, $\underline{C}H_2CH_2NCH_3$, $\underline{C}H_3N$), 41.3 ($CH_2\underline{C}HAr$), 45.8 ($CH_2\underline{C}H_2NCH_3$), 46.5 ($C_{quat}$), 54.4 ($\underline{C}H_2CH_2CHAr$), 58.8 ($CH_2CH_2\underline{C}H_2N$), 61.9 ($N\underline{C}H_2$Cquat.), 110.8, 125.5, 139.8, 142.5 ($\underline{C}$-Ar), 175.4 ($\underline{C}$=O); HRMS–ES+ (*m*/*z*): $[M + H]^+$ calculated for $C_{17}H_{27}O_2N_2$, 291.2067; found, 291.2071.

2.4.2. Synthesis and characterization of 11b

(±)-(5*S*,10*R*)-10-(3-Furyl)-7-(hept-5-yn-1-yl)-2-methyl-2,7-diazaspiro[4.5]decan-1-one (**11b**)

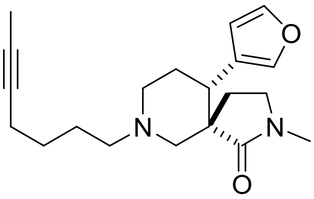

(±)-**11b**

According to general procedure D (for 0.59 mmol, 0.200 g of **10b** used 1.76 mmol, 1.76 mL of 1 M $LiAlH_4$ in THF; 42.50 mmol, 1.60 mL of formic acid and 11.8 mL of toluene) **11b** (0.148 g, 77%) was obtained after column chromatography (Et_2O → Et_2O/MeOH 90:10) as a pale yellow oil.
IR (film) 2936, 2920, 2866 (C-H), 1681 (C=O); ^{1}H NMR (400 MHz, $CDCl_3$) δ 1.44–1.65 (m, 6H, $C\underline{H_2}C\underline{H_2}CH_2N$, $C\underline{H_A}H_BCH_2NCH_3$, $CH_2C\underline{H_A}H_BCHAr$), 1.78 (t, 3H, *J* = 7.3 Hz, $C{\equiv}CC\underline{H_3}$), 1.87–1.99 (m, 3H, $NC\underline{H_A}H_BC_{quat}$, $C\underline{H_A}H_BCH_A\underline{H_B}CHAr$), 2.12–2.17 (m, 2H, $C{\equiv}CC\underline{H_2}$), 2.22–2.29 (m, 1H, $CH_2CH_2C\underline{H_A}H_BN$), 2.35–2.42 (m, 2H, $CH_2C\underline{H}Ar$, $CH_2CH_2CH_A\underline{H_B}N$), 2.65–2.81 (m, 5H, $C\underline{H_3}N$, $CH_A\underline{H_B}C\underline{H_A}H_BNCH_3$), 3.03–3.14 (m, 3H, $NCH_A\underline{H_B}C_{quat}$, $CH_2CH_A\underline{H_B}NCH_3$, $CH_A\underline{H_B}CH_2CHAr$), 6.37 (s, 1H, $\underline{H}$-Ar), 7.30–7.31 (m, 2H, $\underline{H}$-Ar); ^{13}C NMR (100 MHz, $CDCl_3$) δ 3.4 ($\underline{C}H_3C{\equiv}C$), 18.5 ($C{\equiv}C\underline{C}H_2$), 25.8, 26.9 ($\underline{C}H_2\underline{C}H_2CH_2N$), 29.1 ($\underline{C}H_2CH_2NCH_3$), 29.5 ($\underline{C}H_3N$), 30.8 ($\underline{C}H_2CHAr$), 41.1 ($CH_2\underline{C}HAr$), 45.7 ($CH_2\underline{C}H_2NCH_3$), 46.4 ($C_{quat}$), 54.2 ($\underline{C}H_2CH_2CHAr$), 58.3 ($CH_2CH_2\underline{C}H_2N$), 61.6 ($N\underline{C}H_2Cquat.$), 75.3 ($CH_3\underline{C}{\equiv}C$), 79.1 ($CH_3C{\equiv}\underline{C}$), 110.7, 125.3, 139.6, 142.3 ($\underline{C}$-Ar), 175.2 ($\underline{C}$=O); HRMS–ES+ (*m*/*z*): $[M + H]^+$ calculated for $C_{20}H_{29}N_2O_2$, 329.2224; found, 329.2215.

2.4.3. Synthesis and characterization of 11c

(±)-(2*R*,3*S*,11b*S*)-1'-Methyl-2-phenyl-1,6,7,11b-tetrahydro-2H,2'H-spiro[pyrido[2,1-*a*]isoquinoline-3,3'-pyrrolidin]-2'-one (**11c**)

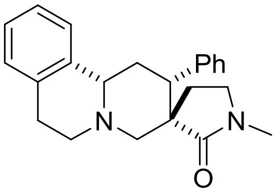

(±)-**11c**

According to general procedure D (for 0.28 mmol, 0.100 g of **10c** used 0.83 mmol, 0.83 mL of 1 M $LiAlH_4$ in THF; 20.16 mmol, 0.76 mL of formic acid and 5.6 mL of toluene) **11c** (0.068 g, 71%) was obtained as a pale yellow solid.
Mp 218–220 °C (dec., EtOAc); IR (film) 2930, 2873 (C-H), 1681 (C=O); ^{1}H NMR (400 MHz, $CDCl_3$) δ 1.67–1.74 (m, 1H, $C\underline{H_A}H_BCH_2NCH_3$), 1.96 (ddd, 1H, *J* = 12.8, 8.3, 4.2 Hz, $CH_A\underline{H_B}CH_2NCH_3$), 2.15–2.25 (m, 2H, $NCHC\underline{H_A}H_BCHPh$, $CH_2C\underline{H_A}H_BNCH_3$), 2.45–2.52 (m, 2H, $NC\underline{H_A}H_BC_{quat}$, $PhCH_2C\underline{H_A}H_BN$), 2.59 (s, 3H, $C\underline{H_3}N$), 2.68–2.88 (m, 3H, $CH_2C\underline{H}Ar$, $NCHCH_A\underline{H_B}CHPh$, $PhC\underline{H_A}H_BCH_2N$), 2.95 (dt, 1H, *J* = 9.2, 4.2 Hz, $CH_2CH_A\underline{H_B}NCH_3$), 3.03–3.08 (m, 1H, $PhCH_2CH_A\underline{H_B}N$), 3.15 (d, 1H, *J* = 11.9 Hz, $NCH_A\underline{H_B}Cquat$), 3.29–3.36 (m, 2H, $NC\underline{H}CH_2CHPh$, $PhCH_A\underline{H_B}CH_2N$), 7.07–7.20 (m, 4H, $\underline{H}$-Ph), 7.24–7.35 (m, 5H, $\underline{H}$-Ph); ^{13}C NMR (100 MHz, $CDCl_3$) δ 29.6, 29.6 ($\underline{C}H_3N$, $Ph\underline{C}H_2CH_2N$), 30.6 ($\underline{C}H_2CH_2NCH_3$), 34.2 ($NCH\underline{C}H_2CH$), 45.6 ($CH_2\underline{C}H_2NCH_3$), 46.9 ($C_{quat}$), 52.0 ($PhCH_2\underline{C}H_2N$), 52.8 ($NCHCH_2\underline{C}HAr$), 63.7 ($N\underline{C}HCH_2$), 65.0 ($N\underline{C}H_2C_{quat}$), 124.9, 125.5, 125.8, 127.1, 128.2, 128.8, 128.9, 135.0, 137.7, 141.6 ($\underline{C}$-Ph), 175.1 ($\underline{C}$=O); HRMS–ES+ (*m*/*z*): $[M + H]^+$ calculated for $C_{23}H_{27}N_2O$, 347.2118; found, 347.2215.

2.4.4. Synthesis and characterization of 12a

(±)-(2*R*,3*R*,12b*S*)-1'-Methyl-2-phenyl-1,2,6,7,12,12b-hexahydrospiro[indolo[2,3-*a*]quinolizine-3,3'-pyrrolidine] (**12a**)

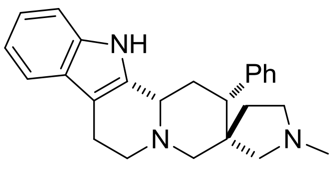

(±)-**12a**

A suspension of spirocycle **10d** (0.25 mmol, 0.100 g) in THF (4 mL) was cooled to –78 °C and DIBAL (2.5 mmol, 2.5 mL of 1 M solution in cyclohexane) was added dropwise at –78 °C under an atmosphere of N_2. The mixture was allowed to warm to rt and stirred for 24 h. The mixture was then cooled to 0 °C and 1 M HCl (1 mL) was carefully added. After 5 min of stirring the pH of the mixture was adjusted to 12 and the mixture was extracted with DCM (3 × 5 mL). The combined organic layers were dried (Na_2SO_4) and concentrated in vacuo. The crude product was purified by flash column chromatography (DCM/MeOH 80:20) to yield spirocycle **12a** (0.066 g, 71%) as a colourless solid.
Mp 246–248 °C; ^{1}H NMR (500 MHz, $CDCl_3$) δ 1.28–1.33 (m, 1H, $C\underline{H_A}H_BCH_2$), 1.38–1.43 (m, 1H, $CH_A\underline{H_B}CH_2$), 1.79–2.01 (m, 3H, $CHC\underline{H_2}CH$, $C\underline{H_A}H_BCH_2$), 2.07 (s, 3H, $C\underline{H_3}N$), 2.22 (d, 1H, *J* = 9.6 Hz, $CHNC\underline{H_A}H_BC_{quat}$), 2.32 (d, 1H, *J* = 11.4 Hz, $CH_3NC\underline{H_A}H_BC_{quat}$), 2.53–2.67 (m, 3H, $C\underline{H_A}H_BCH_2$, $C\underline{H_A}H_BCH_2$, $CH_A\underline{H_B}CH_2$), 2.72 (dd, 1H, *J* = 13.1 Hz, *J* = 3.0 Hz, $CHC\underline{H}Ph$), 2.88–3.05 (m, 3H, $CH_3NCH_A\underline{H_B}C_{quat}$, $CH_A\underline{H_B}CH_2$, $CH_A\underline{H_B}CH_2$), 3.19 (d, 1H, *J* = 9.6 Hz, $CHNCH_A\underline{H_B}C_{quat}$), 3.29 (broad d, 1H, *J* = 10.7 Hz, $NC\underline{H}CH_2$), 7.00–7.27 (m, 8H, $\underline{H}$-Ar), 7.42 (d, 1H, *J* = 7.6 Hz, $\underline{H}$-Ar), 7.64 (broad s, 1H, $N\underline{H}$); ^{13}C NMR (125 MHz, $CDCl_3$) δ 21.8 ($\underline{C}H_2CH_2N$), 34.0 ($CH\underline{C}H_2CH$), 34.7 ($\underline{C}H_2CH_2N$), 42.2 ($\underline{C}H_3N$), 46.8 ($C_{quat.}$), 50.5 ($\underline{C}HPh$), 52.7 ($CH_2\underline{C}H_2N$), 55.3 ($CH_2\underline{C}H_2N$), 59.9 ($CHN\underline{C}H_2C_{quat}$), 60.0 ($N\underline{C}HCH_2$), 67.8 ($CH_3N\underline{C}H_2C_{quat}$), 108.5, 110.7, 118.2, 119.4, 121.4, 126.8, 127.4, 128.0, 129.7, 134.6, 135.9, 141.5 ($\underline{C}$-Ar); HRMS–ES+ (*m*/*z*): $[M + H]^+$ calculated for $C_{25}H_{30}N_3$, 372.2445; found, 372.2433; Anal. calcd for $C_{25}H_{29}N_3$: C, 80.82; H, 7.87; N, 11.31; found: C, 79.75; H, 7.90; N, 11.14%.

2.4.5. Synthesis and characterization of 12b

(±)-(5*R*,10*S*)-7-Butyl-10-(furan-3-yl)-2-methyl-2,7-diazaspiro[4.5]decane (**12b**)

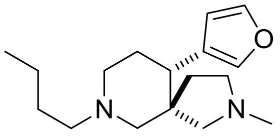

(±)-**12b**

A solution of spirocycle **10a** (0.33 mmol, 0.100 g) in THF (4 mL) was cooled to –78 °C and DIBAL (10 equiv, 3.30 mmol, 3.3 mL of 1 M solution in toluene) was added dropwise under an atmosphere of N_2. The mixture was allowed to warm to rt and stirred. After 4 d, MeOH (1 mL) was added carefully to the mixture and it was stirred for 1 h at rt. $Na_2SO_4.10\ H_2O$ (0.500 g) and Et_2O (8 mL) were added and the resulting suspension was vigorously stirred at rt. After 30 min the insoluble solid was filtered off, washed (Et_2O, 2 × 4 mL) and the filtrate was concentrated in vacuo. The residue was purified by column chromatography (Et_2O → Et_2O/MeOH 75:25) affording **12b** (0.068 g, 75%) as a pale-yellow oil.
IR (film) 2956, 2933 (C-H); ^{1}H NMR (400 MHz, $CDCl_3$) δ 0.91 (t, 3H, *J* = 7.3 Hz, $C\underline{H_3}CH_2CH_2$), 1.32 (sxt, 2H, *J* = 7.3 Hz, $CH_3C\underline{H_2}CH_2$), 1.41–1.53 (m, 3H, $CH_3CH_2C\underline{H_2}$, $C\underline{H_A}H_BCH_2NCH_3$), 1.65–1.70 (m, 2H, $CH_2C\underline{H_2}CHAr$), 1.73–1.85 (m, 3H, $C_{quat}C\underline{H_A}H_BN$, $CH_A\underline{H_B}C\underline{H_A}H_BNCH_3$), 1.95–2.04 (m, 1H,

$C\underline{H_A}H_BCH_2CHAr$), 2.21–2.23 (m, 4H, $C\underline{H_3}N$, $NC\underline{H_A}H_BC_{quat}$), 2.26–2.43 (m, 3H, $CH_2CH_2C\underline{H_2}N$, $CH_2C\underline{H}Ar$), 2.59–2.64 (m, 1H, $CH_2CH_A\underline{H_B}NCH_3$), 2.82–2.93 (m, 3H, $C_{quat}CH_A\underline{H_B}NCH_3$, $NCH_A\underline{H_B}C_{quat}$, $NCH_A\underline{H_B}CH_2CHAr$), 6.33 (s, 1H, $\underline{H}$-Ar), 7.28 (broad s, 1H, $\underline{H}$-Ar), 7.37 (d, 1H, J = 1.5 Hz, $\underline{H}$-Ar); ^{13}C NMR (100 MHz, $CDCl_3$) δ 14.0 ($\underline{C}H_3CH_2$), 20.7 ($CH_3\underline{C}H_2$), 29.2 ($CH_3CH_2\underline{C}H_2$) 30.3 ($CH_2\underline{C}H_2CHAr$), 36.6 ($\underline{C}H_2CH_2NCH_3$), 41.7 ($CH_2\underline{C}HAr$), 42.3 ($\underline{C}H_3N$), 45.9 ($C_{quat}$), 54.0 ($N\underline{C}H_2CH_2CHAr$), 55.6 ($CH_2\underline{C}H_2NCH_3$), 58.3 ($CH_2CH_2\underline{C}H_2N$), 61.2 ($N\underline{C}H_2C_{quat}$), 65.6 ($C_{quat}\underline{C}H_2NCH_3$), 111.5, 126.2, 139.7, 142.2 ($\underline{C}$-Ar); HRMS–ES+ (m/z): $[M + H]^+$ calculated for $C_{17}H_{29}N_2O$, 277.2274; found, 277.2273.

3. Elucidation of relative configuration:

The relative stereochemistry of compounds **1** and **2** was elucidated based on a combination of NMR techniques (coupling constant on 6-membered ring, n.O. effects and a comparison of ^1H NMR data) and single-crystal X-ray analysis.

3.1. nOes

3.1.1. nOes of compound 1c

(selected irradiation)

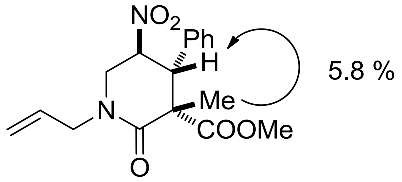

(±)-1c

3.2. Single-crystal X-ray crystallography
3.2.1. X-ray crystal structure of compound (±)-6e

Crystal data and structure refinement for 6e.

Identification code 6110

$C_{13}H_{16}N_2O_6$	$F(000) = 624$
$M_r = 296.28$	$D_x = 1.439$ Mg m^{-3}
Monoclinic, $P2_1/n$	Melting point: not measured K
Hall symbol: -P 2yn	Mo Kα radiation, λ = 0.71073 Å
a = 6.6527 (1) Å	Cell parameters from 3228 reflections
b = 14.3997 (2) Å	θ = 5–27°
c = 14.2861 (2) Å	μ = 0.12 mm^{-1}
β = 91.7612 (6)°	T = 150 K
V = 1367.92 (3) Å^3	Plate, Clear_pale_colourless
Z = 4	0.48 × 0.16 × 0.10 mm

Data collection

Area diffractometer	2479 reflections with $I > 2.0\sigma(I)$
graphite	$R_{int} = 0.038$
ω scans	$\theta_{max} = 27.5°$, $\theta_{min} = 5.1°$
Absorption correction: Multi-scan *DENZO*/*SCALEPACK* (Otwinowski & Minor, 1997)	$h = -8 \rightarrow 8$
$T_{min} = 0.83$, $T_{max} = 0.99$	$k = -18 \rightarrow 18$
21820 measured reflections	$l = -18 \rightarrow 18$
3120 independent reflections	

Refinement

Refinement on F^2	Primary atom site location: Structure-invariant direct methods
Least-squares matrix: Full	Hydrogen site location: Inferred from neighbouring sites
$R[F^2 > 2\sigma(F^2)] = 0.036$	H-atom parameters constrained
$wR(F^2) = 0.087$	Method = Modified Sheldrick $w = 1/[\sigma^2(F^2) + (0.04P)^2 + 0.63P]$, where $P = (\max(F_o^2,0) + 2F_c^2)/3$
$S = 0.94$	$(\Delta/\sigma)_{max} = 0.001$
3120 reflections	$\Delta\rho_{max} = 0.35$ e Å^{-3}
190 parameters	$\Delta\rho_{min} = -0.31$ e Å^{-3}
0 restraints	

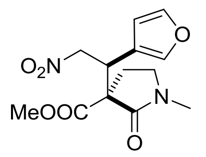

O_2N
MeOOC
N
O
O

(±)-**6e**

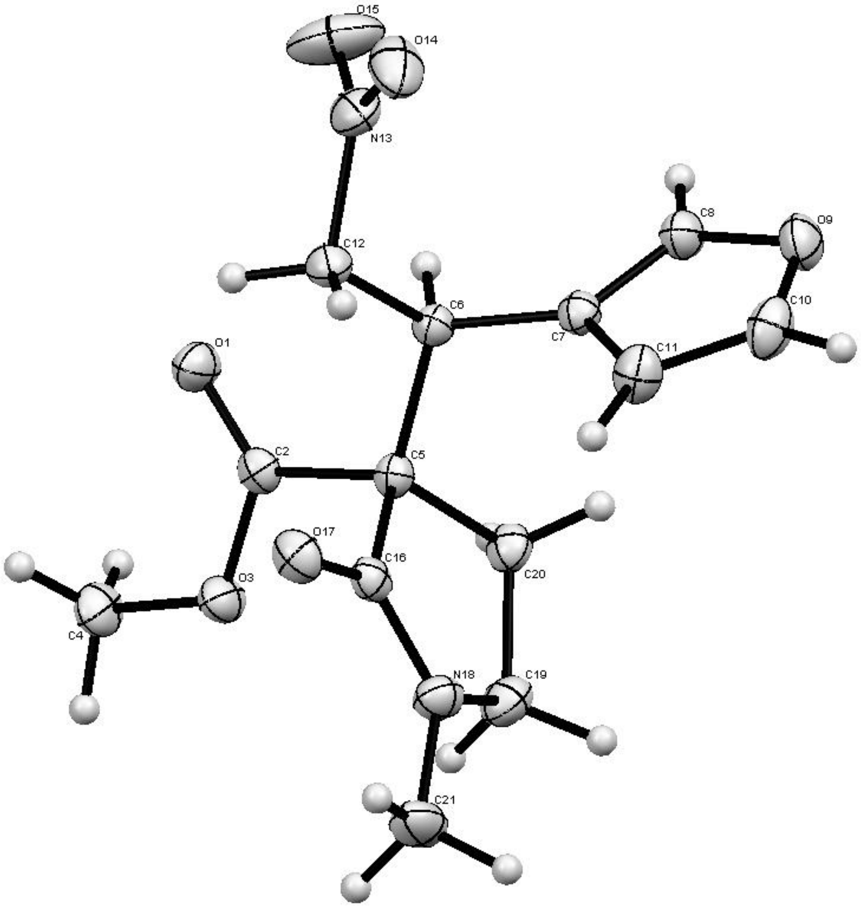

O15
O14
N13
C8
O9
C12
C6
C10
C7
C11
O1
C2
C5
O17
C16
C20
O3
C4
N18
C19
C21

3.2.2. X-ray crystal structure of compound (±)-1g

Crystal data and structure refinement for 1g.

Identification code	s2730abs	
Empirical formula	C19 H21 N3 O6	
Formula weight	387.39	
Temperature	100(2) K	
Wavelength	0.71073 Å	
Crystal system	Orthorhombic	
Space group	$Pna2_1$	
Unit cell dimensions	a = 11.405(2) Å	α= 90°.
	b = 14.943(3) Å	β= 90°.
	c = 10.8676(18) Å	γ = 90°.
Volume	1852.2(6) $Å^3$	
Z	4	
Density (calculated)	1.389 Mg/m^3	
Absorption coefficient	0.105 mm^{-1}	
F(000)	816	
Crystal size	0.55 x 0.50 x 0.50 mm^3	
Theta range for data collection	2.25 to 26.37°.	
Index ranges	-14<=h<=13, -18<=k<=18, -8<=l<=13	
Reflections collected	10299	
Independent reflections	1996 [R(int) = 0.0250]	
Completeness to theta = 26.37°	99.7 %	
Absorption correction	Semi-empirical from equivalents	
Max. and min. transmission	0.9495 and 0.9446	
Refinement method	Full-matrix least-squares on F^2	
Data / restraints / parameters	1996 / 1 / 255	
Goodness-of-fit on F^2	1.091	
Final R indices [I>2sigma(I)]	R1 = 0.0271, wR2 = 0.0667	
R indices (all data)	R1 = 0.0280, wR2 = 0.0672	
Largest diff. peak and hole	0.211 and -0.155 e.$Å^{-3}$	

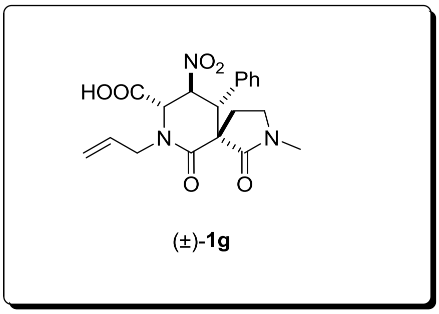

NO2
Ph
HOOC
N
N
O
O
(±)-1g

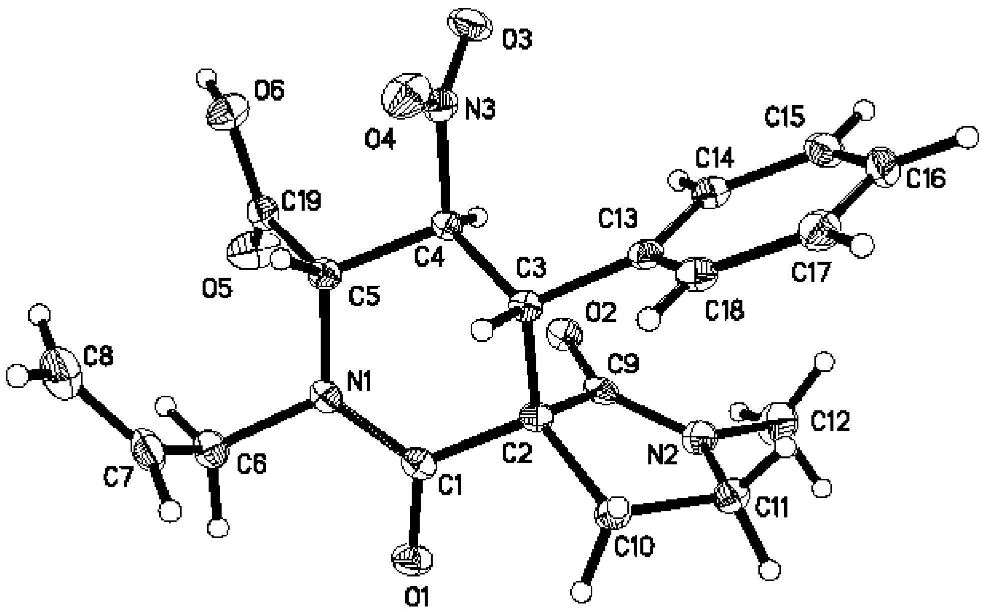

O3
O6
N3
O4
C15
C14
C16
C19
C13
C4
C17
O5
C5
C3
O2
C18
C8
C9
N1
C12
C2
N2
C6
C7
C1
C11
C10
O1

3.2.3. X-ray crystal structure of compound (±)-2a

Crystal data and structure refinement for 2a.

Identification code	s2668abs	
Empirical formula	C23 H23 N3 O4	
Formula weight	405.44	
Temperature	100(2) K	
Wavelength	0.71073 Å	
Crystal system	Monoclinic	
Space group	Pn	
Unit cell dimensions	a = 6.4732(5) Å	α= 90°.
	b = 10.4674(7) Å	β= 95.0500(10)°.
	c = 14.5108(10) Å	γ = 90°.
Volume	979.40(12) $Å^3$	
Z	2	
Density (calculated)	1.375 Mg/m^3	
Absorption coefficient	0.095 mm^{-1}	
F(000)	428	
Crystal size	0.60 x 0.30 x 0.30 mm^3	
Theta range for data collection	1.95 to 26.39°.	
Index ranges	-8<=h<=8, -12<=k<=13, -18<=l<=18	
Reflections collected	7591	
Independent reflections	2000 [R(int) = 0.0141]	
Completeness to theta = 26.39°	99.5 %	
Absorption correction	Semi-empirical from equivalents	
Max. and min. transmission	0.9719 and 0.9449	
Refinement method	Full-matrix least-squares on F^2	
Data / restraints / parameters	2000 / 2 / 272	
Goodness-of-fit on F^2	1.088	
Final R indices [I>2sigma(I)]	R1 = 0.0273, wR2 = 0.0713	
R indices (all data)	R1 = 0.0276, wR2 = 0.0715	
Largest diff. peak and hole	0.235 and -0.167 e.$Å^{-3}$	

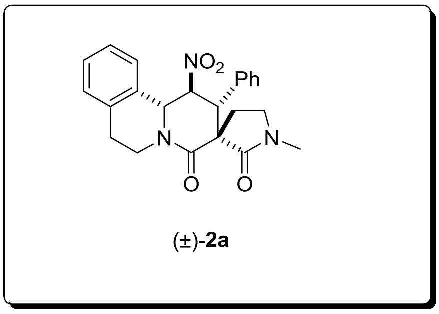

NO2
Ph
N
N
O
O
(±)-2a

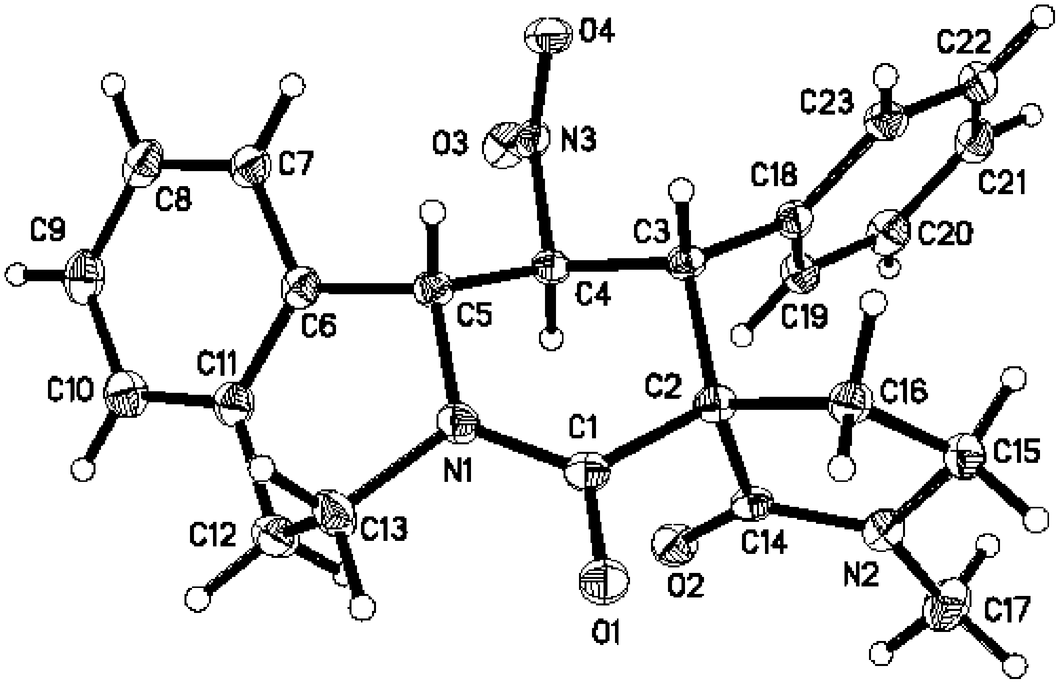

O4
C22
C23
O3
N3
C7
C8
C21
C18
C9
C3
C20
C6
C5
C4
C19
C10
C11
C2
C16
C1
C15
N1
C12
C13
C14
O2
N2
C17
O1

3.2.4. X-ray crystal structure of compound (±)-2a’’

Crystal data and structure refinement for 2a’’.

Identification code	s2685	
Empirical formula	C23 H23 N3 O4	
Formula weight	405.44	
Temperature	100(2) K	
Wavelength	0.71073 Å	
Crystal system	Orthorhombic	
Space group	Pbca	
Unit cell dimensions	a = 15.0761(19) Å	α= 90°.
	b = 10.7200(13) Å	β= 90°.
	c = 23.726(3) Å	γ = 90°.
Volume	3834.5(8) $Å^3$	
Z	8	
Density (calculated)	1.405 Mg/m^3	
Absorption coefficient	0.098 mm^{-1}	
F(000)	1712	
Crystal size	0.45 x 0.40 x 0.05 mm^3	
Theta range for data collection	1.72 to 26.46°.	
Index ranges	-18<=h<=18, -13<=k<=13, -29<=l<=29	
Reflections collected	28298	
Independent reflections	3950 [R(int) = 0.0832]	
Completeness to theta = 26.46°	99.9 %	
Absorption correction	None	
Refinement method	Full-matrix least-squares on F^2	
Data / restraints / parameters	3950 / 0 / 272	
Goodness-of-fit on F^2	0.891	
Final R indices [I>2sigma(I)]	R1 = 0.0416, wR2 = 0.0930	
R indices (all data)	R1 = 0.0718, wR2 = 0.1026	
Largest diff. peak and hole	0.217 and -0.277 e.$Å^{-3}$	

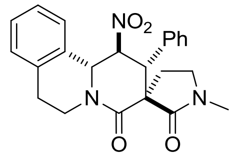
NO2
Ph
N
N
O
O

(±)-**2a''**

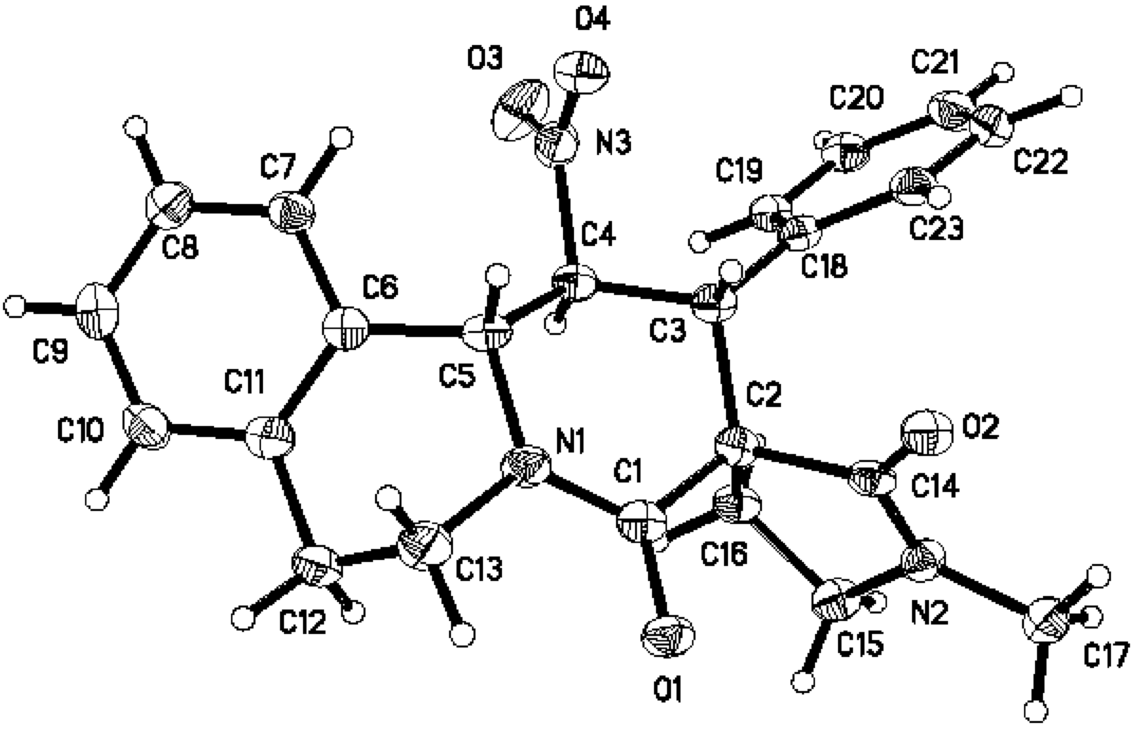
O4
O3
N3
C21
C20
C22
C19
C7
C23
C4
C8
C18
C6
C3
C9
C5
C11
C2
O2
C10
N1
C1
C14
C16
C13
N2
C12
C15
C17
O1

3.2.5. X-ray crystal structure of compound (±)-2m'

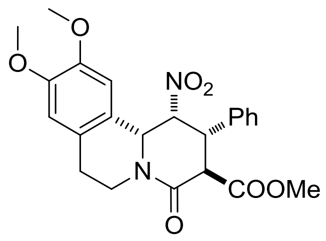

(±)-**2m'**

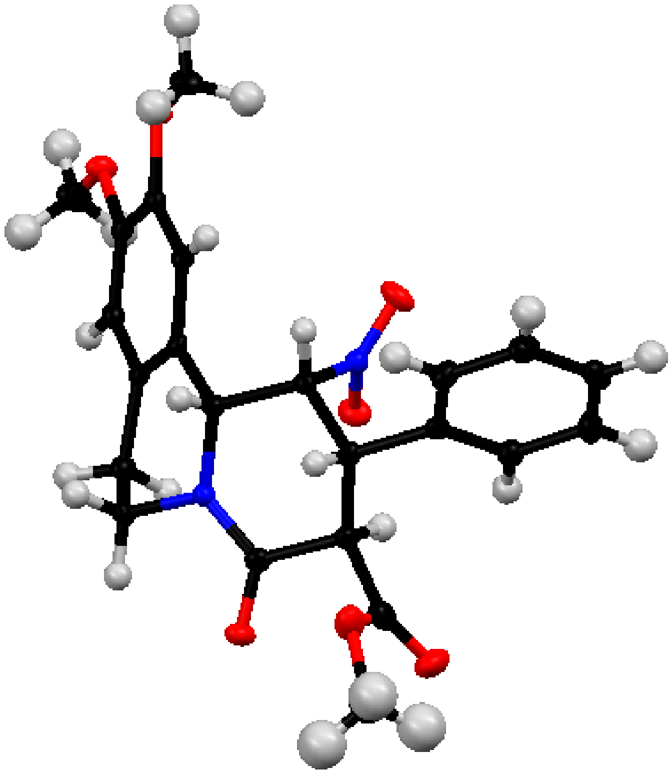

3.2.6. X-ray crystal structure of compound (±)-11c

Crystal data and structure refinement for 11c.

Identification code 6105

Crystal data

$C_{23}H_{26}N_2O$	$F(000) = 744$
$M_r = 346.47$	$D_x = 1.252$ Mg m^{-3}
Monoclinic, $P2_1/c$	Melting point: not measured K
Hall symbol: -P 2ybc	Mo Kα radiation, λ = 0.71073 Å
$a = 20.7471$ (6) Å	Cell parameters from 4193 reflections
$b = 7.3739$ (2) Å	θ = 5–27°
$c = 12.0188$ (3) Å	μ = 0.08 mm^{-1}
β = 90.9587 (14)°	$T = 150$ K
$V = 1838.46$ (9) Å^3	Plate, Clear_pale_colourless
$Z = 4$	0.18 × 0.18 × 0.06 mm

Data collection

Area diffractometer	2739 reflections with $I > 2.0\sigma(I)$
graphite	$R_{int} = 0.058$
ω scans	$\theta_{max} = 27.5°$, $\theta_{min} = 5.1°$
Absorption correction: Multi-scan *DENZO/SCALEPACK* (Otwinowski & Minor, 1997)	$h = -26 \rightarrow 26$
$T_{min} = 0.94$, $T_{max} = 1.00$	$k = -7 \rightarrow 9$
17146 measured reflections	$l = -15 \rightarrow 15$
4194 independent reflections	

Refinement

$R[F^2 > 2\sigma(F^2)] = 0.050$	0 restraints
$wR(F^2) = 0.156$	No H atoms present
$S = 0.97$	$\Delta\rho_{max} = 0.44$ e Å^{-3}
4194 reflections	$\Delta\rho_{min} = -0.39$ e Å^{-3}
235 parameters	

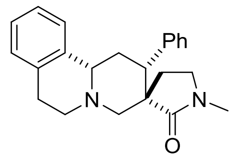
Ph
N
N
O

(±)-**11c**

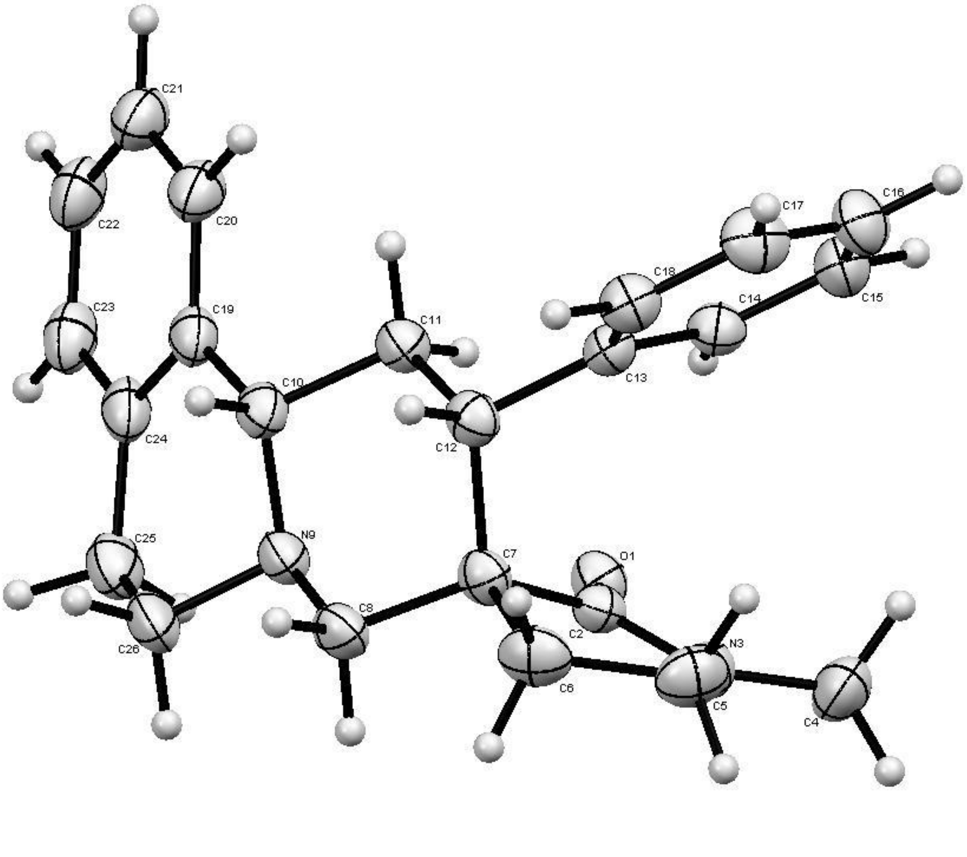
C21
C22
C20
C17
C16
C18
C14
C15
C23
C19
C11
C13
C10
C24
C12
C25
N9
C7
O1
C8
C2
N3
C26
C6
C5
C4

4. References

1. Cosier, J.; Glazer, A. M. *J. Appl. Cryst* **1986**, 19, 105–107.
2. Otwinowski, Z.; Minor, W. *Methods Enzymol.* **1997**, 276, 307–326.
3. Sheldrick, G. M. *Acta Cryst.* **2008**, A64, 112–122.
4. Burla, M. C.; Caliandro, R.; Camalli, M.; Carrozzini, B.; Cascarano, G. L.; De Caro, L.; Giacovazzo, C.; Polidori, G.; Spagna, R. *J. Appl. Cryst.* **2005**, 38, 381–388.
5. Betteridge, P. W.; Carruthers, J. R.; Cooper, R. I.; Prout, K.; Watkin, D. J. *J. Appl. Cryst.* **2003**, 36, 1487.
6. Kyle, A. F.; Jakubec, P.; Cockfield, D. M.; Cleator, E.; Skidmore, J.; Dixon, D. J. *Chem. Commun.* **2011**, *47*, 10037–10039.
7. Jakubec, P.; Helliwell, M.; Dixon, D. J. *Org. Lett.* **2008**, *10*, 4267–4270.
8. Quesada, M. L.; Schlessinger, R. H. *J. Org. Chem.* **1978**, *43*, 346–347.
9. Elliott, M. C.; Williams, E. *Org. Biol . Chem.* **2003**, *1*, 3038–3047.
10. E. P. Kohler, H. Engelbrecht, *J. Am. Chem. Soc.* **1919**, *41*, 764–770.
11. Jakubec, P.; Kyle, A. F.; Calleja, J.; Dixon, D. J. *Tetrahedron Lett.* **2011**, *52*, 6094–6097.
12. Ye, J.; Dixon, D. J.; Hynes, P. S. *Chem. Commun.* **2005**, 4481–4483.
13. Okino, T.; Hoashi, Y.; Takemoto, Y. *J. Am. Chem. Soc.* **2003**, *125*, 12672–12673.
14. Watanabe, M.; Ikagawa, A.; Wang, H.; Murata, K.; Ikariya, T. *J. Am. Chem. Soc.* **2004**, *126*, 11148–11149.
15. E. P. Kohler, H. Engelbrecht, *J. Am. Chem. Soc.* **1919**, *41*, 764–770.
16. A mixture of two major diastereomers **2m**/**2m’** epimeric at the stereogenic centre bearing the nitro-group (C-5) was observed after 2 hours reaction time (49:51) under the described conditions. After 24 hours, the diastereomeric ratio had changed dramatically (7:93) in the biphasic mixture (suspension). Subsequently the reaction mixture was cooled to room temperature and **2m** and **2m’** were isolated by columun chromatography as single diastereomers.

5. ^{1}H and ^{13}C NMR Spectra

5.1. ^{1}H NMR spectra of 6b

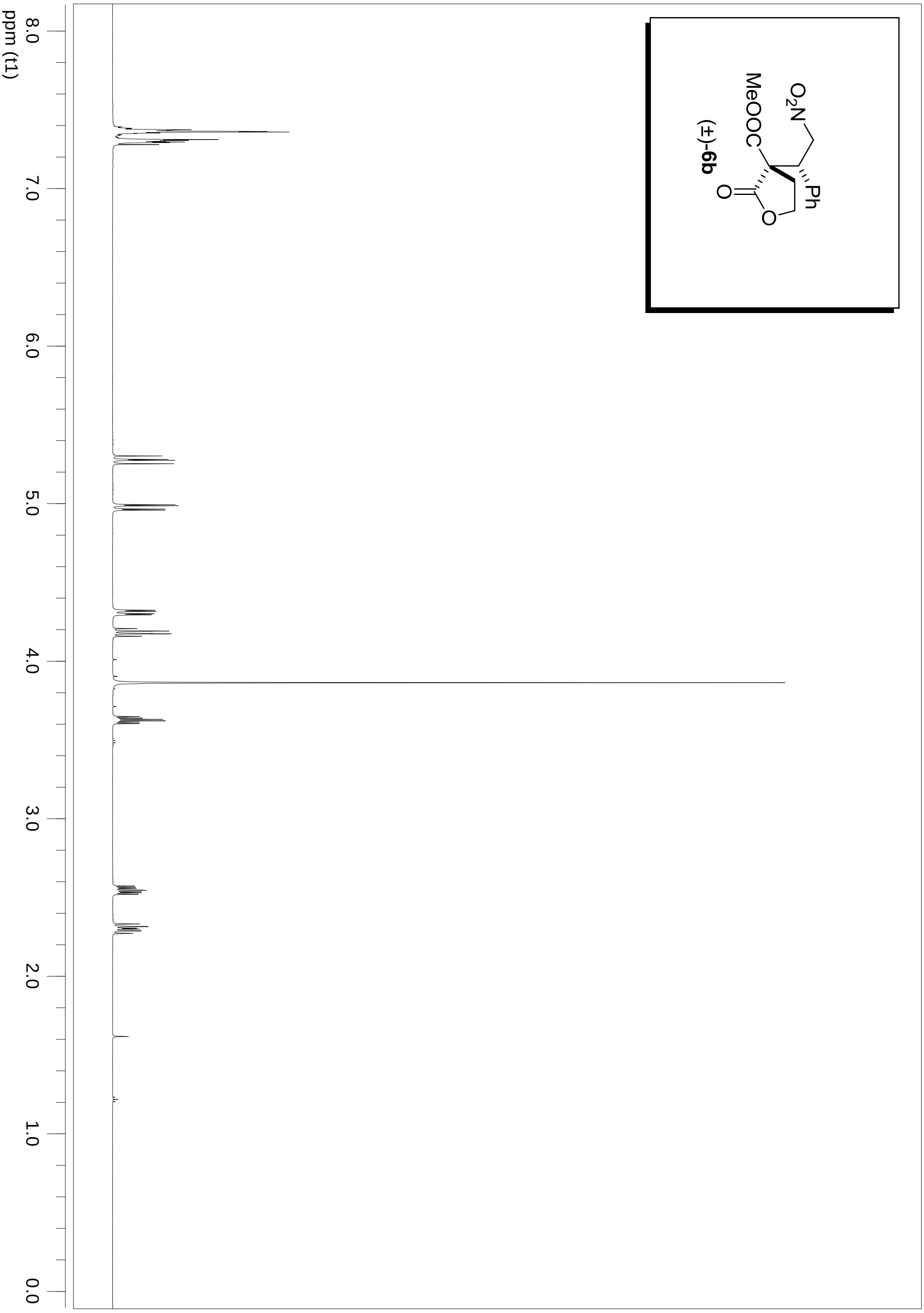

5.1. ^{13}C NMR spectra of 6b

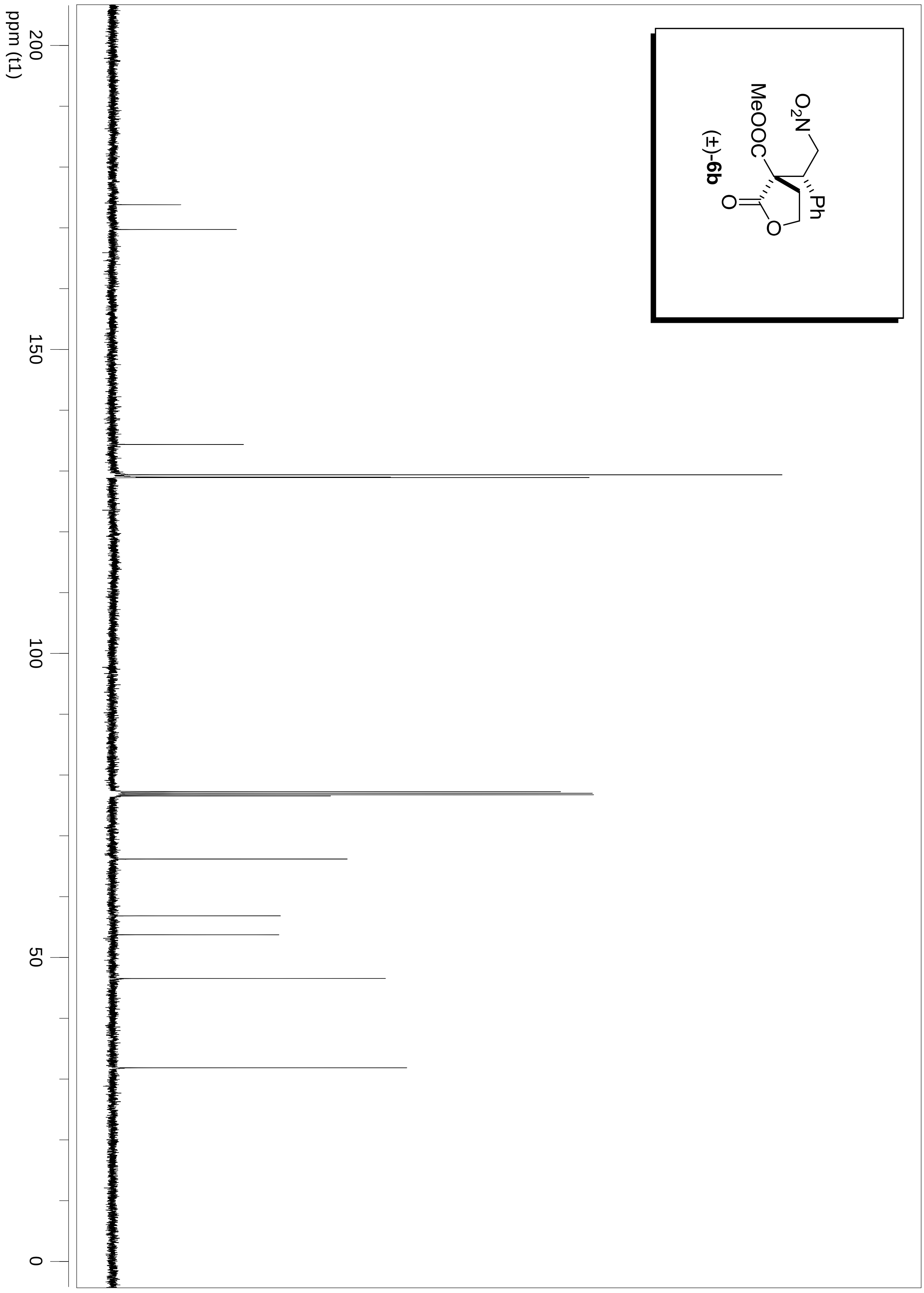

5.2. ^{1}H NMR spectra of 6c

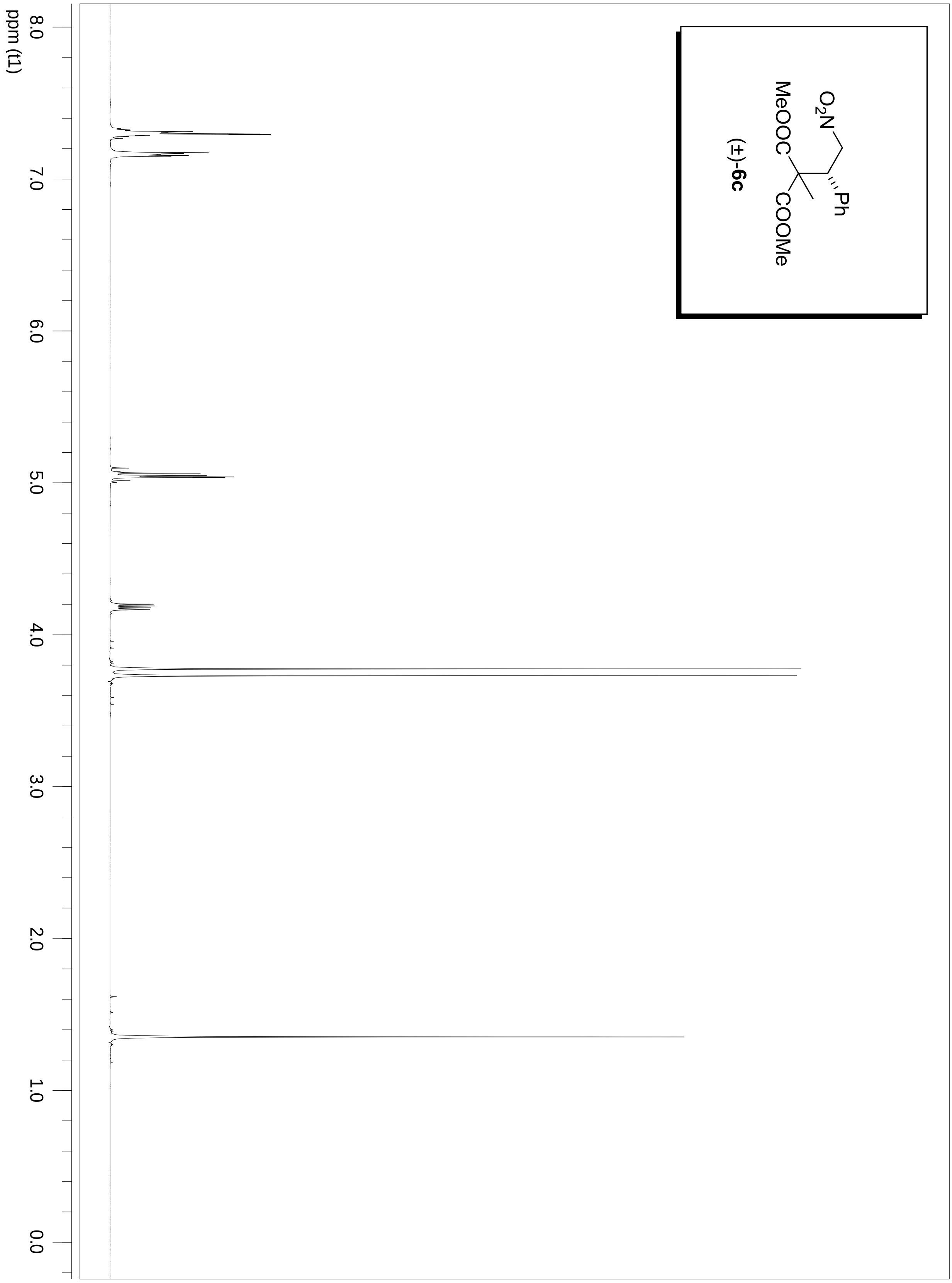

5.2. ^{13}C NMR spectra of 6c

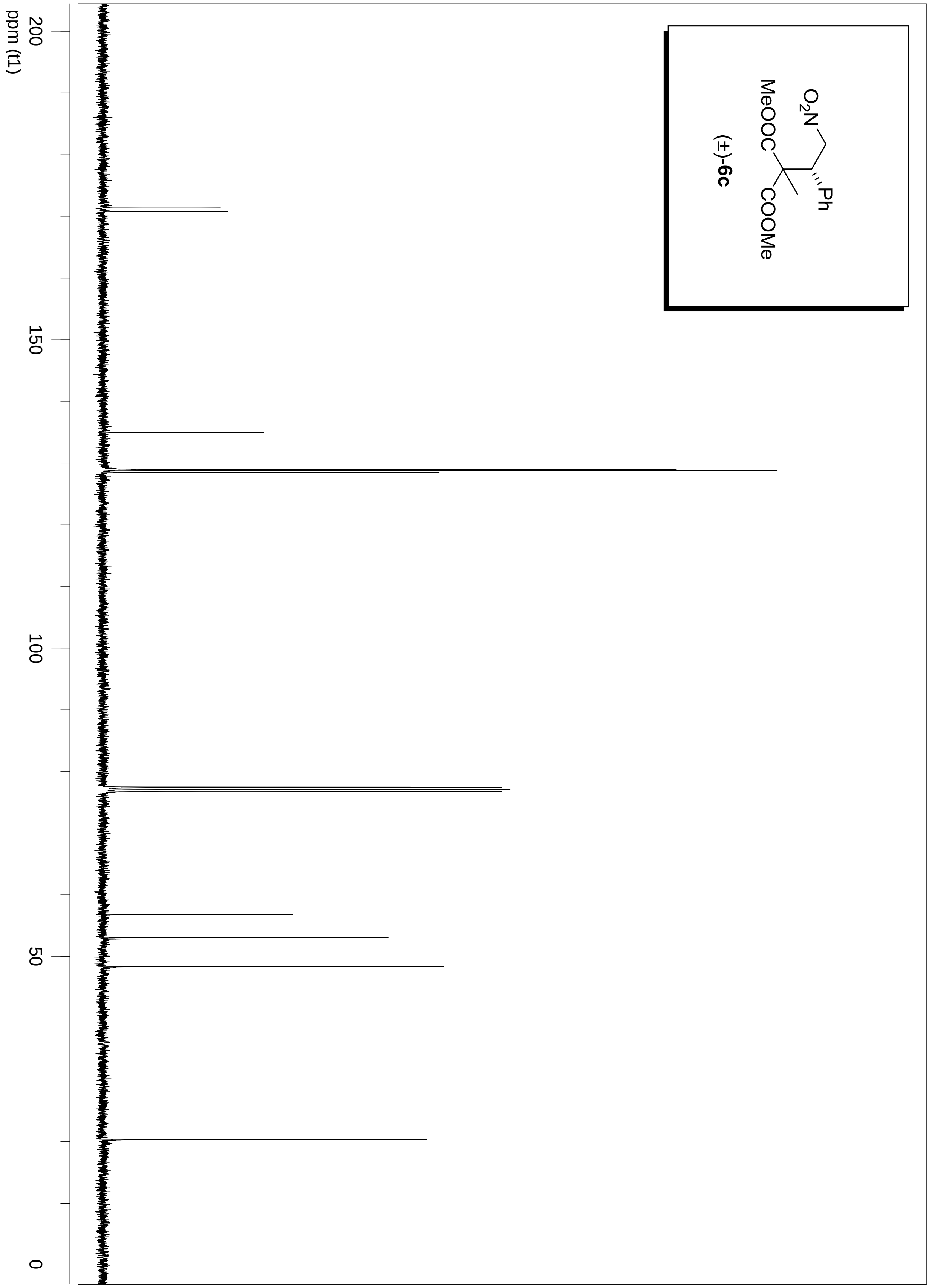

5.3. ^{1}H NMR spectra of 6d

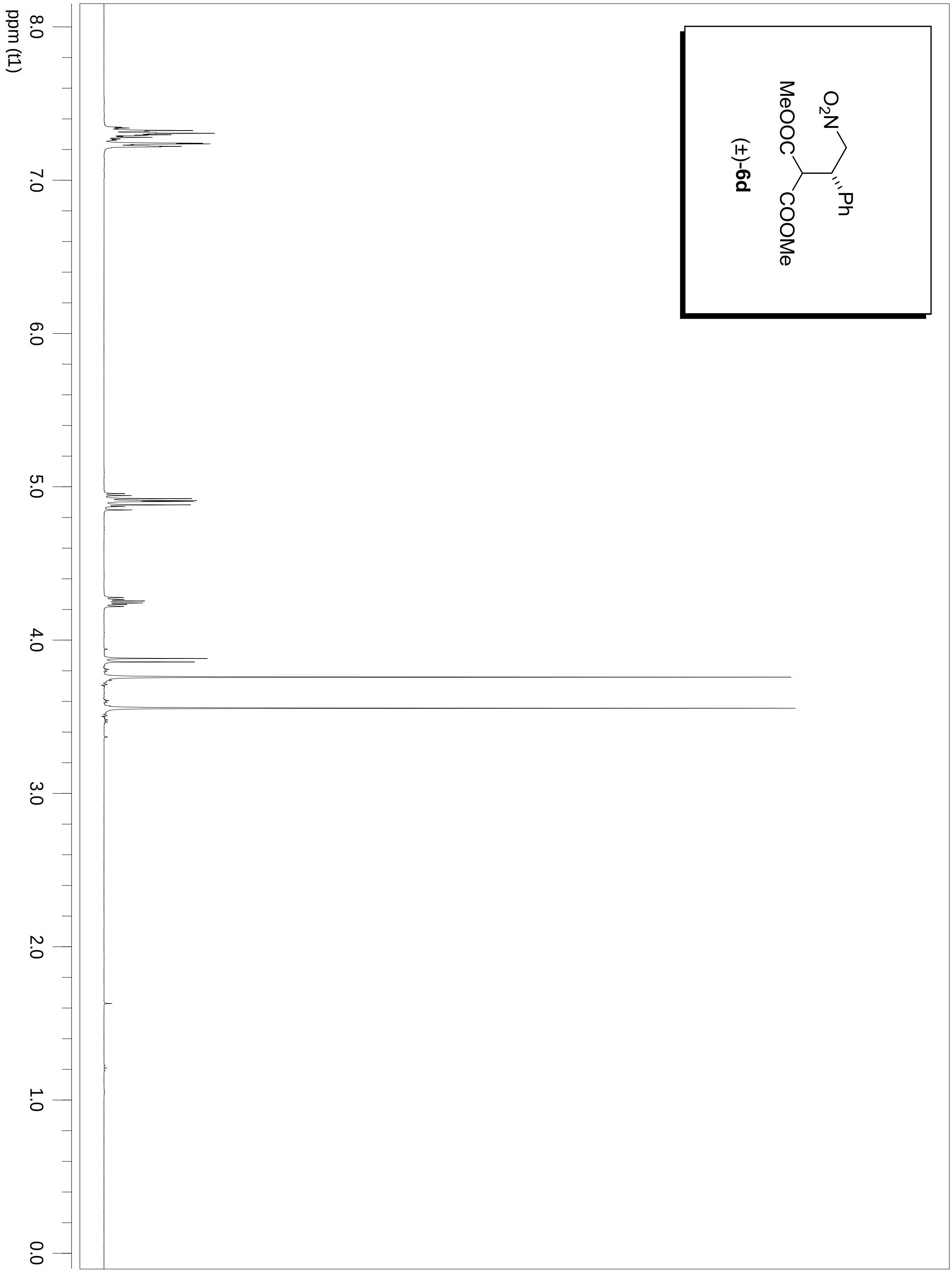

5.3. ^{13}C NMR spectra of 6d

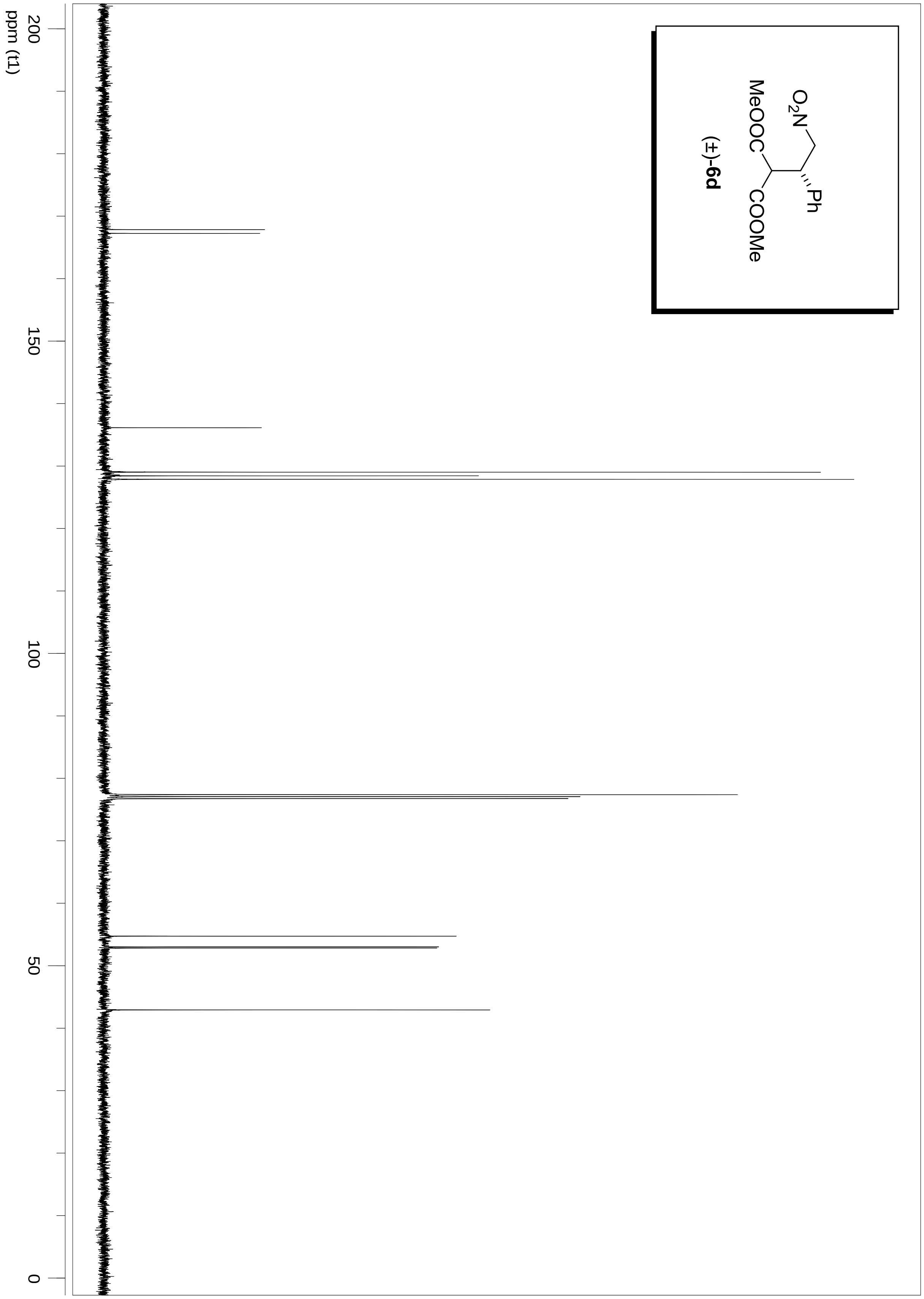

5.4. ^{1}H NMR spectra of 6e

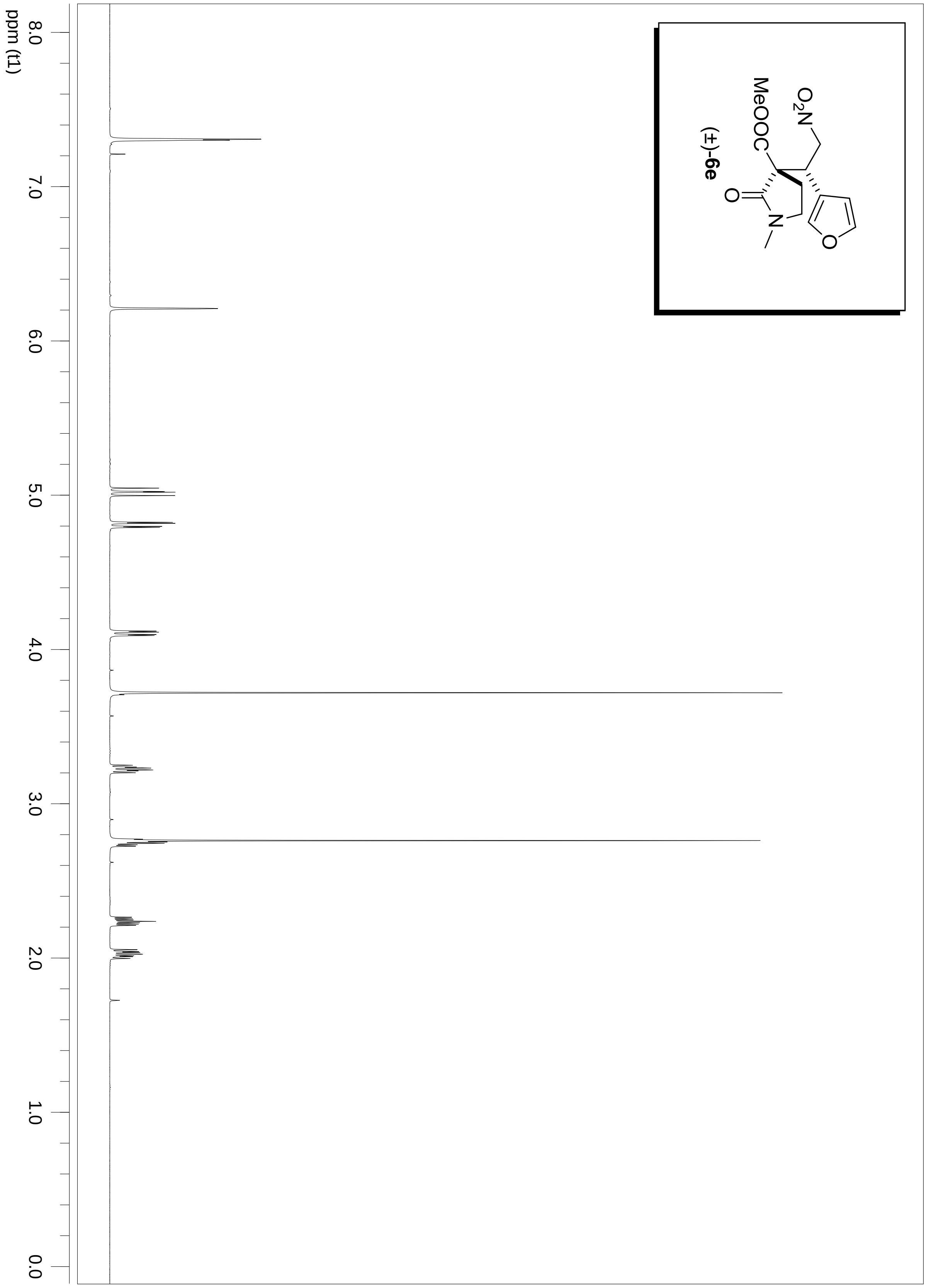

5.4. ^{13}C NMR spectra of 6e

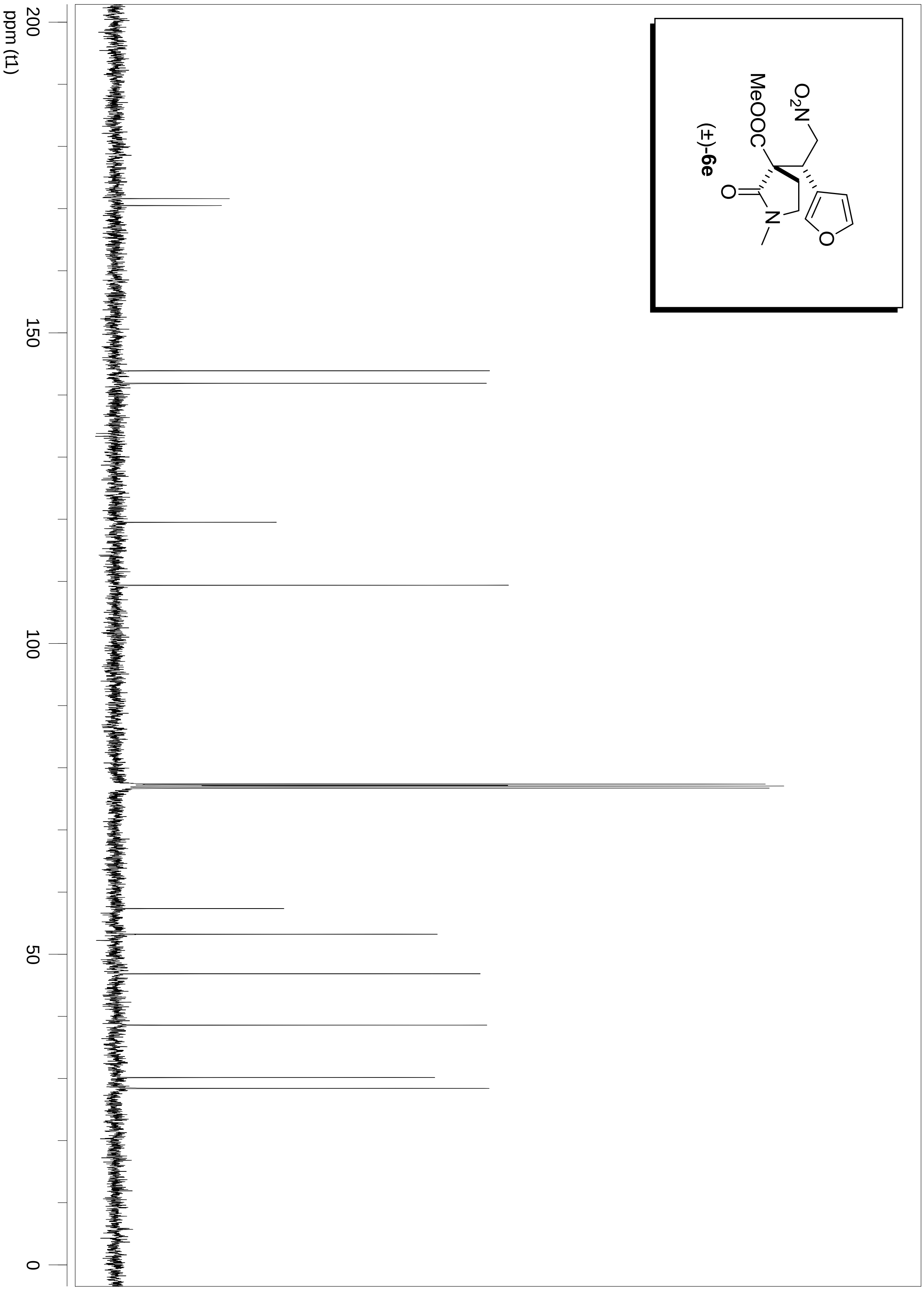

5.5. ^{1}H NMR spectra of 1a

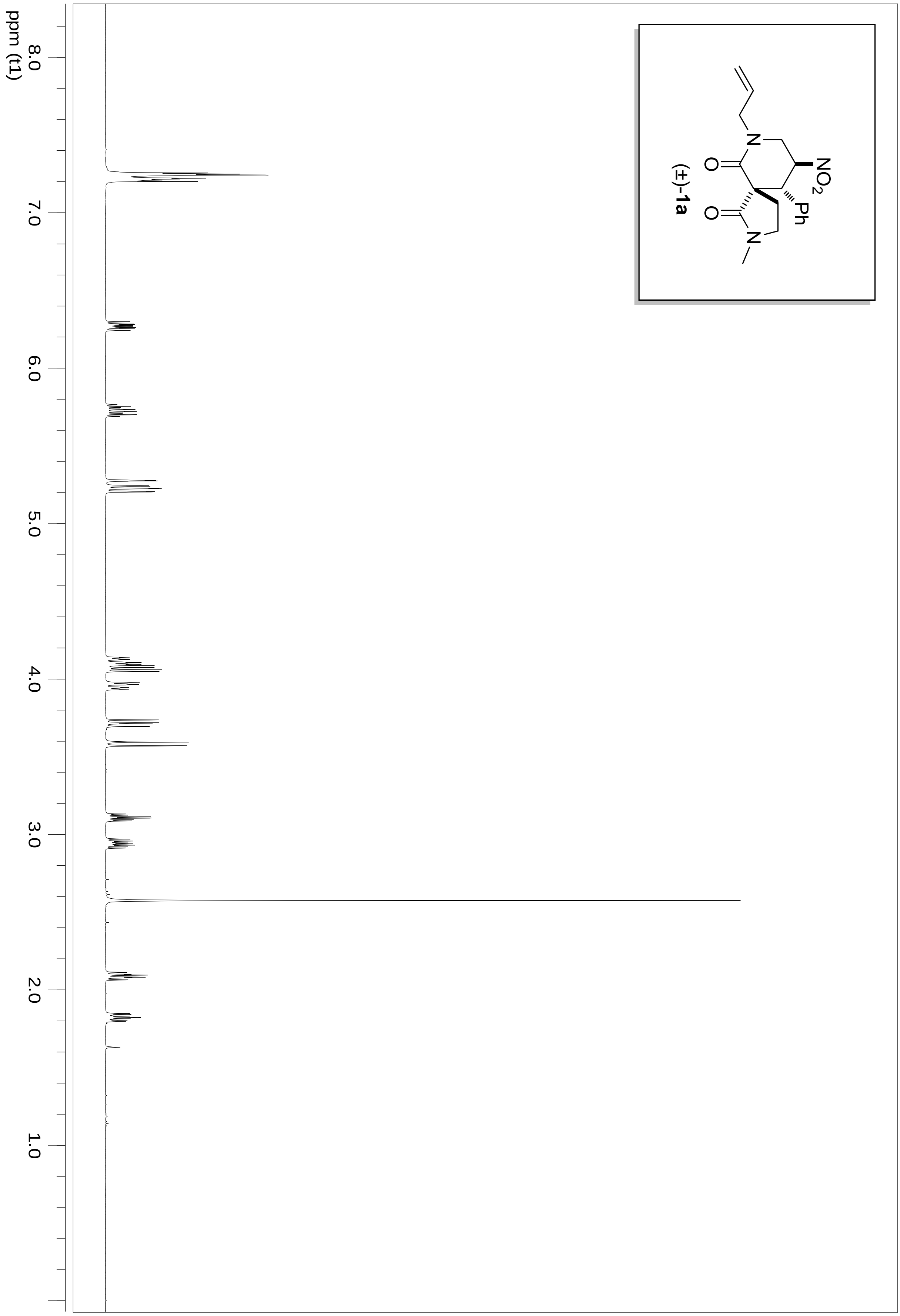

5.5. ^{13}C NMR spectra of 1a

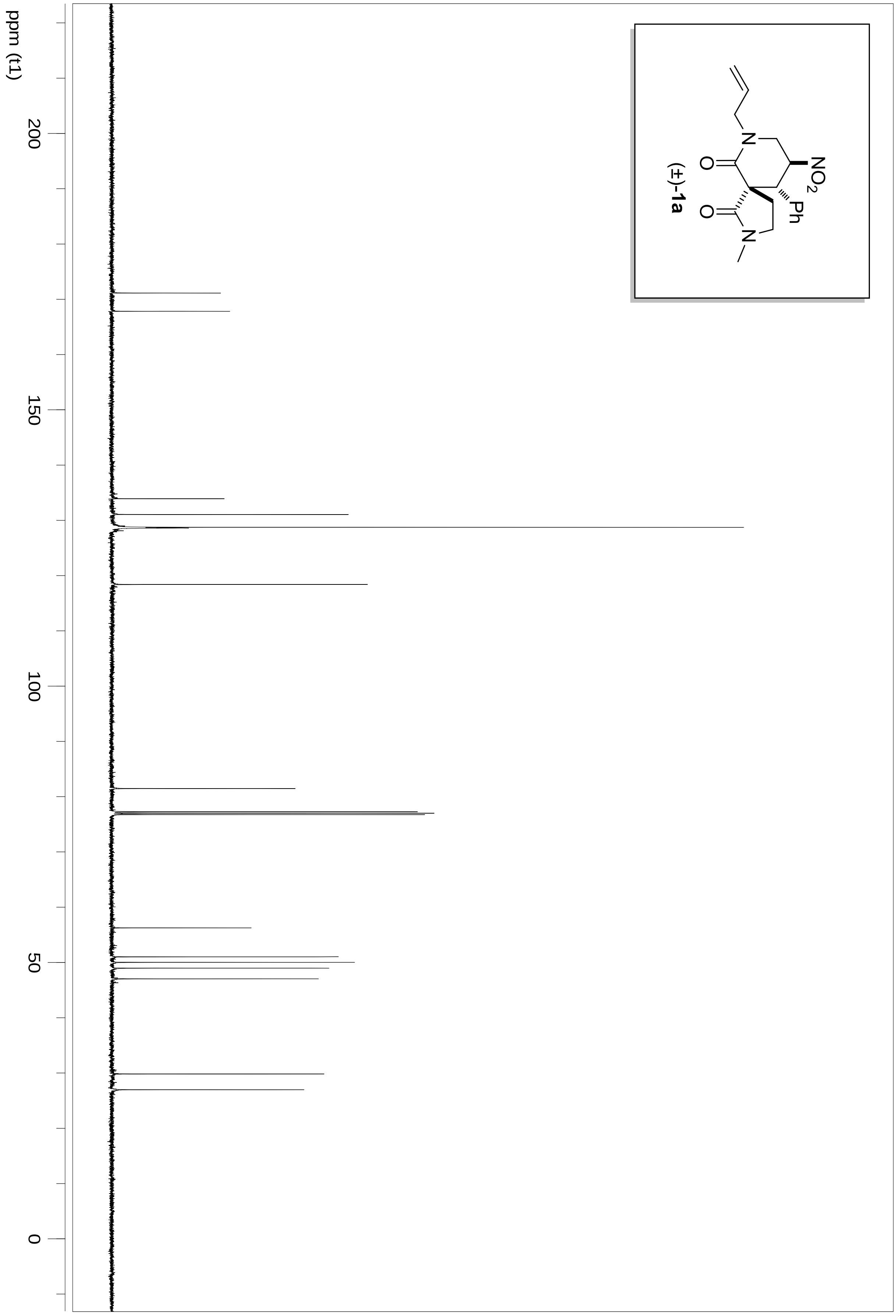

5.6. ^{1}H NMR spectra of 1b

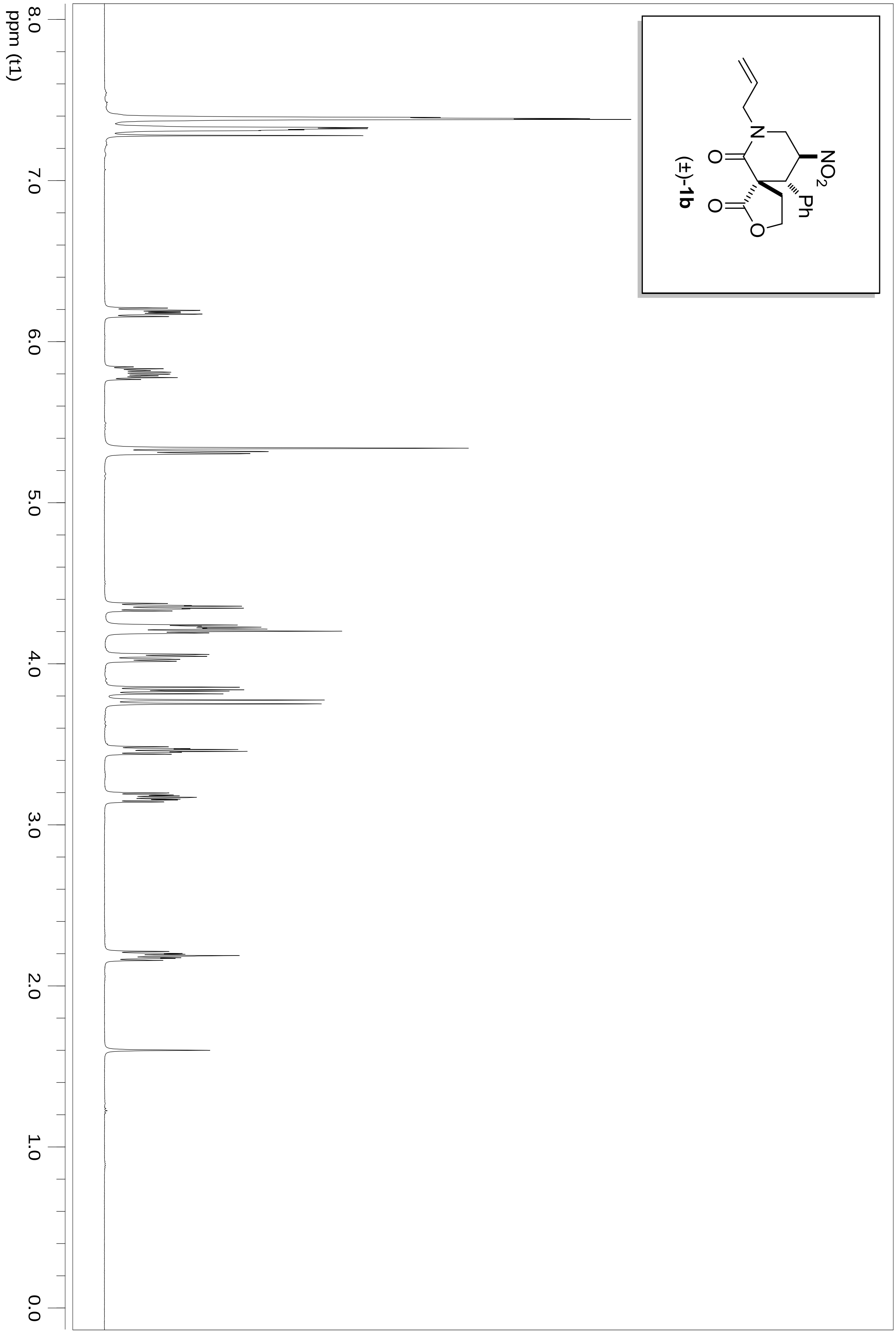

5.6. ^{13}C NMR spectra of 1b

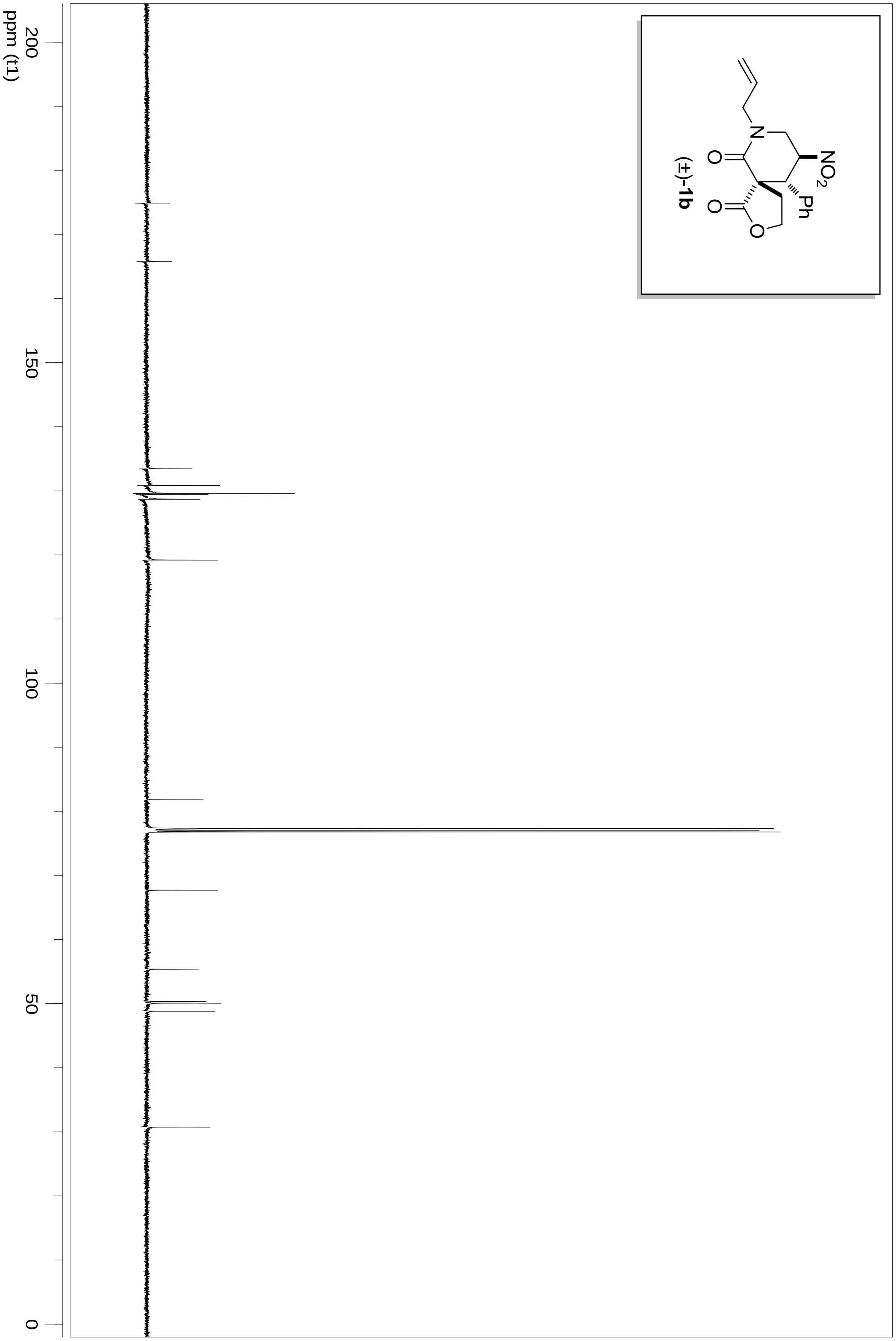

5.7. ^{1}H NMR spectra of 1c

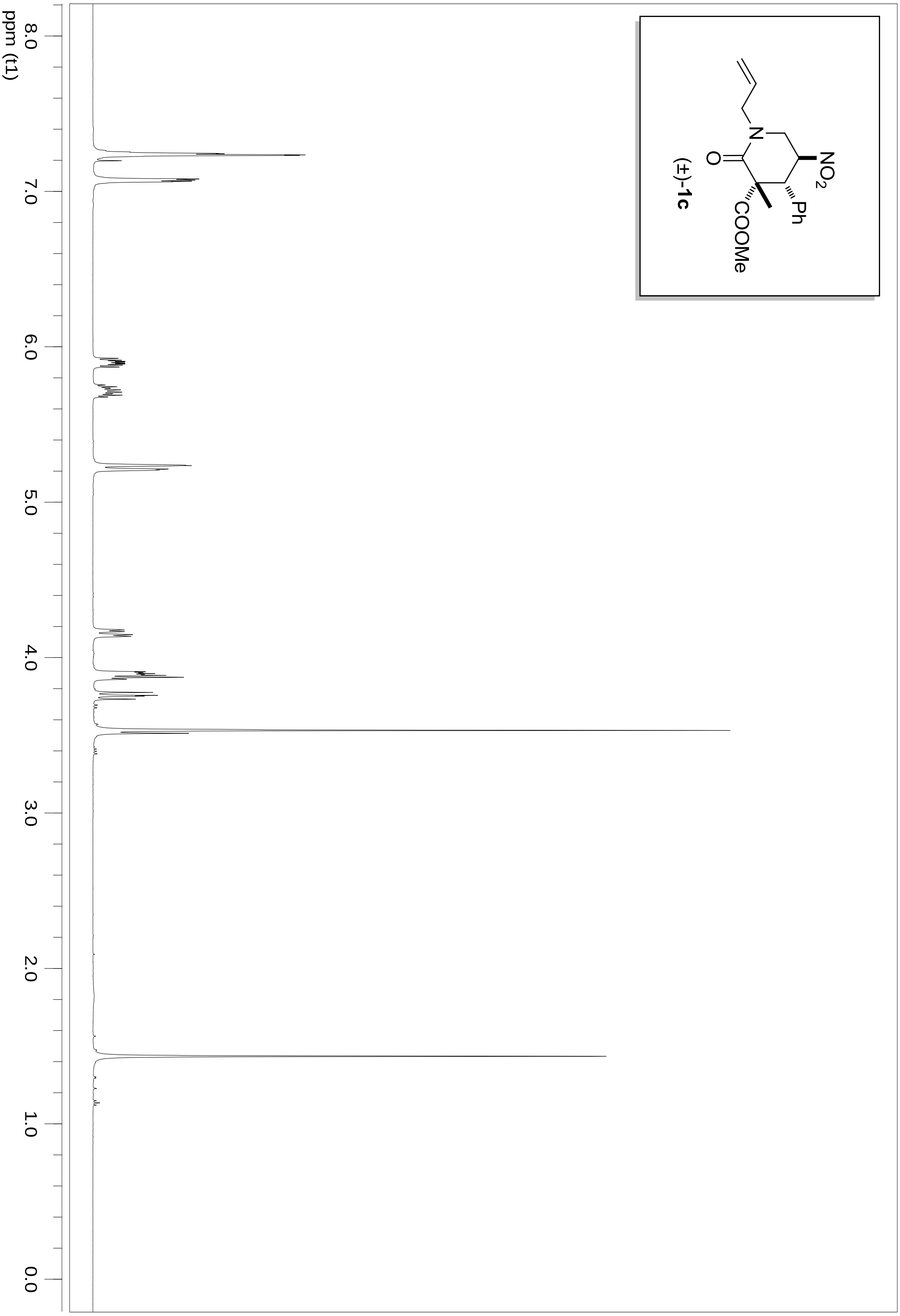

5.7. ^{13}C NMR spectra of 1c

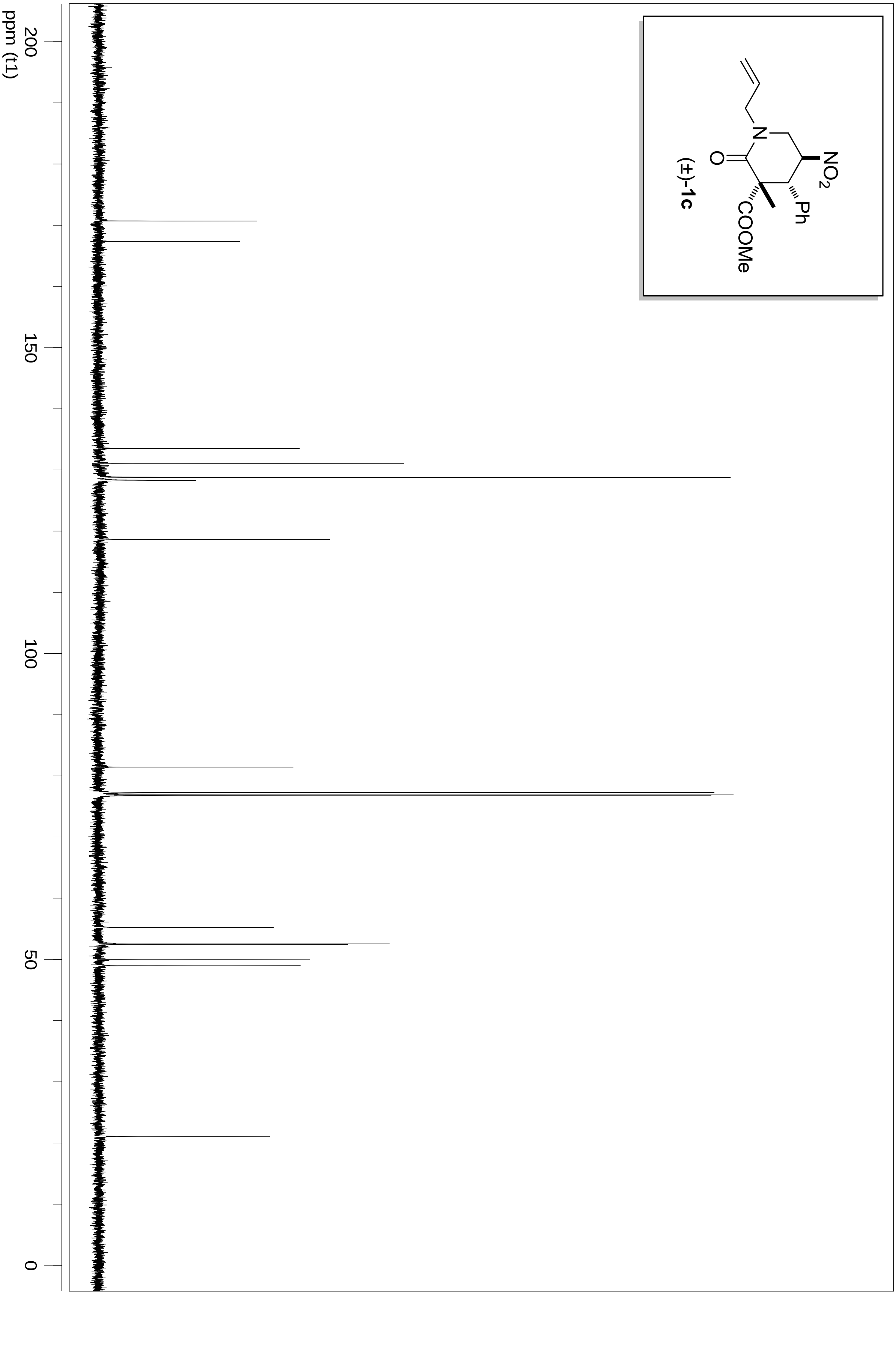

5.8. ^1H NMR spectra of 1d

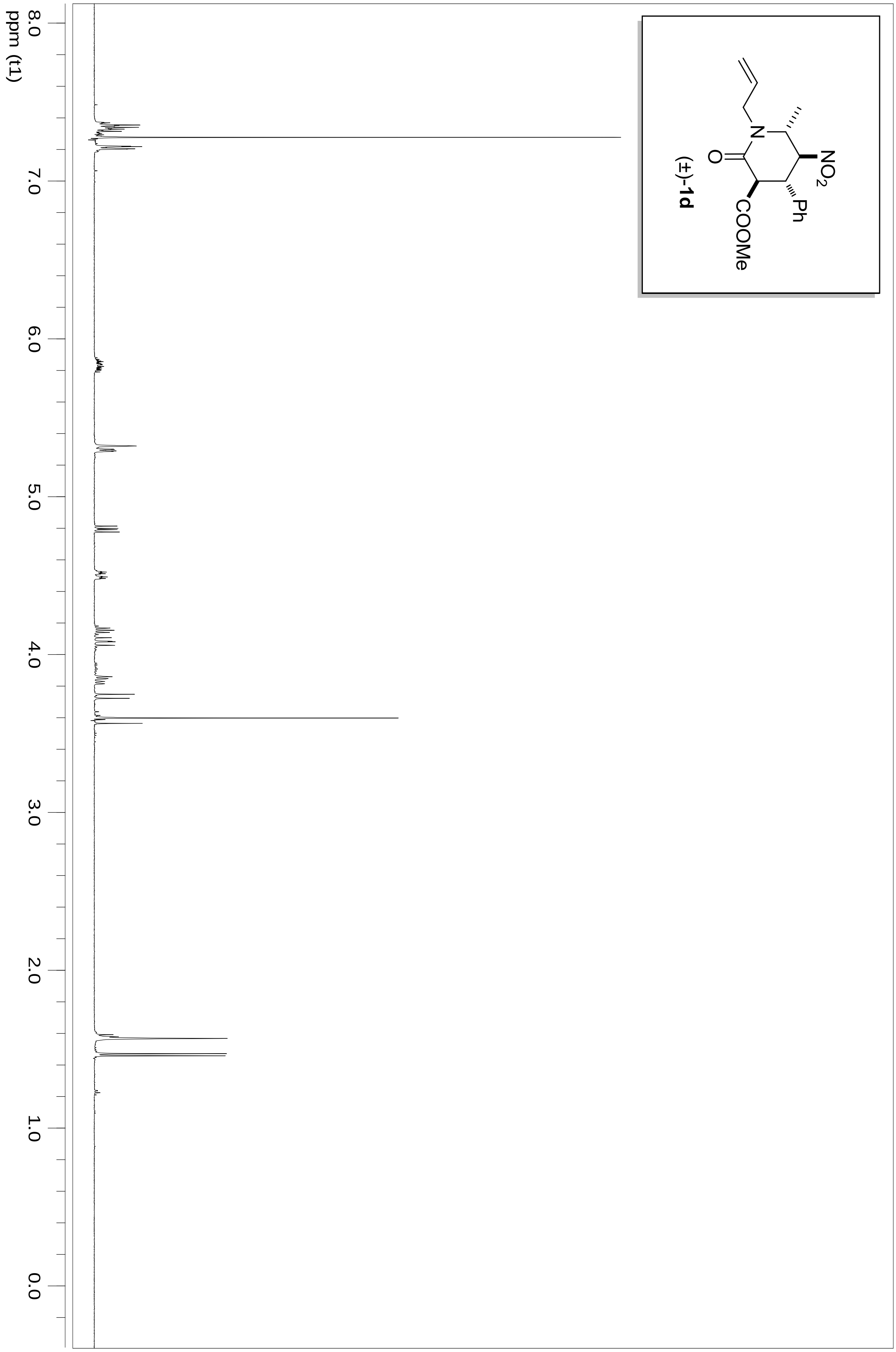

5.8. ^{13}C NMR spectra of 1d

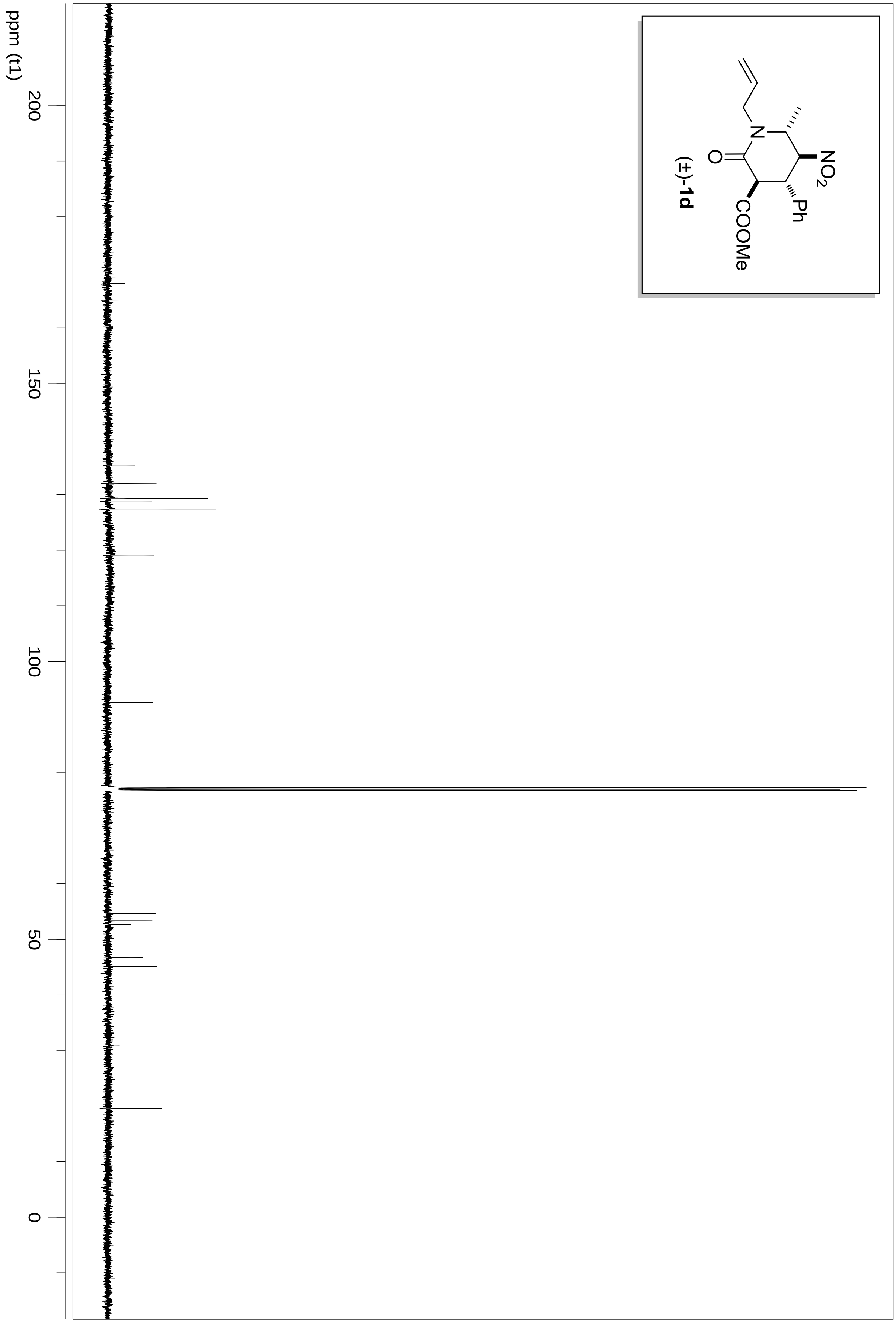

5.9

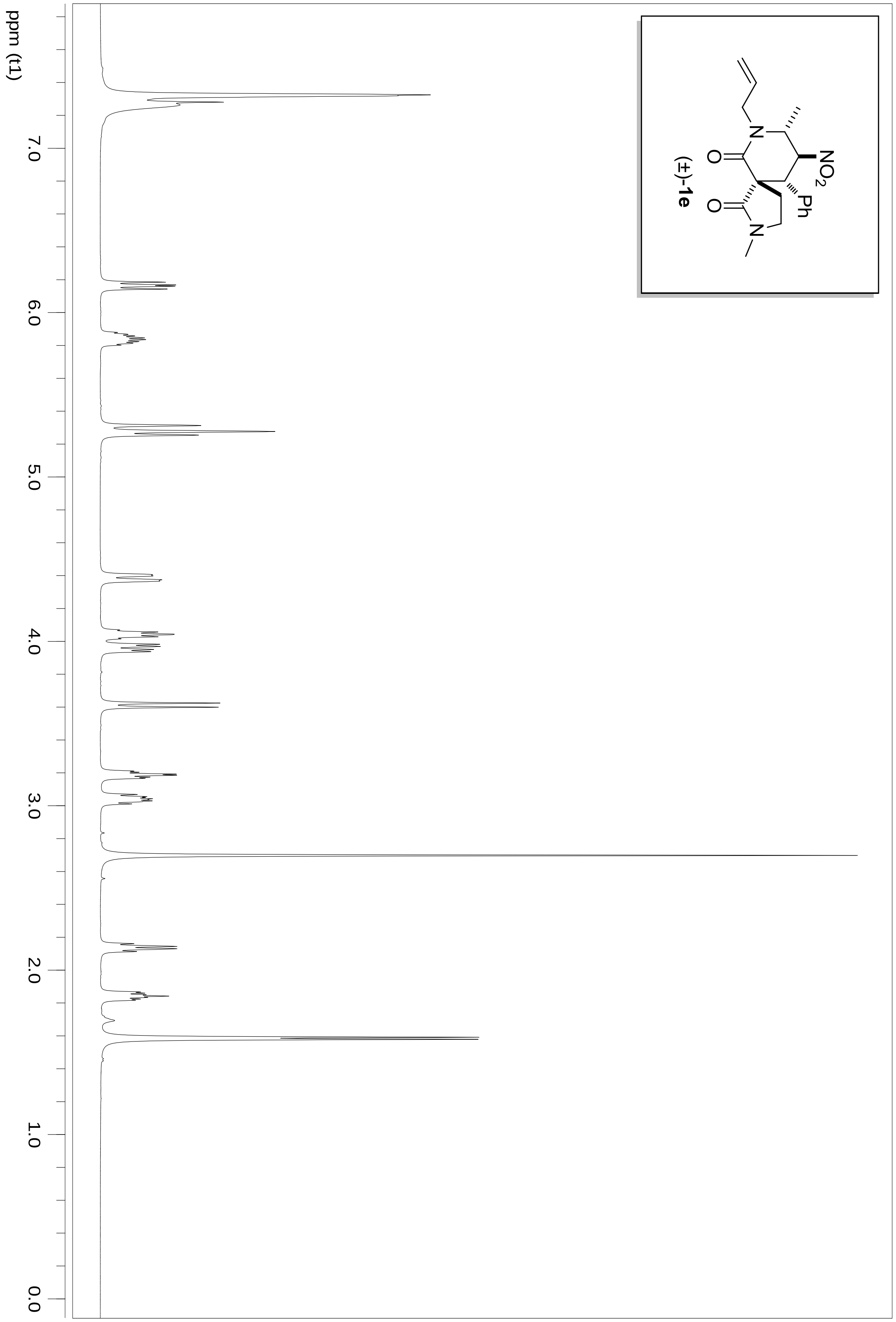

5.9. ^{13}C NMR spectra of 1e

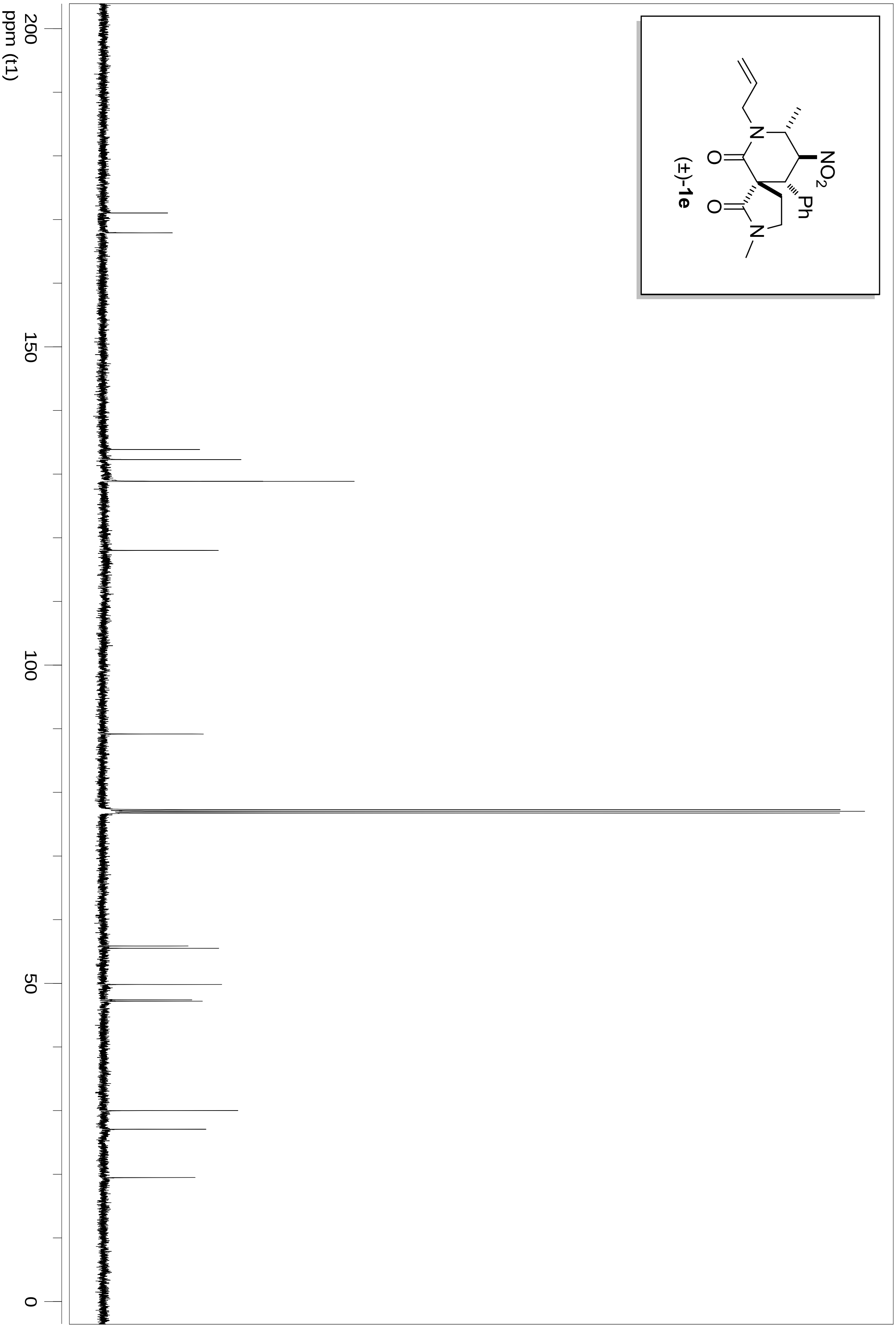

5.10. ^{1}H NMR spectra of 1f

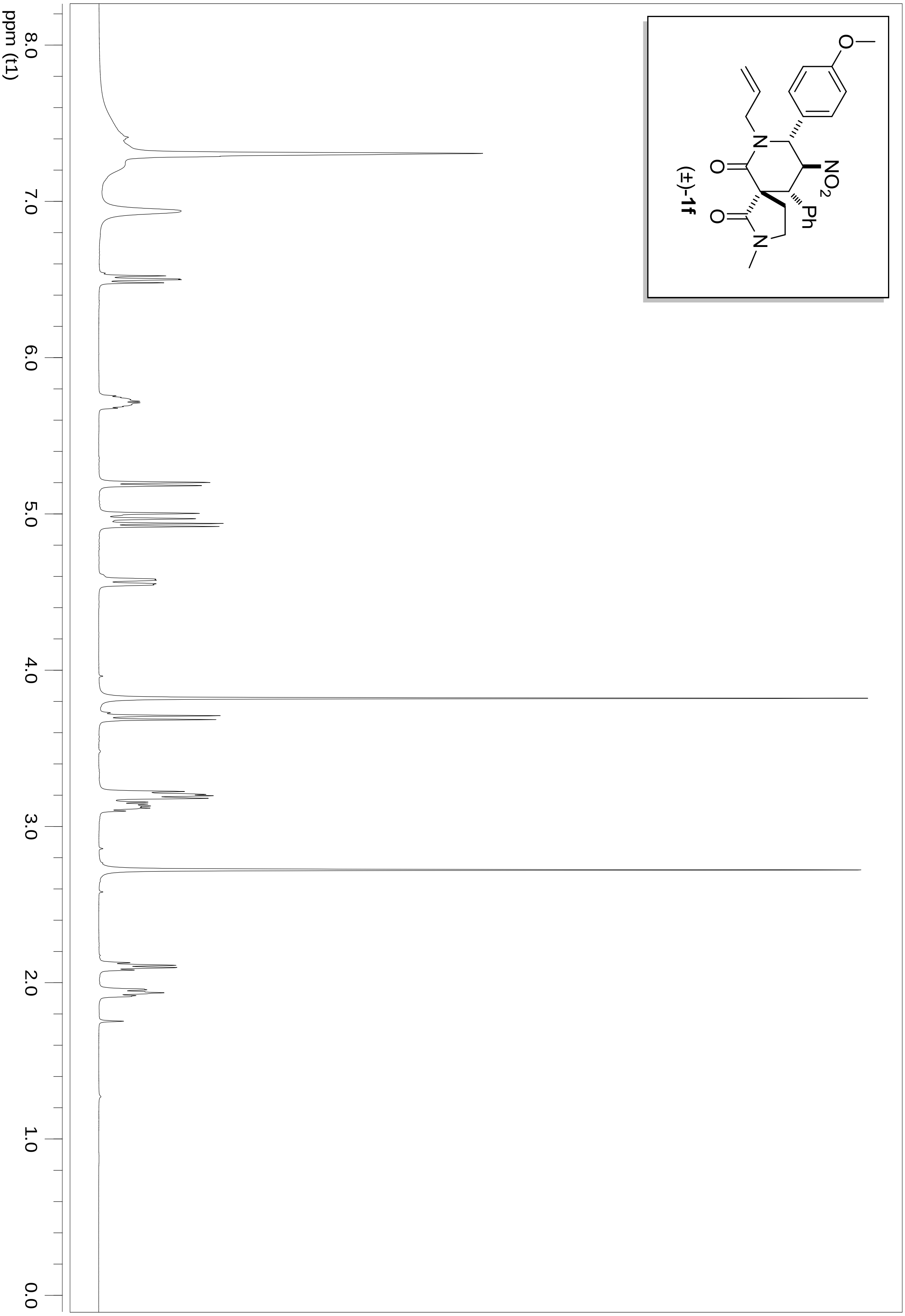

5.10. ^{13}C NMR spectra of 1f

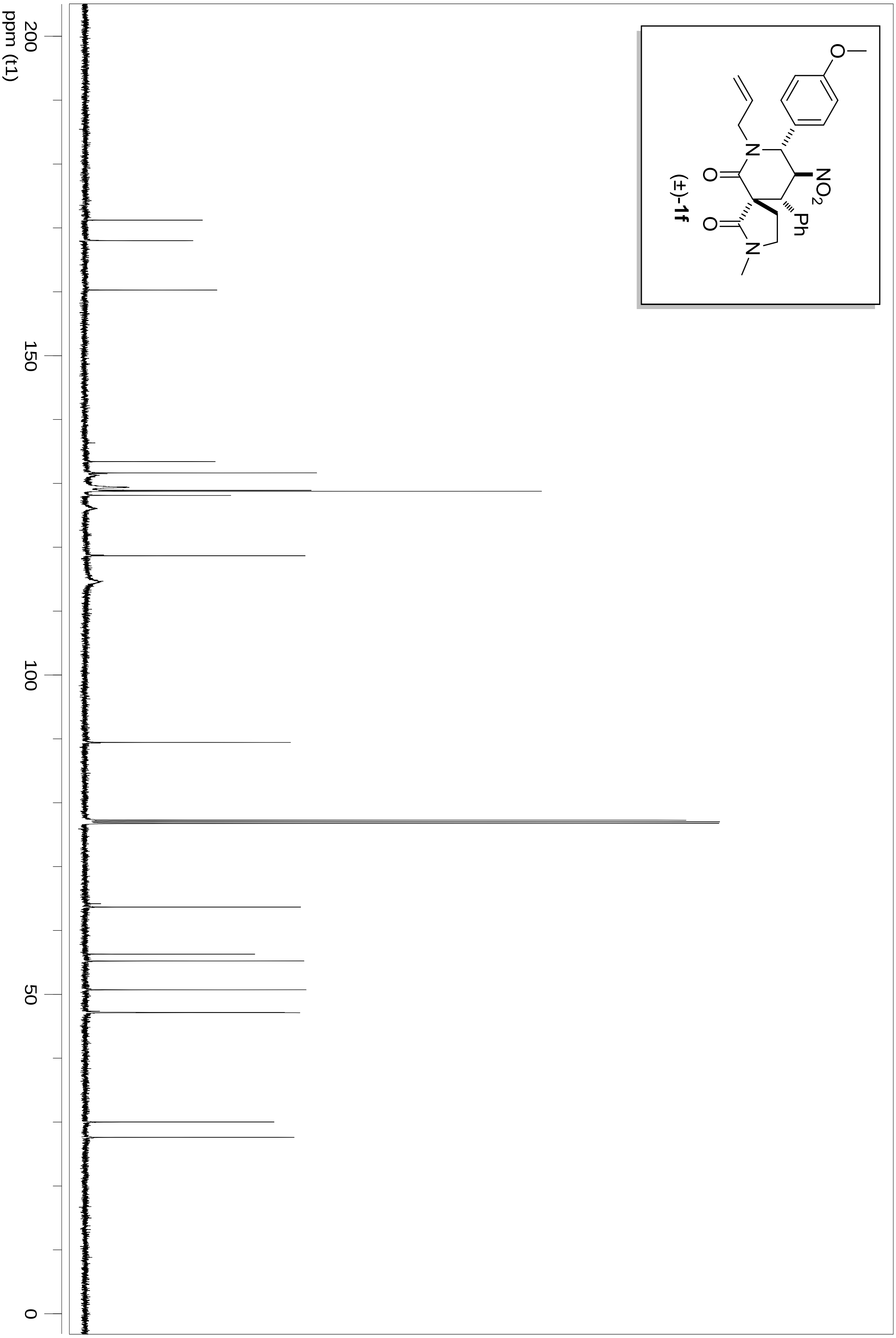

5.11. ^{1}H NMR spectra of 1g

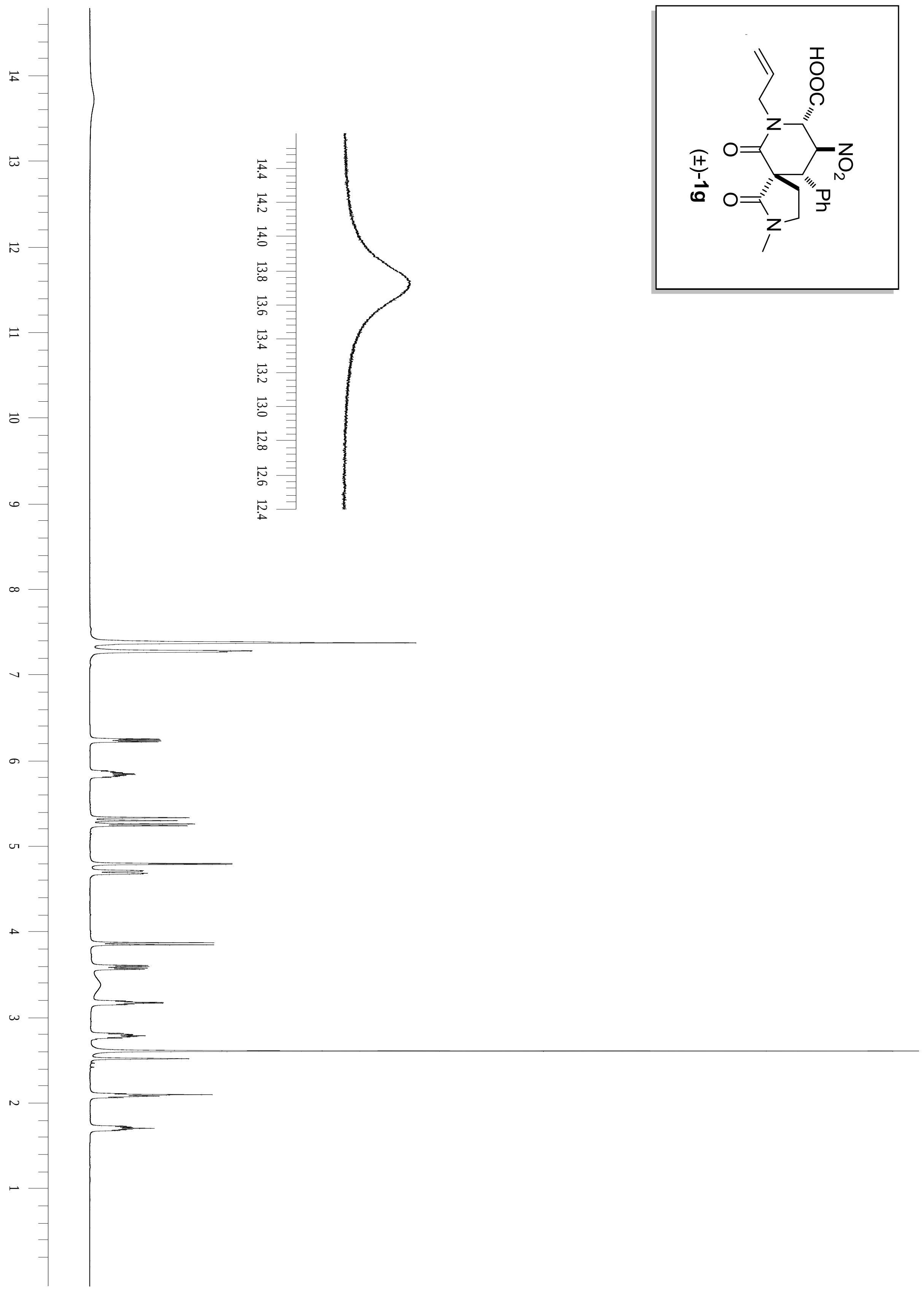

5.11. ^{13}C NMR spectra of 1g

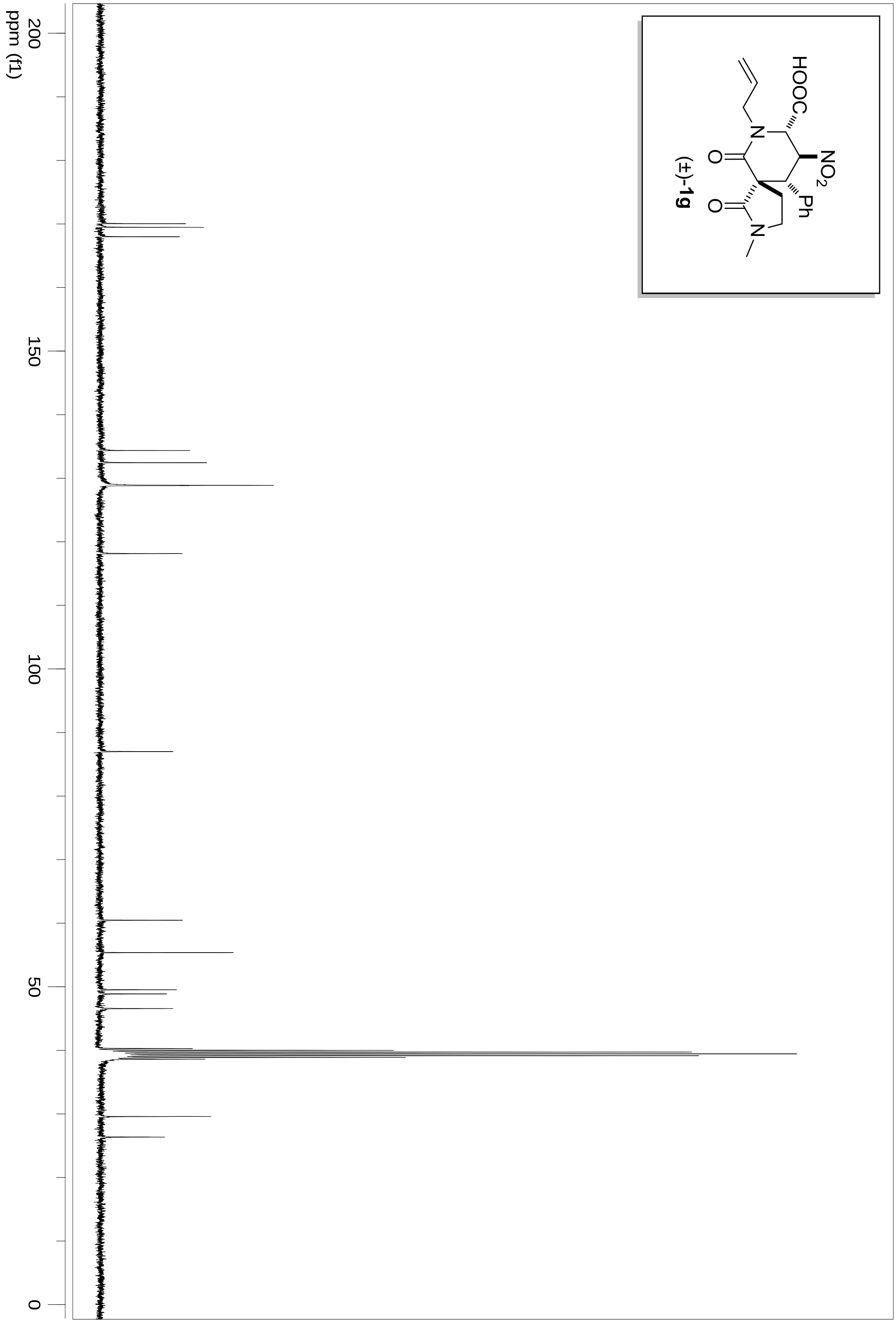

5.12. ^{1}H NMR spectra of 1h

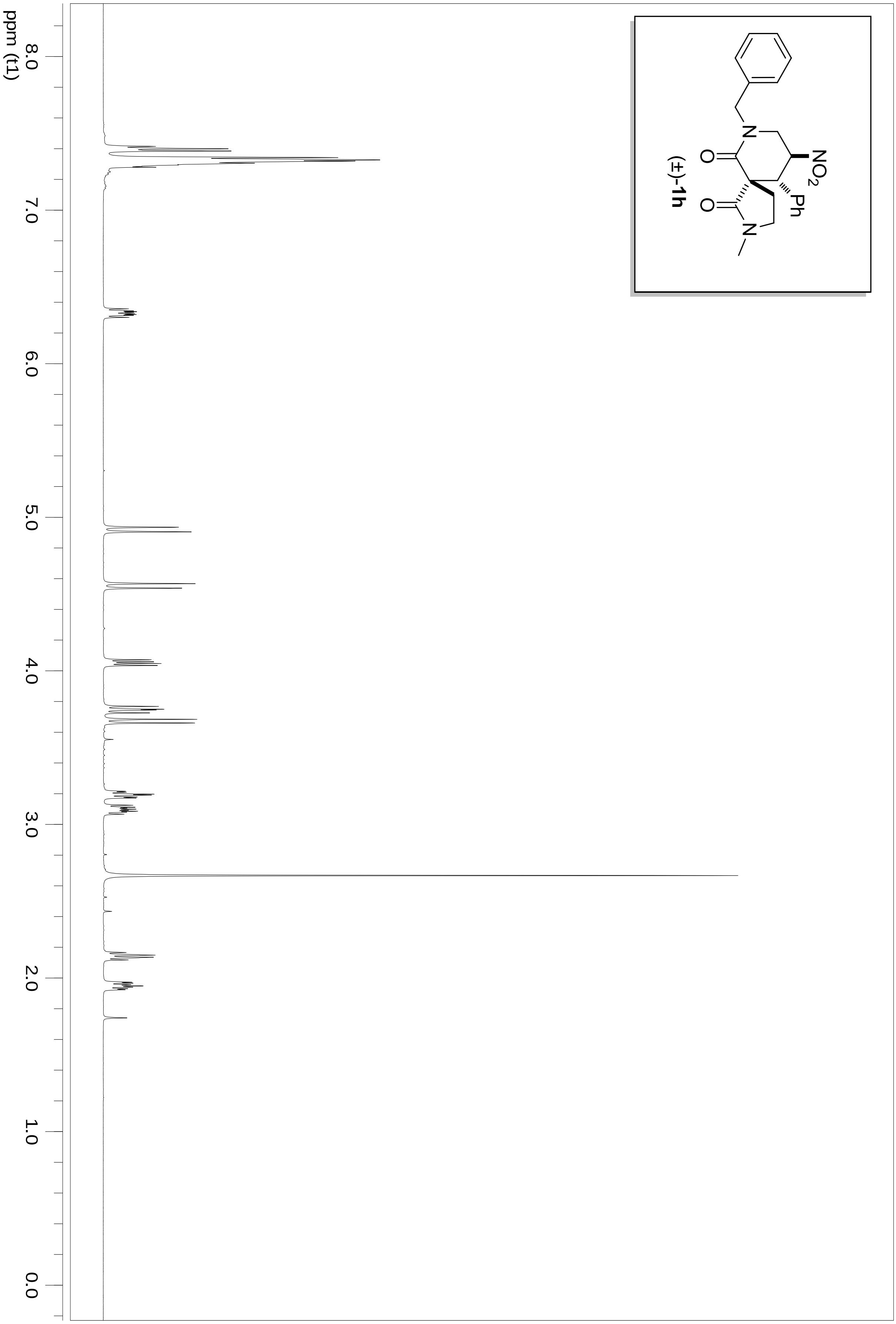

5.12. ^{13}C NMR spectra of 1h

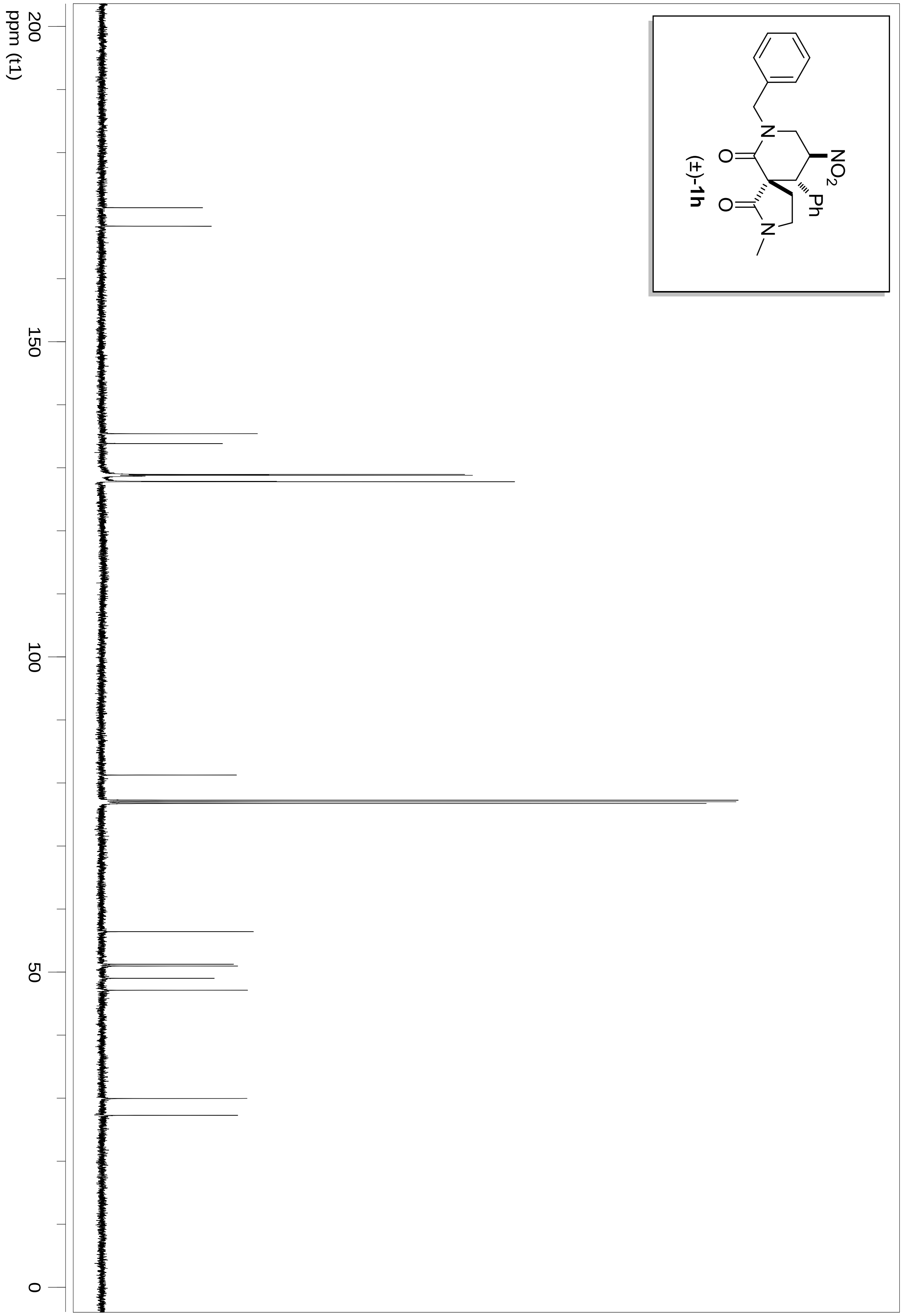

5.13. ^{1}H NMR spectra of 1i

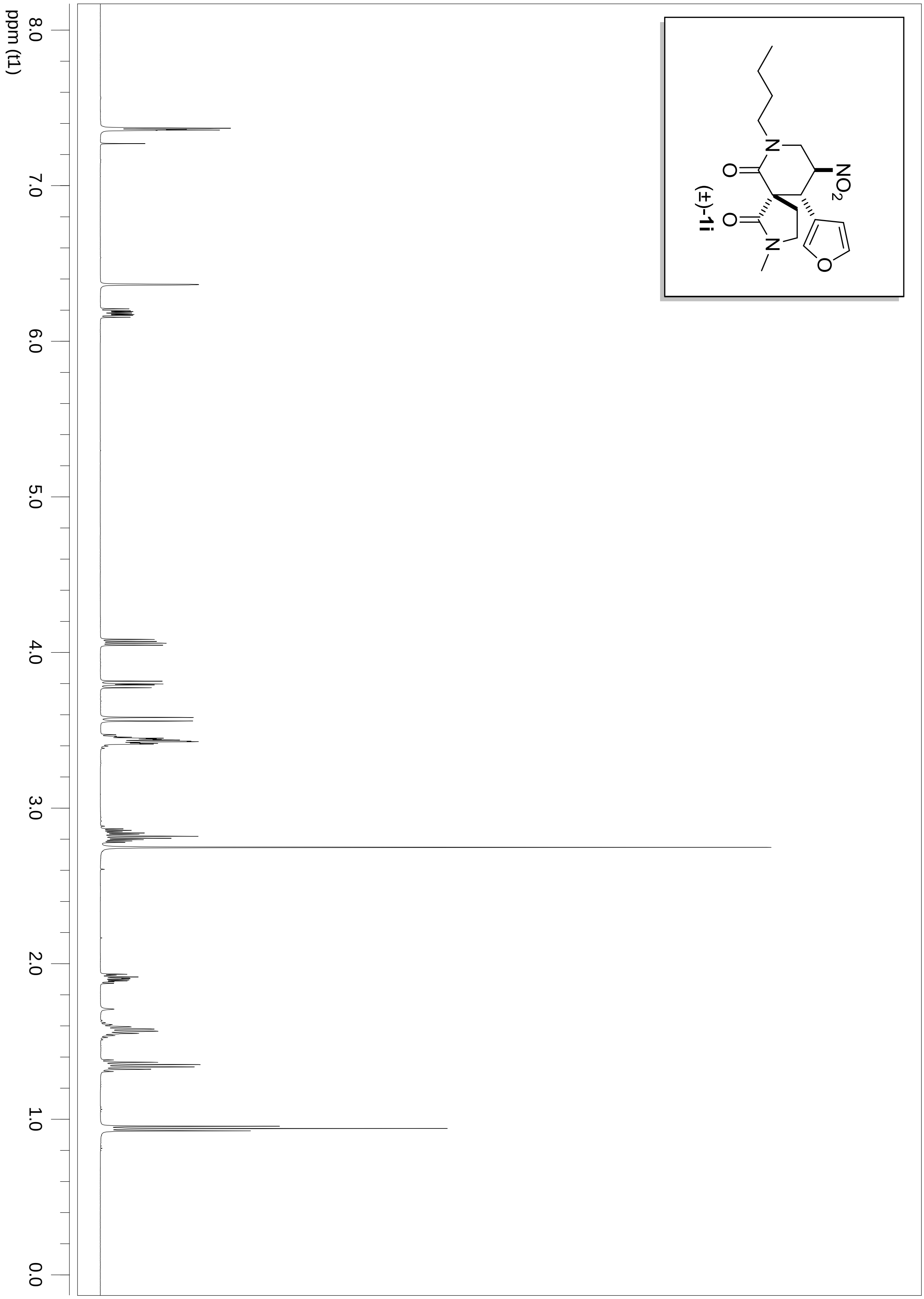

5.13. ^{13}C NMR of 1i

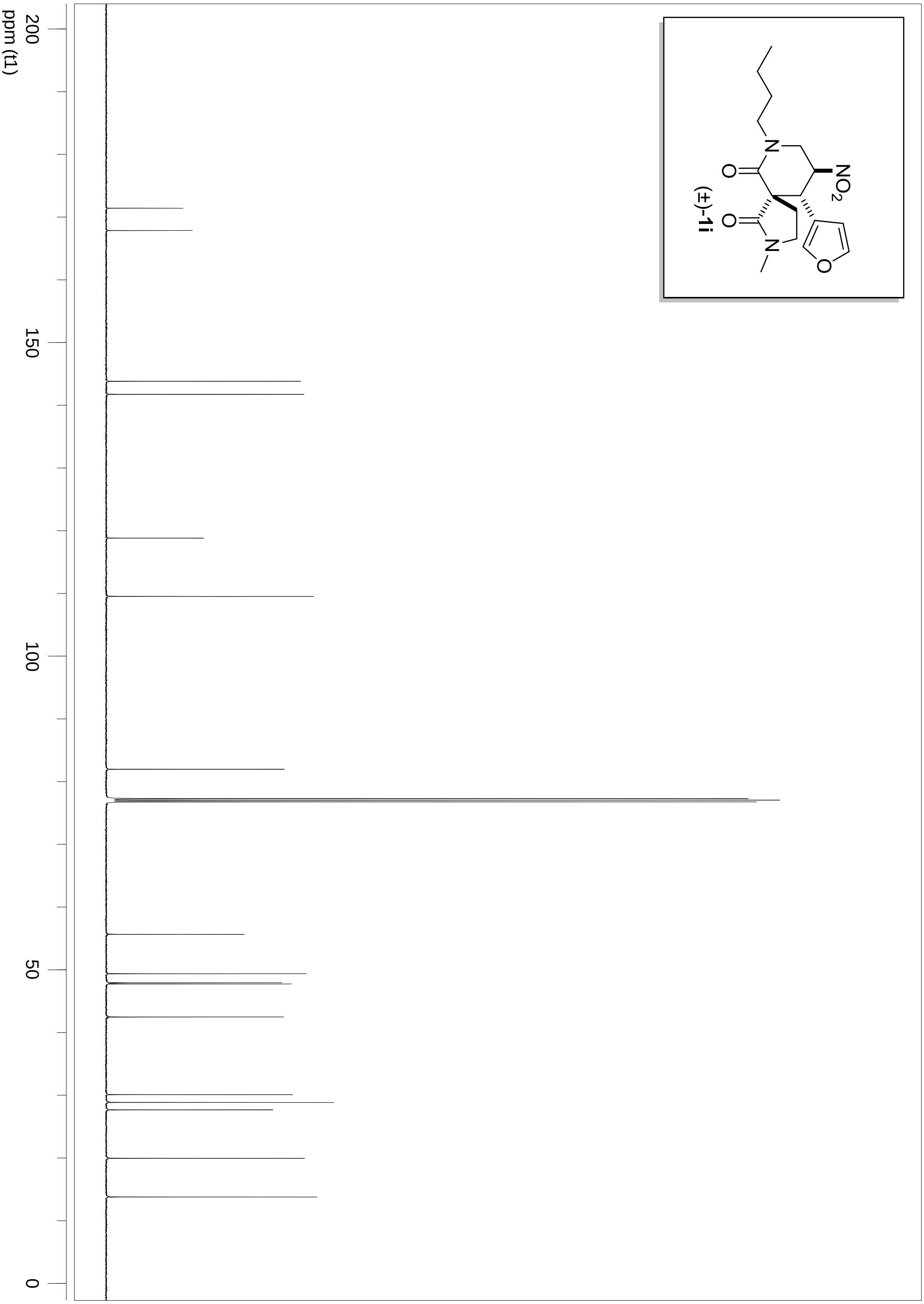

5.14. ^{1}H NMR spectra of 1j

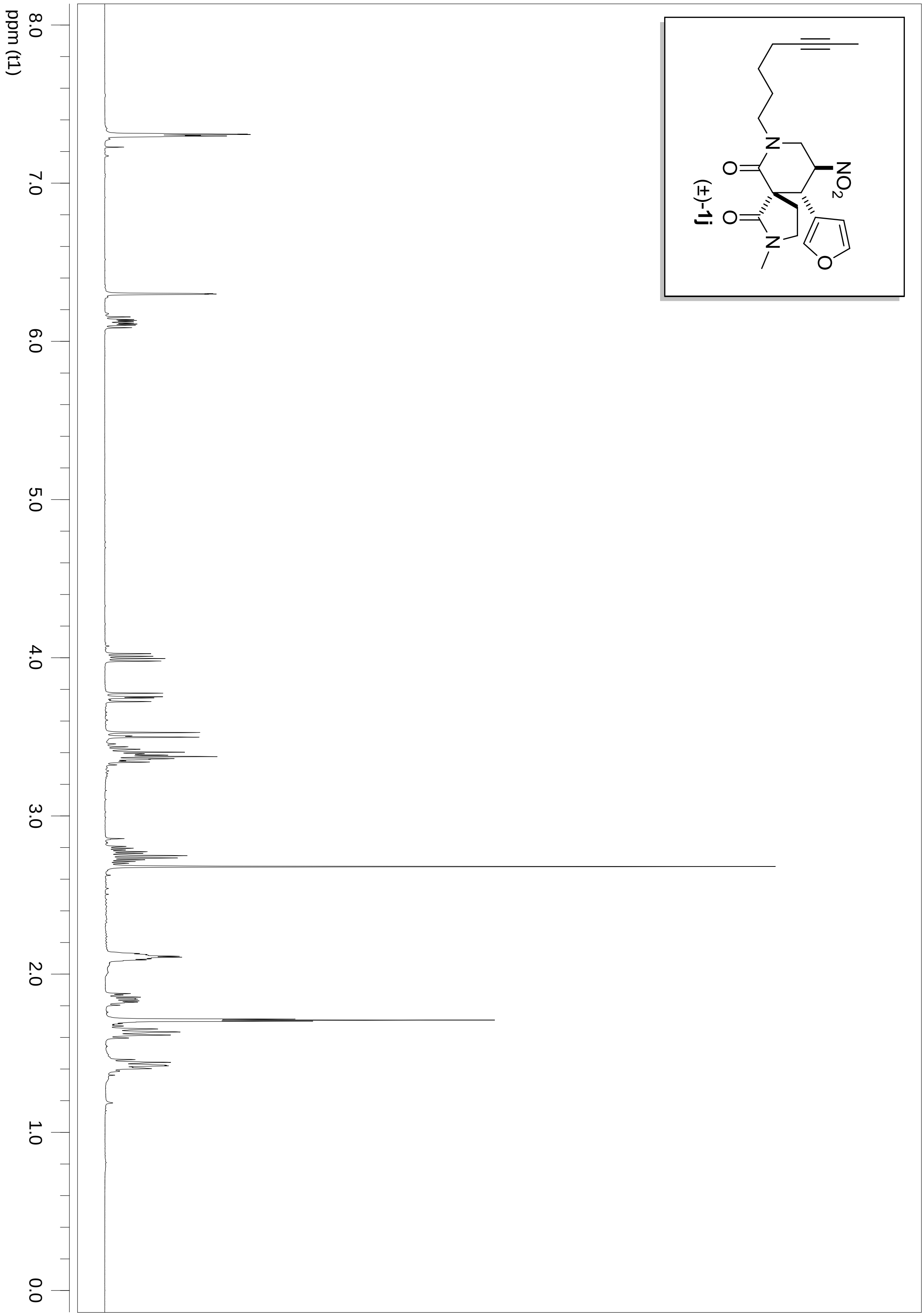

5.14. ^{13}C NMR spectra of 1j

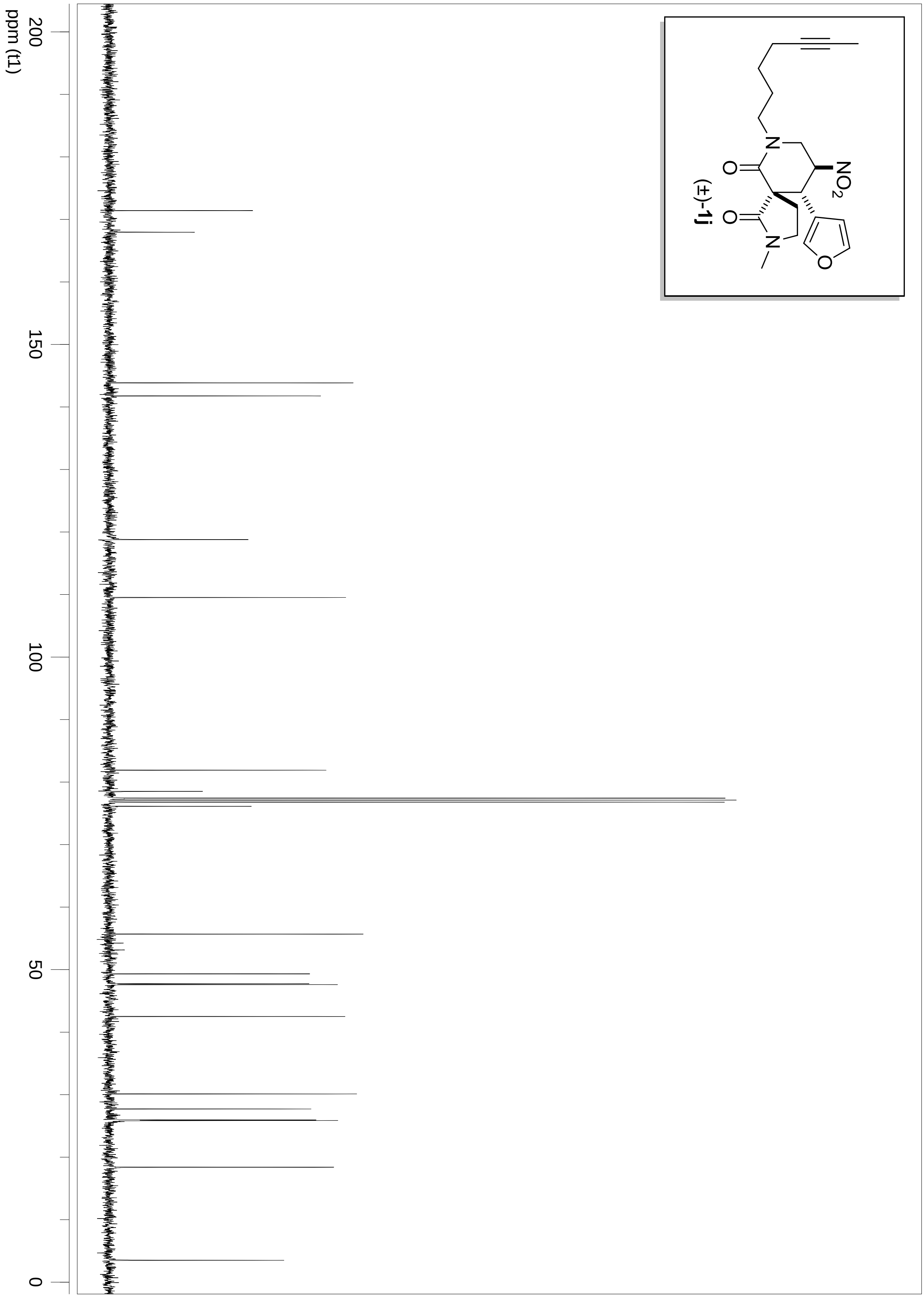

5.15. ^{1}H NMR spectra of 2a

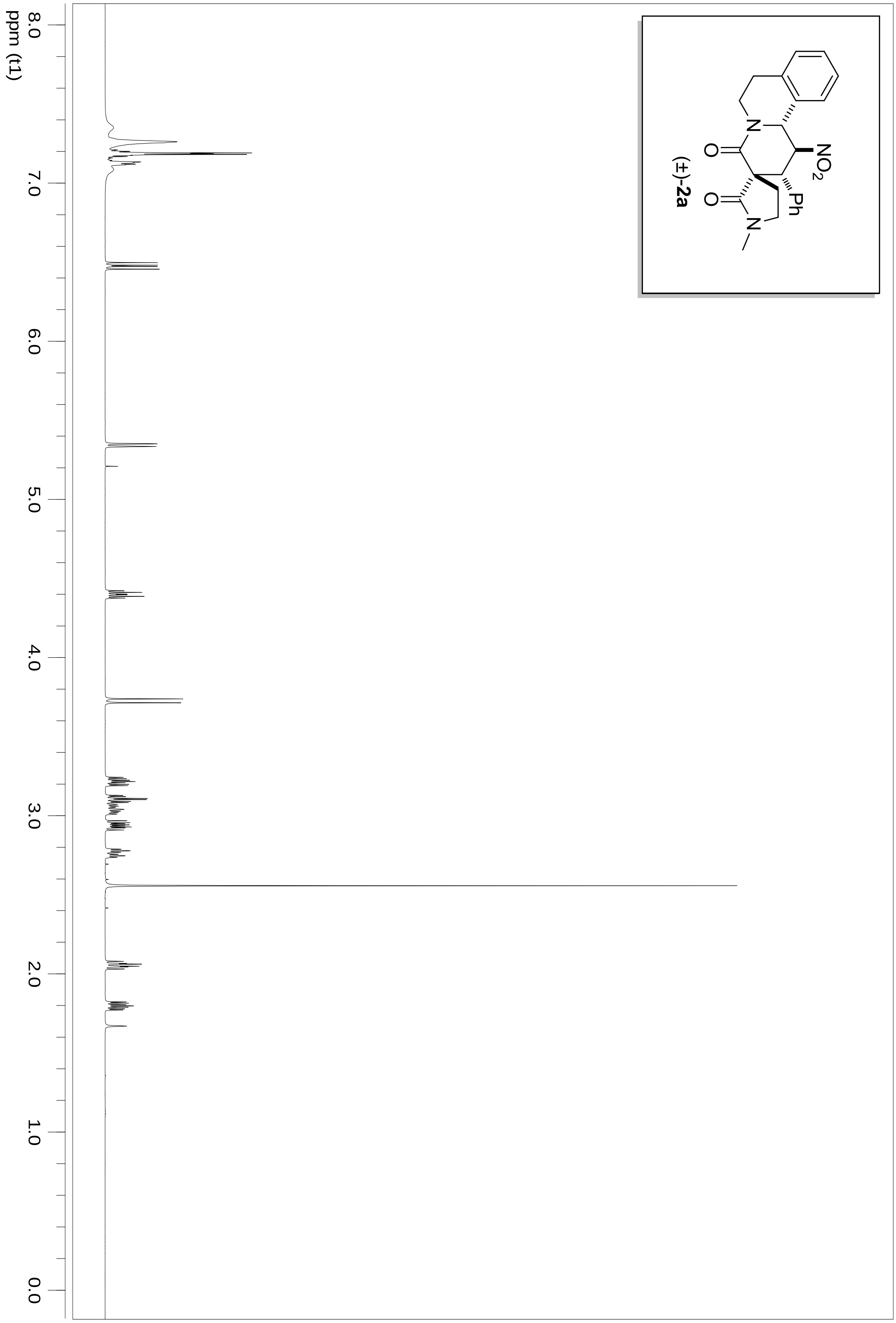

5.15. ^{13}C NMR spectra of 2a

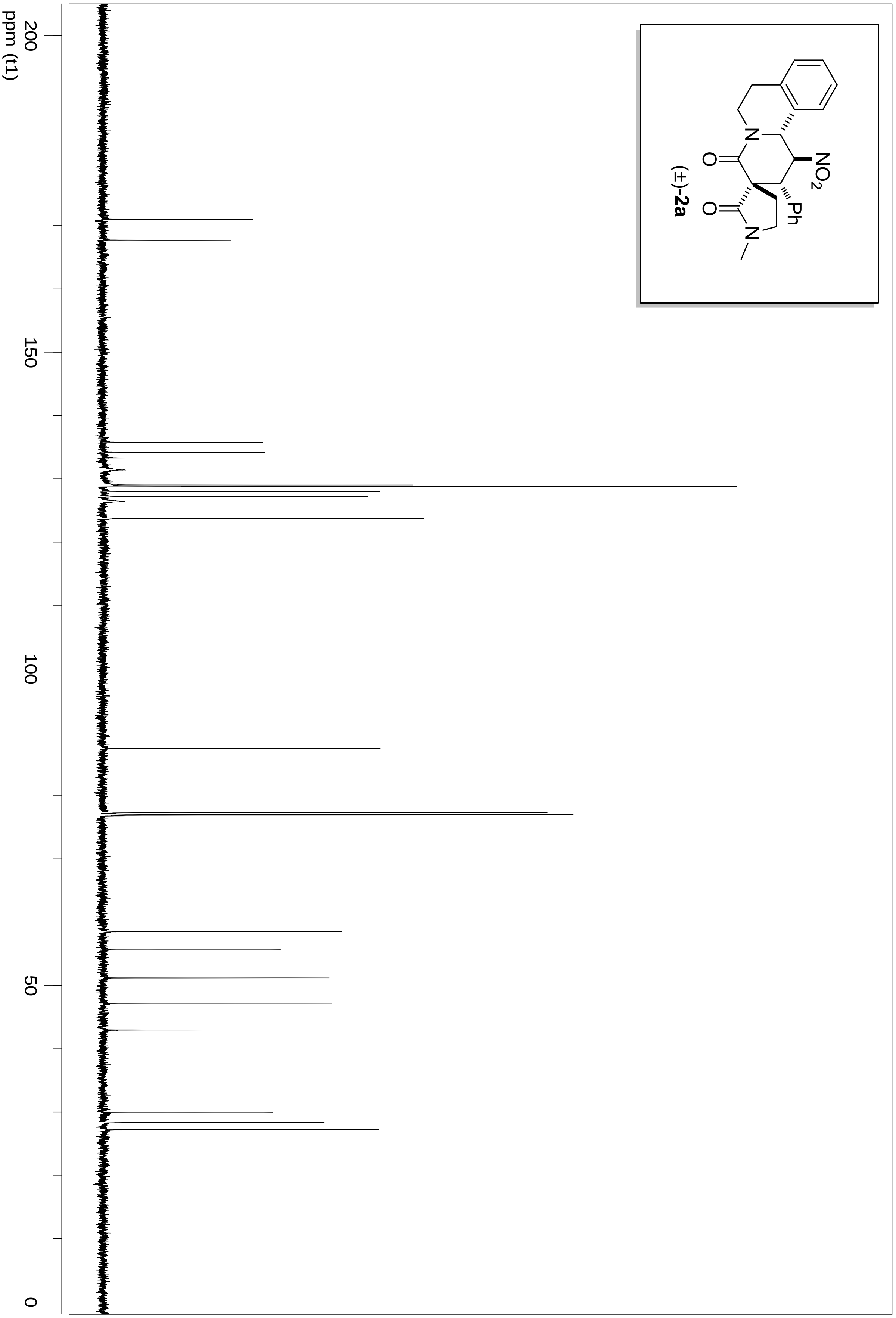

5.16. ^{1}H NMR spectra of 2a''

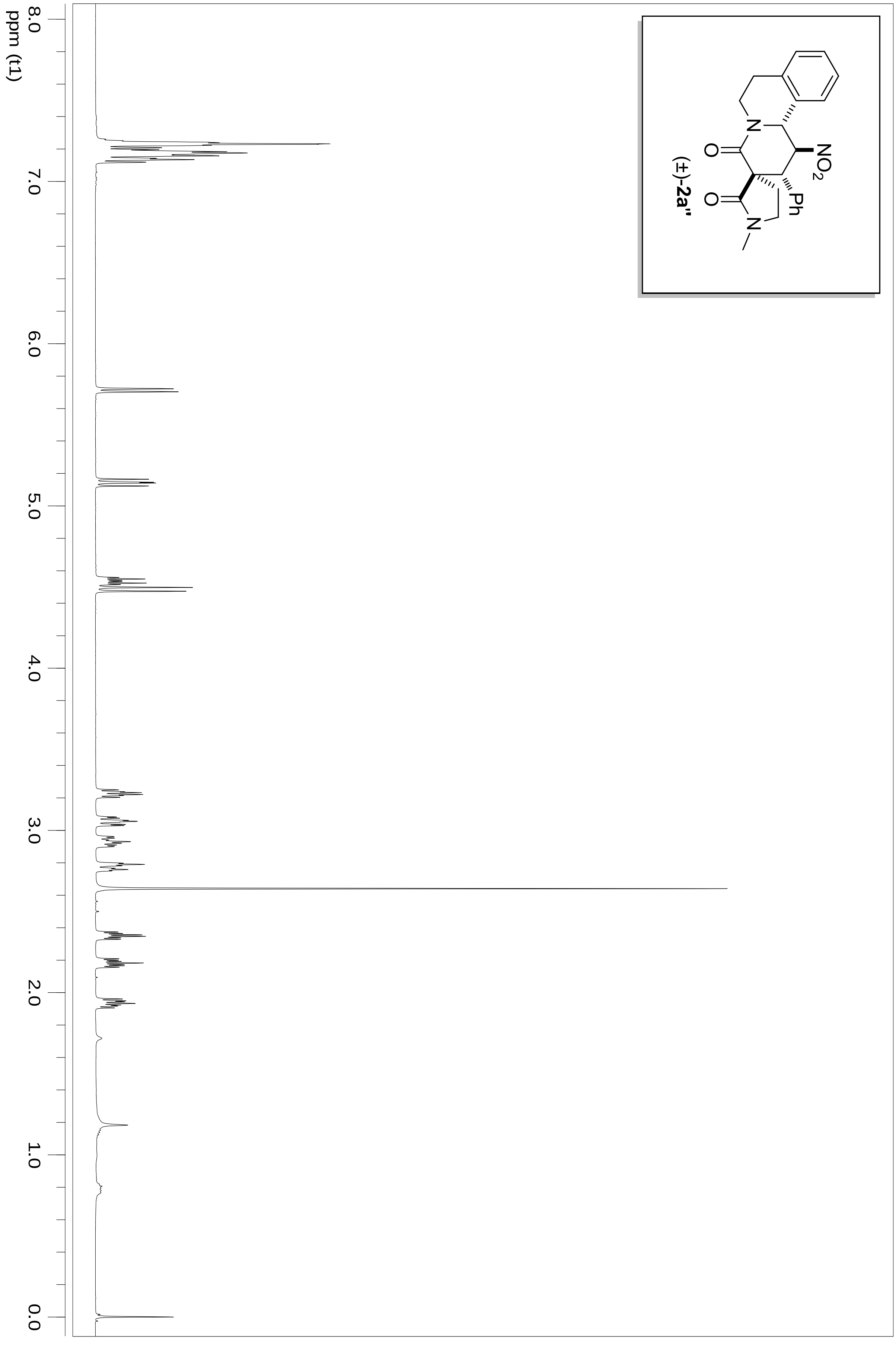

5.16. ^{13}C NMR spectra of 2a''

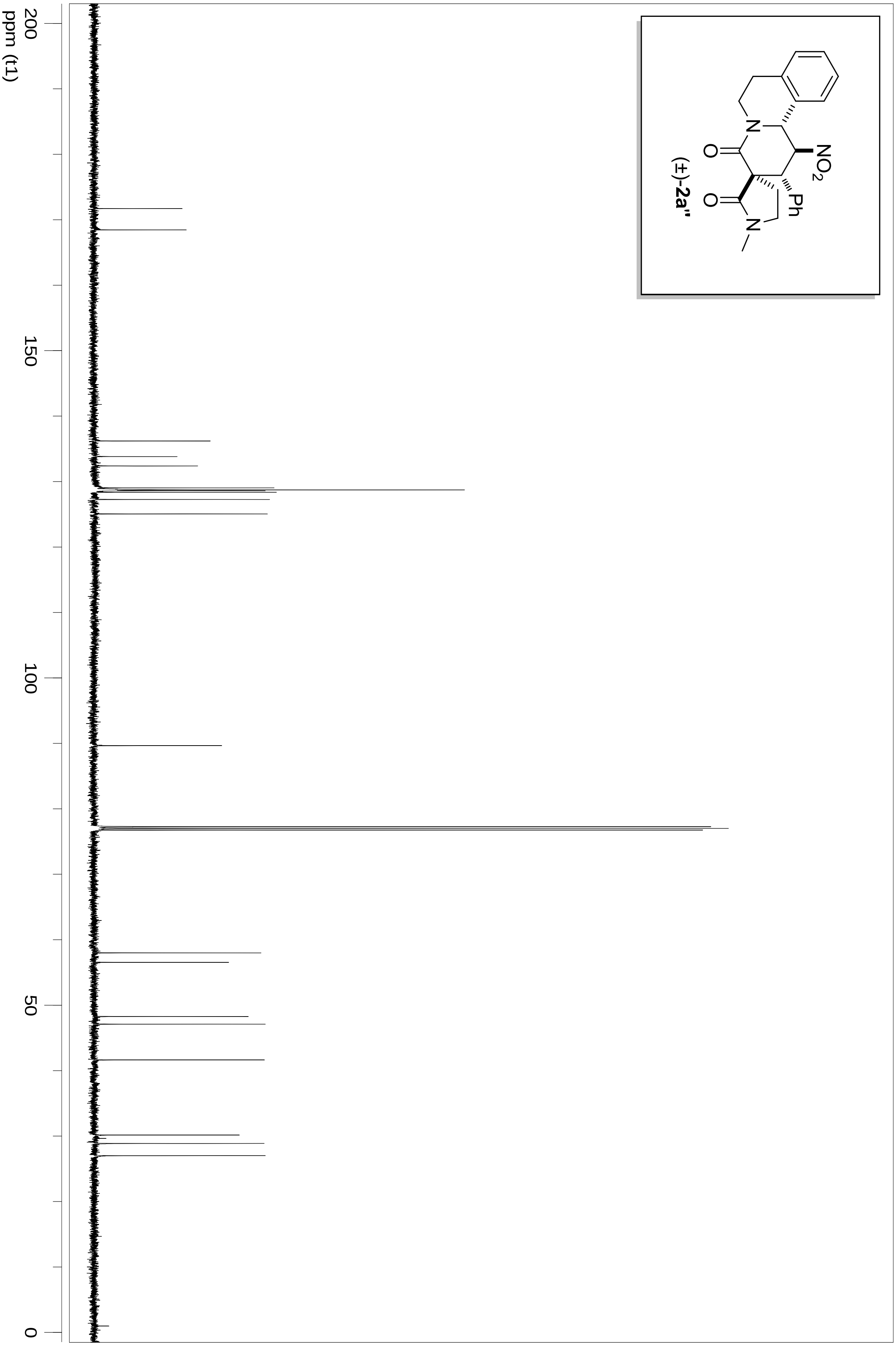

5.17. ^{1}H NMR spectra of 2m

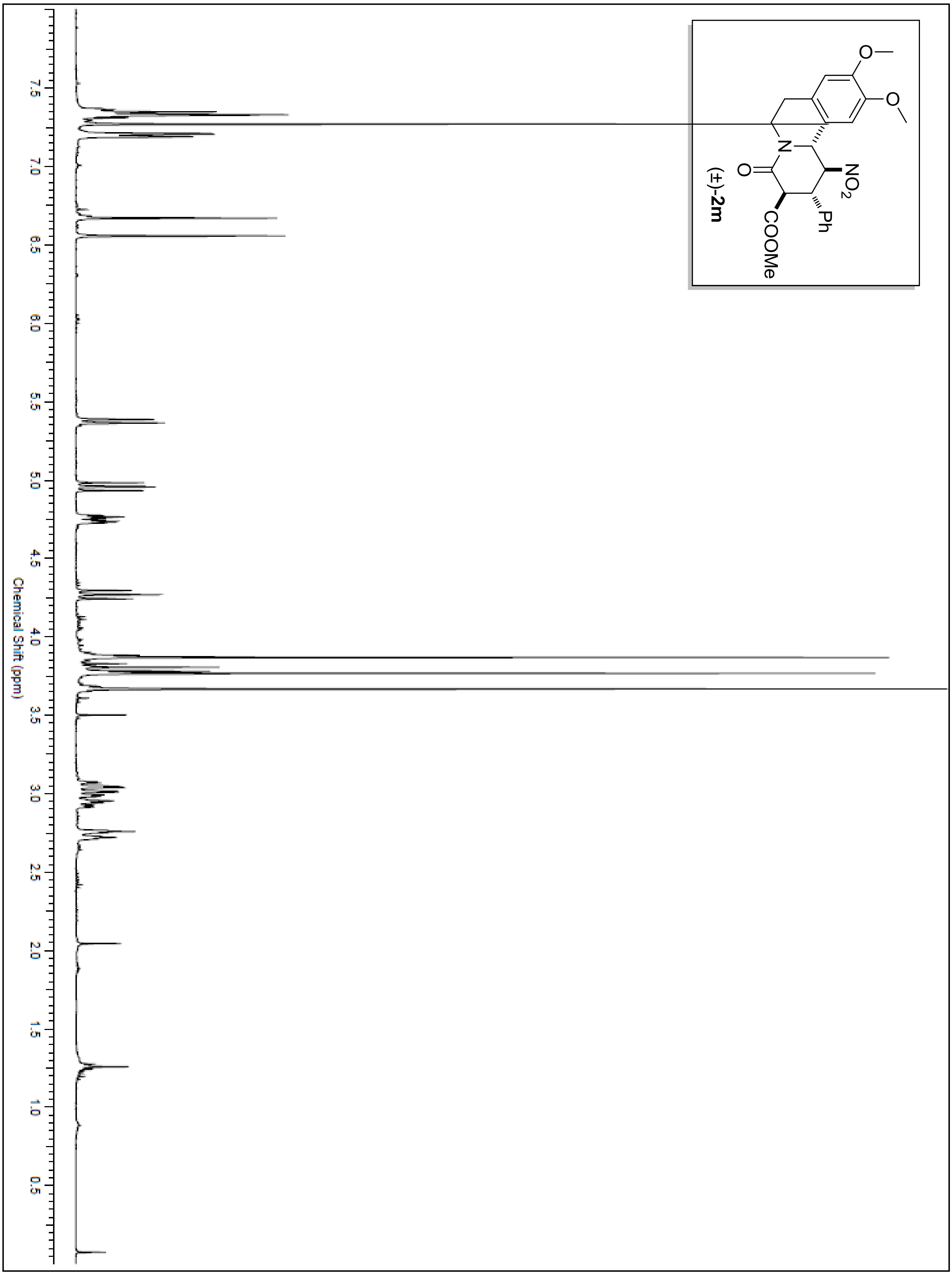

5.17. ^{13}C NMR spectra of 2m

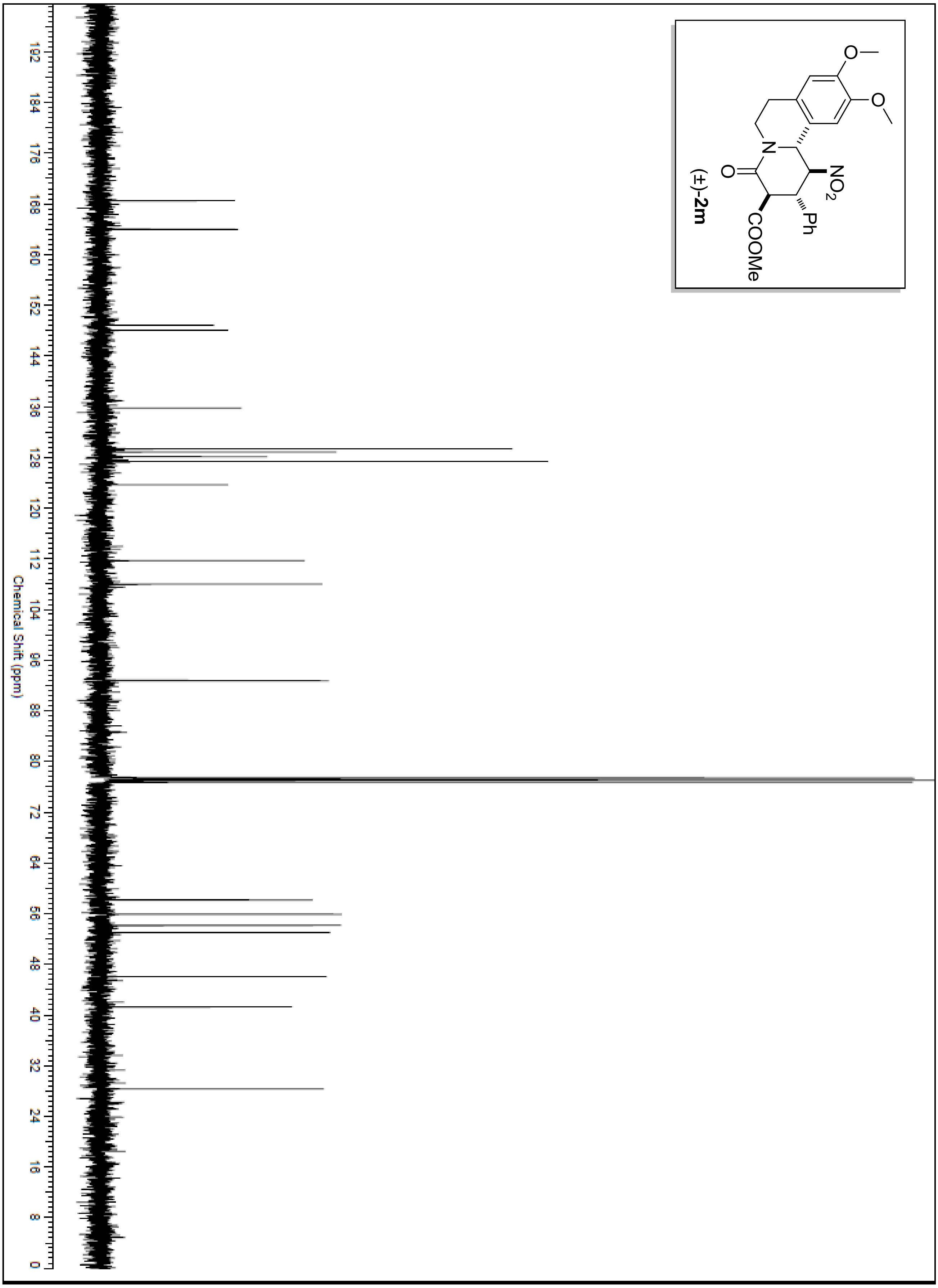

5.18. ^{1}H NMR spectra of 2m’

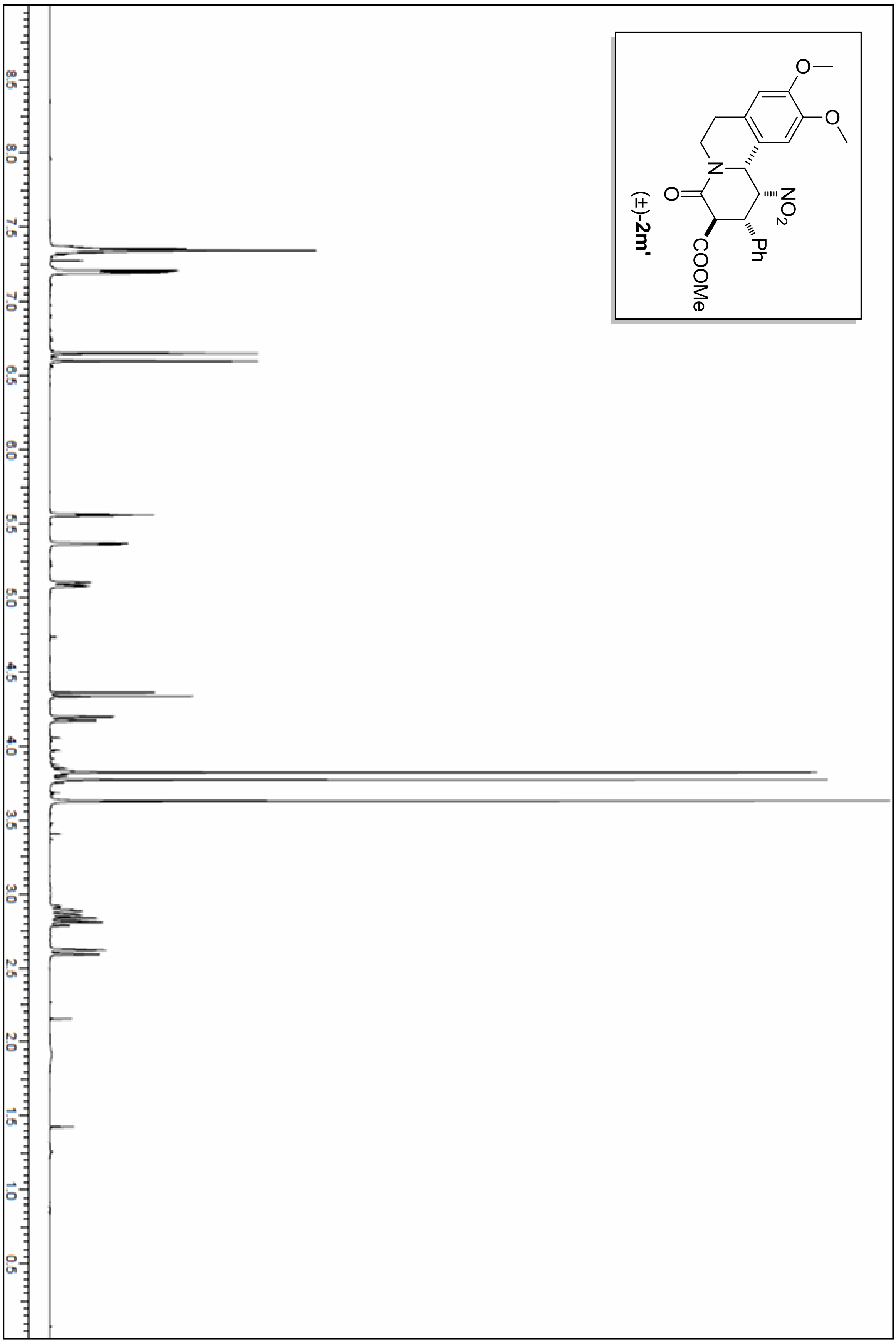

5.18. ^{1}H NMR spectra of 2m'

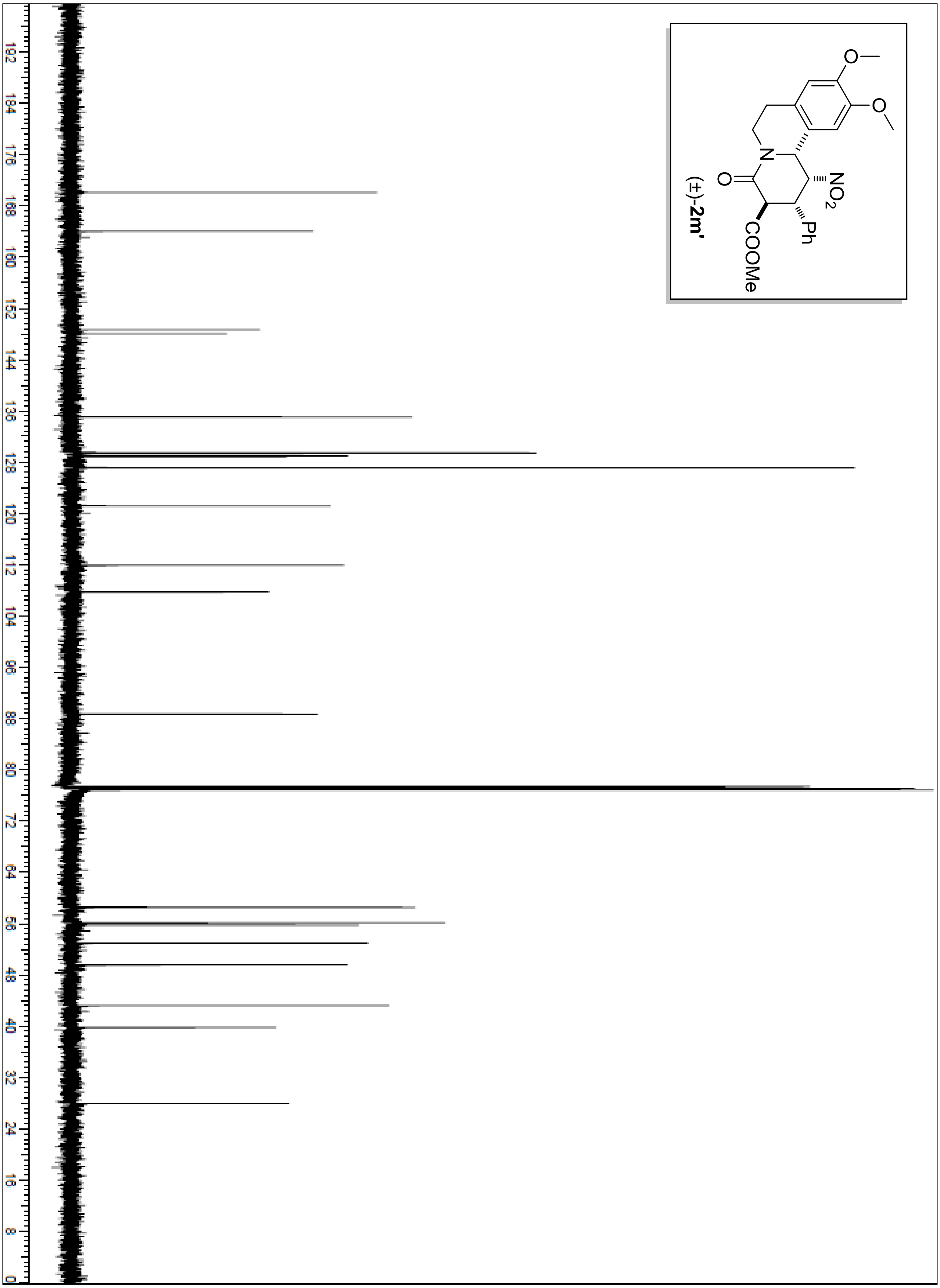

5.19. ^{1}H NMR spectra of 10a

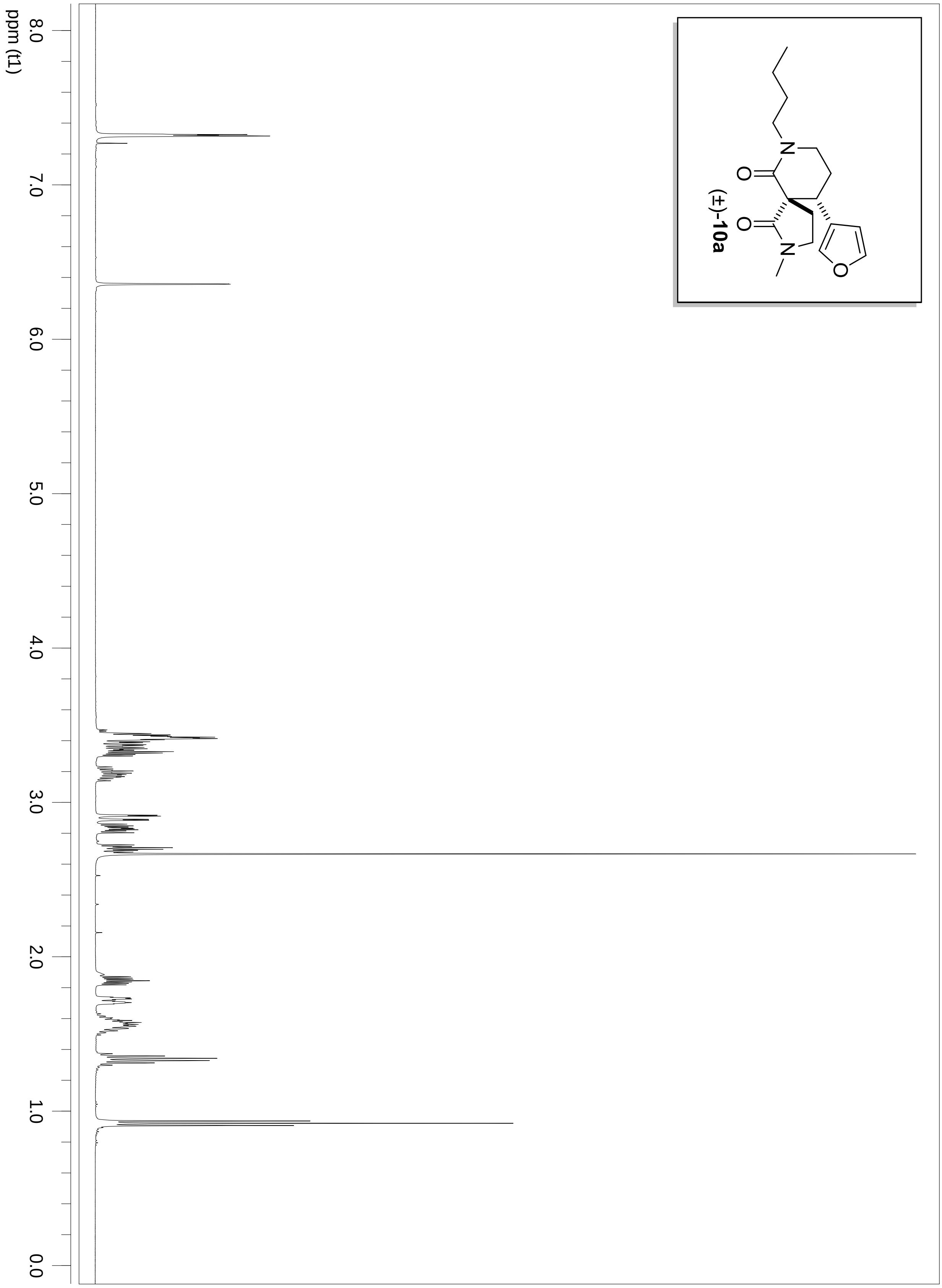

5.19 ^{13}C NMR spectra of 10a

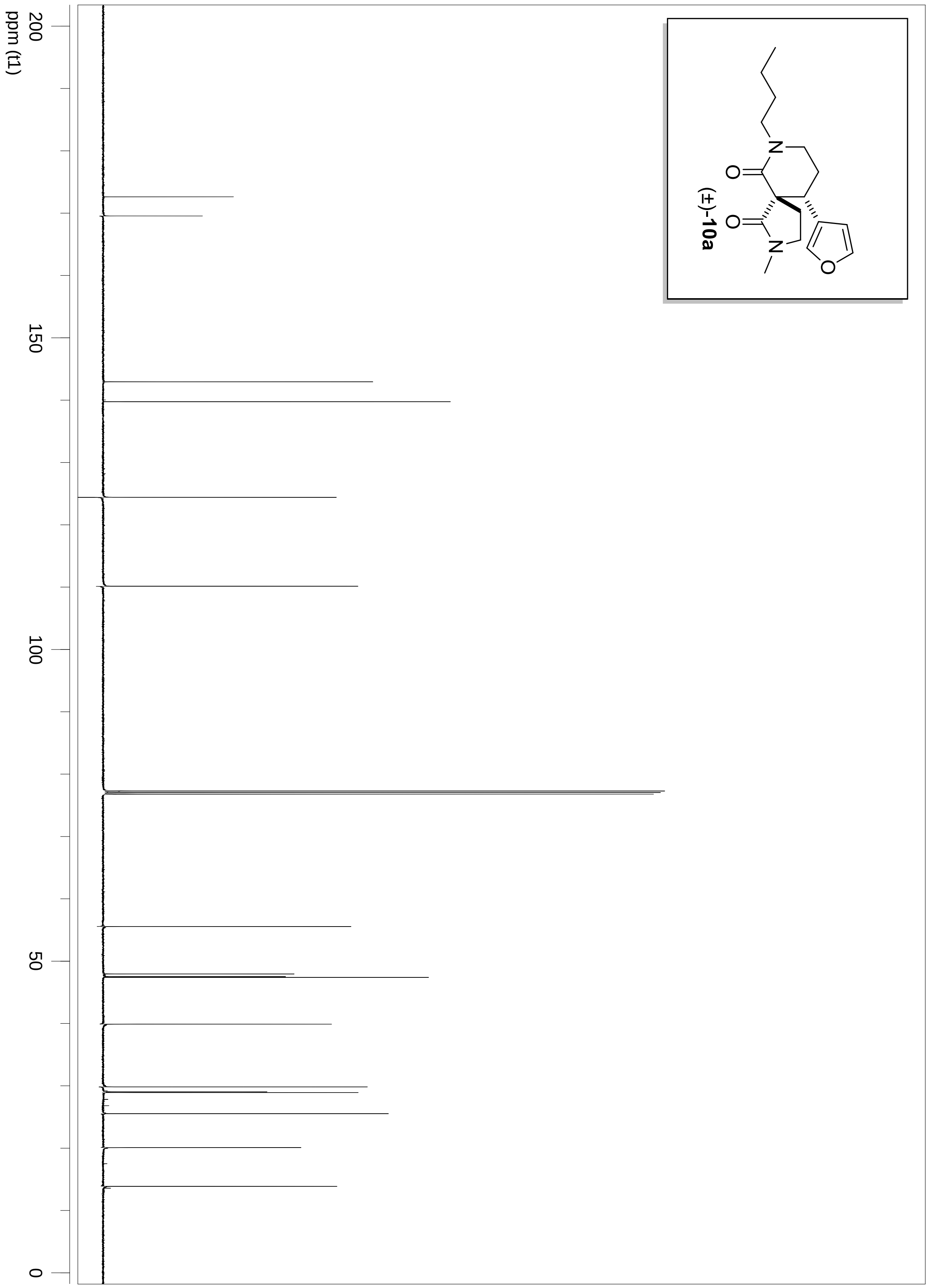

5.20. ^{1}H NMR spectra of 10b

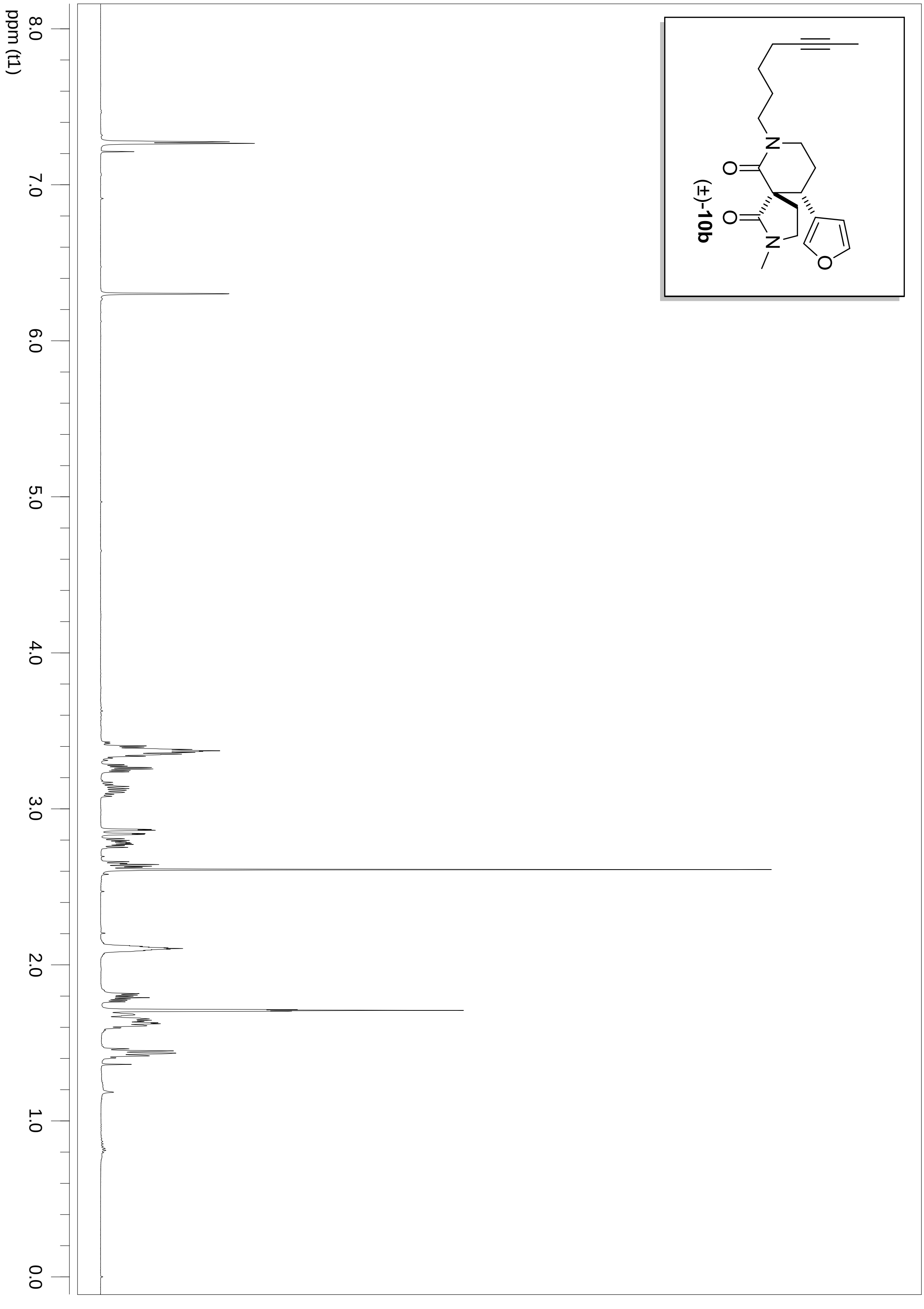

5.20. ^{13}C NMR spectra of 10b

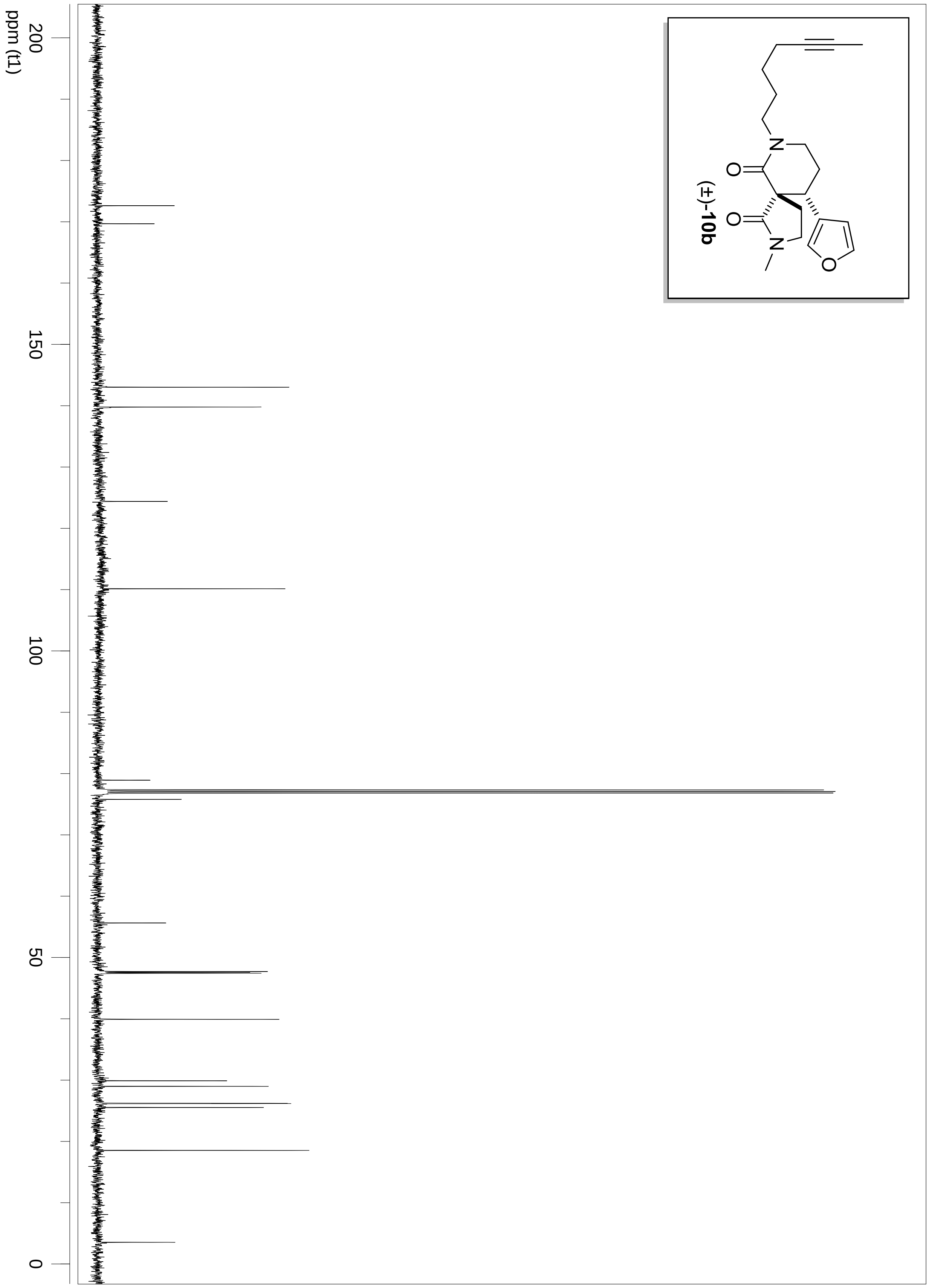

5.21. ^{1}H NMR spectra of 10c

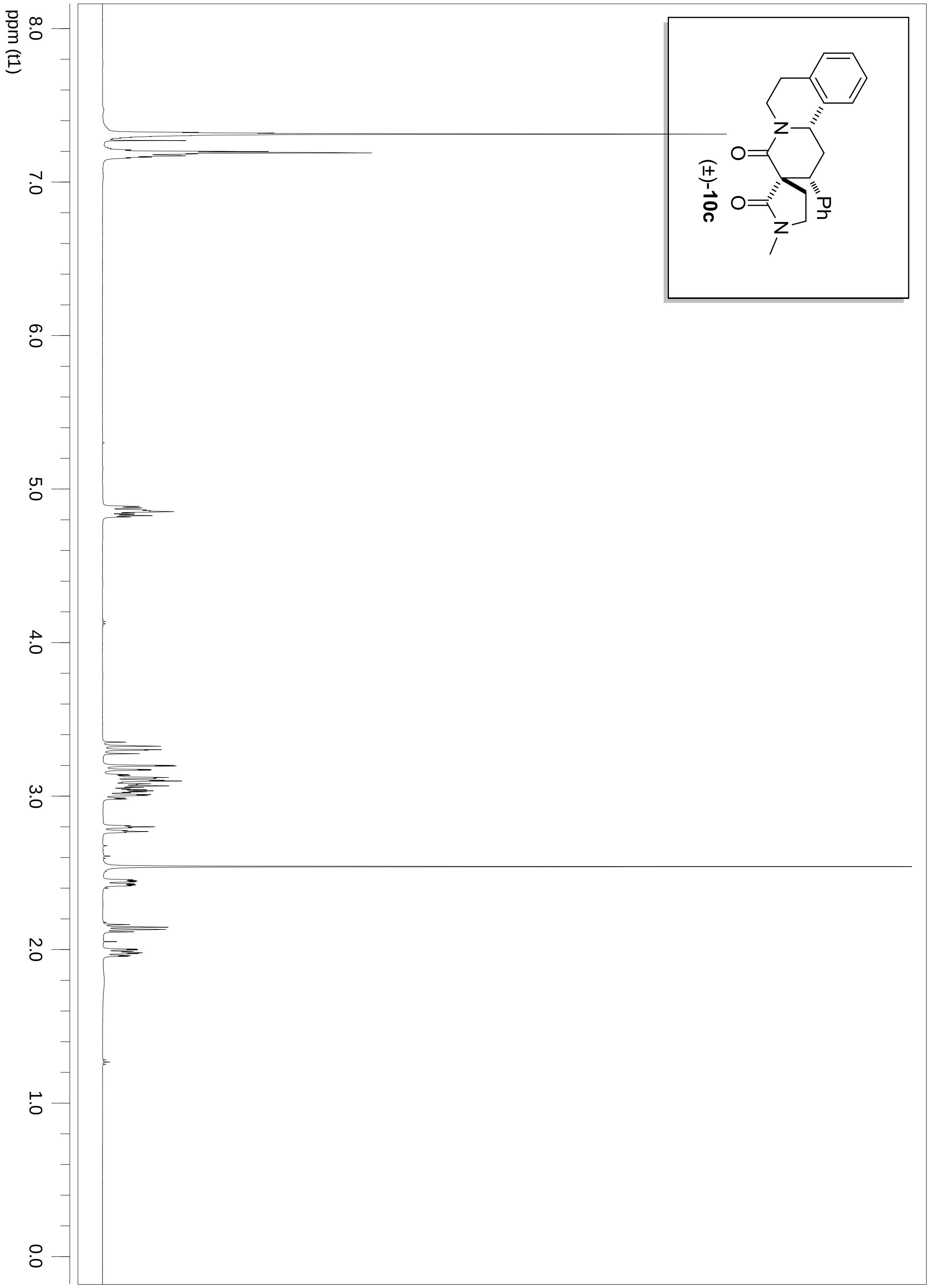

5.21. ^{13}C NMR spectra of 10c

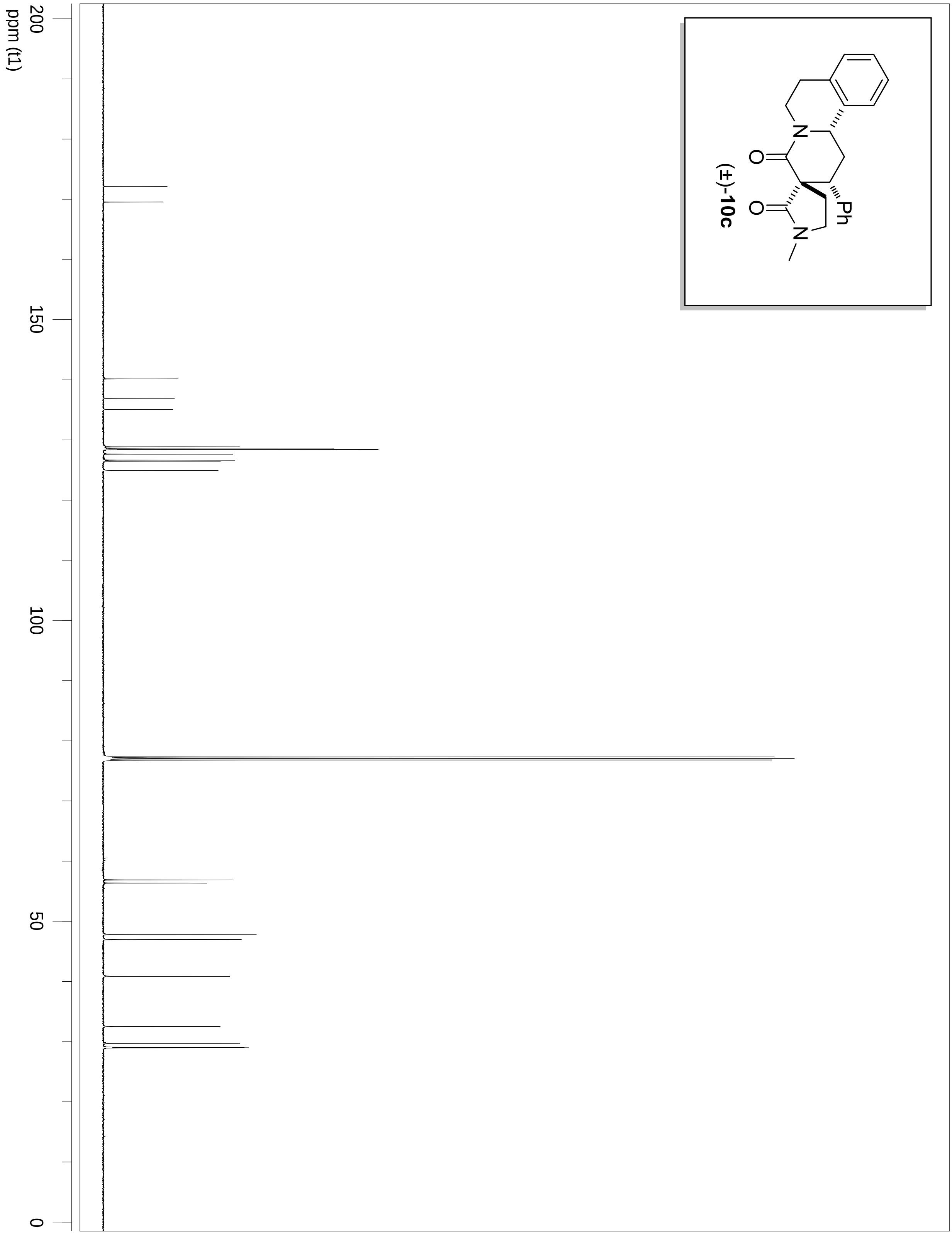

5.22. ^{1}H NMR spectra of 10d

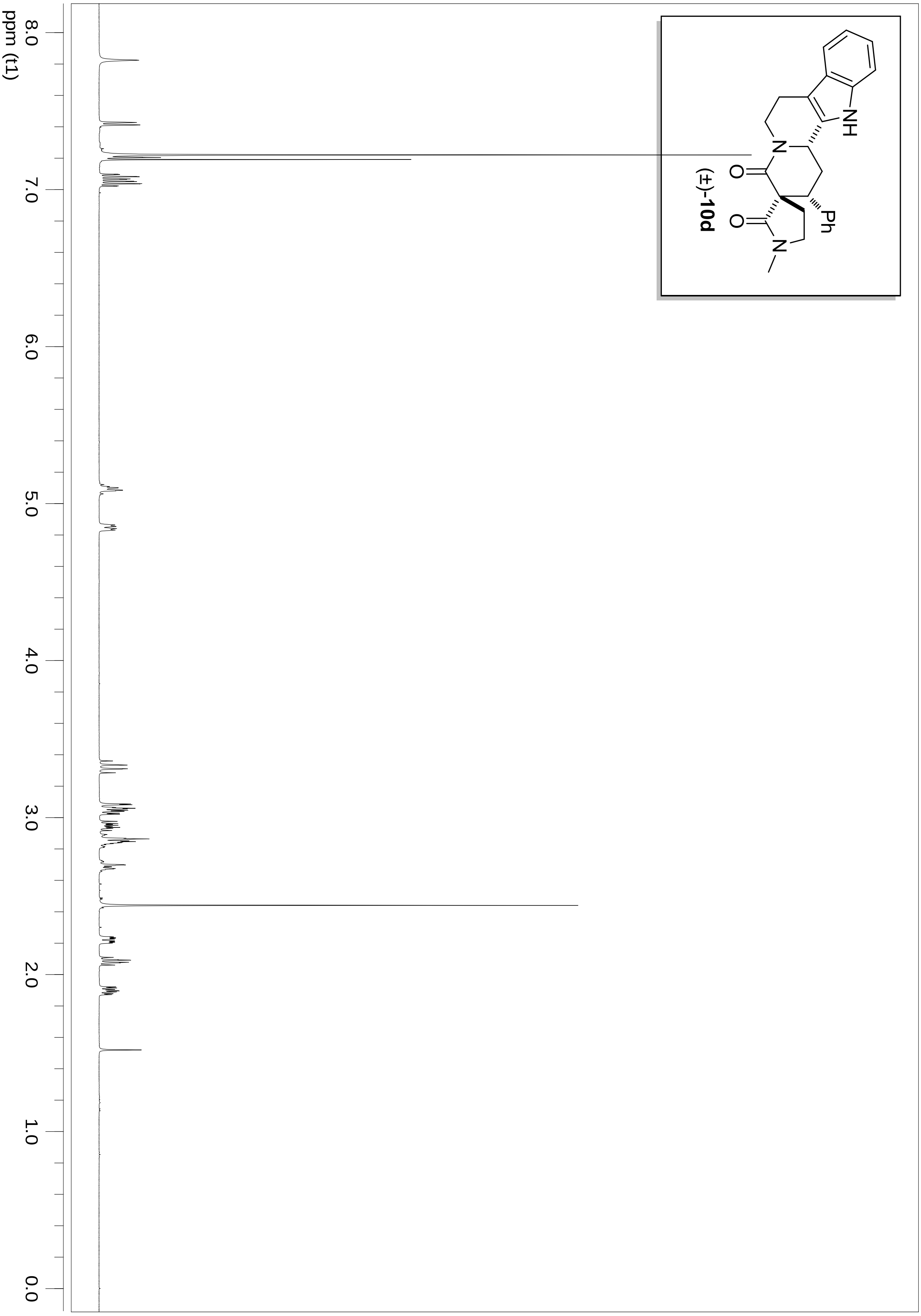

5.22. ^{13}C NMR spectra of 10d

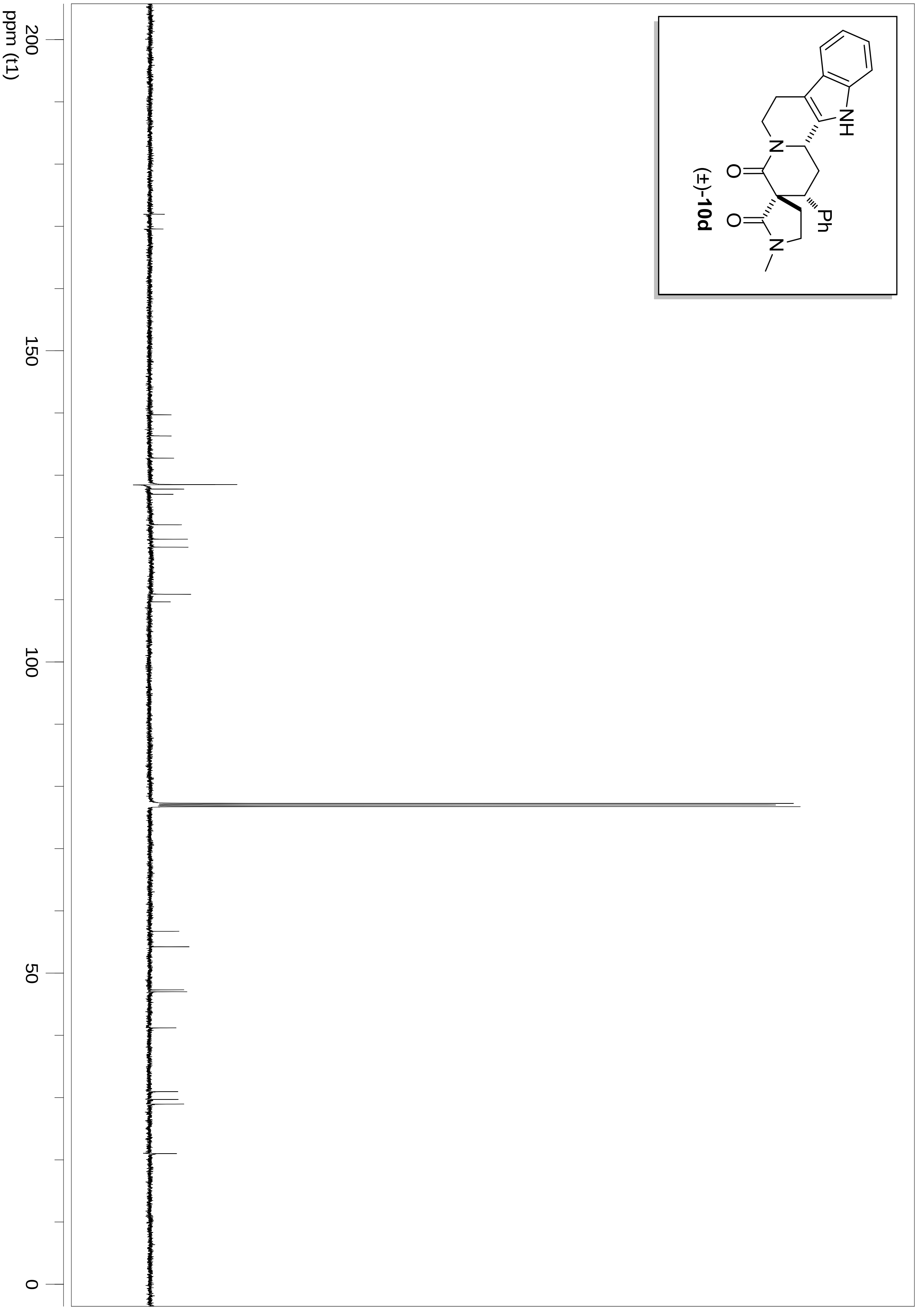

5.23. ^{1}H NMR spectra of 11a

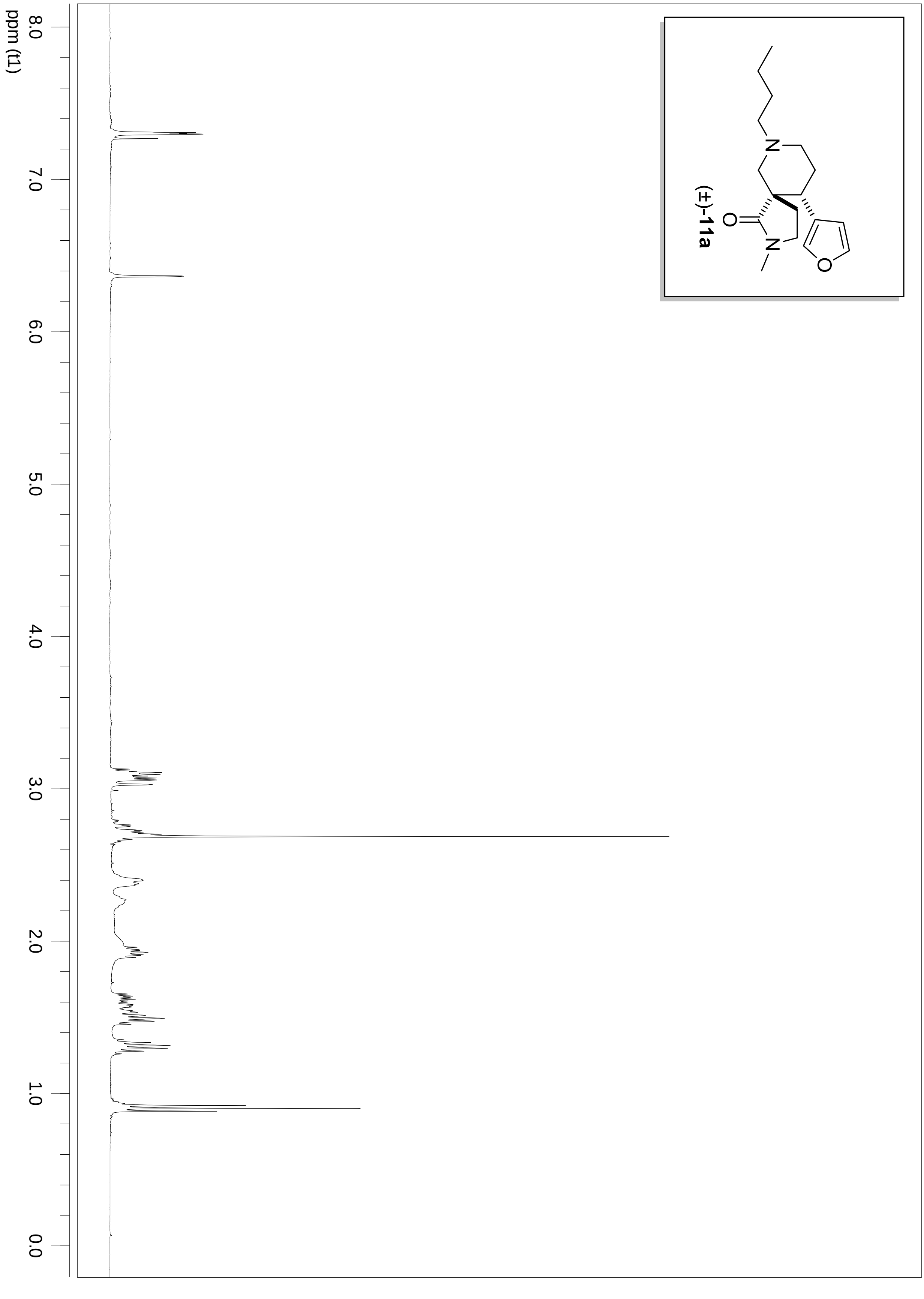

5.23. ^{13}C NMR spectra of 11a

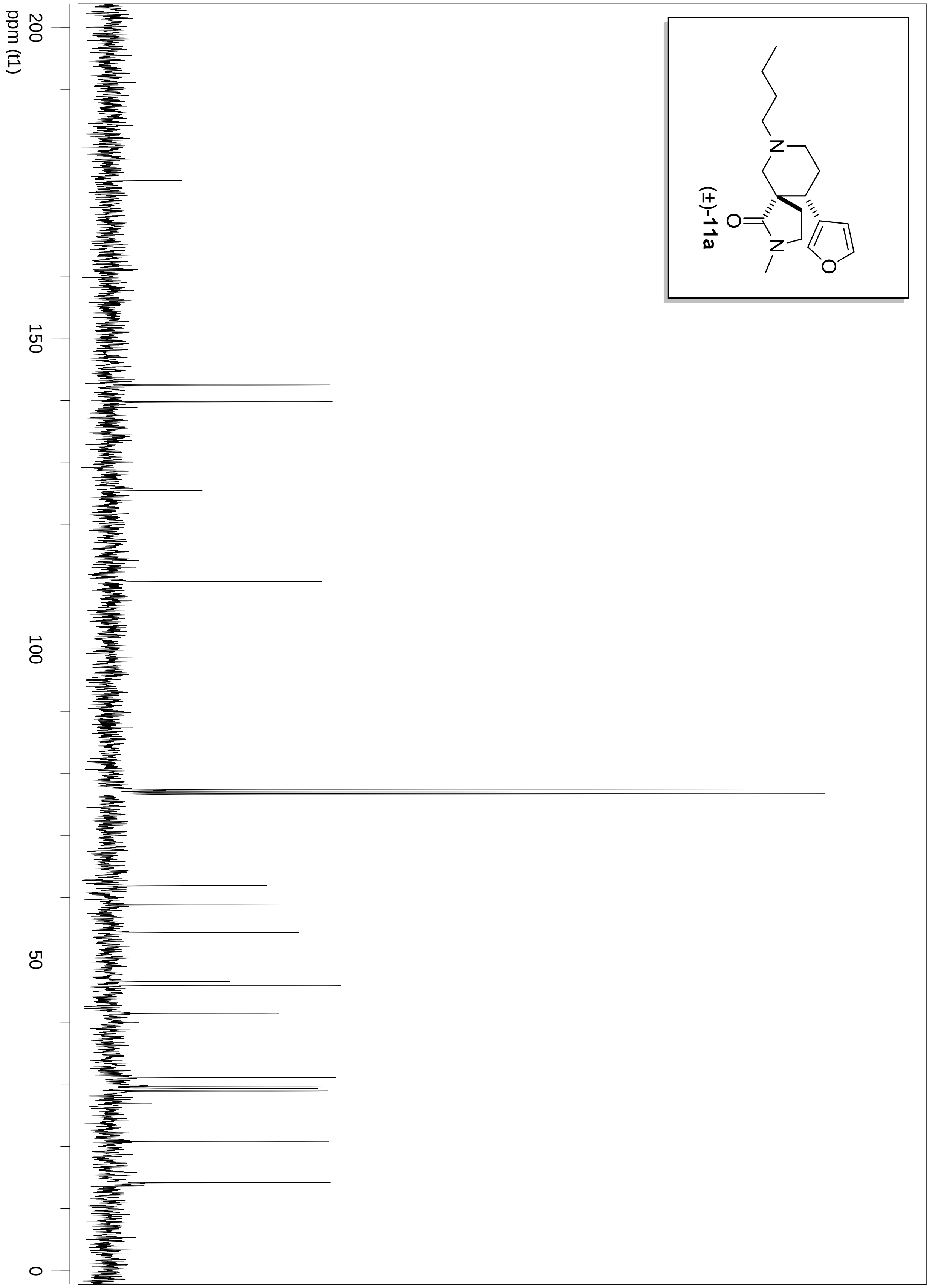

5.24. ^{1}H NMR spectra of 11b

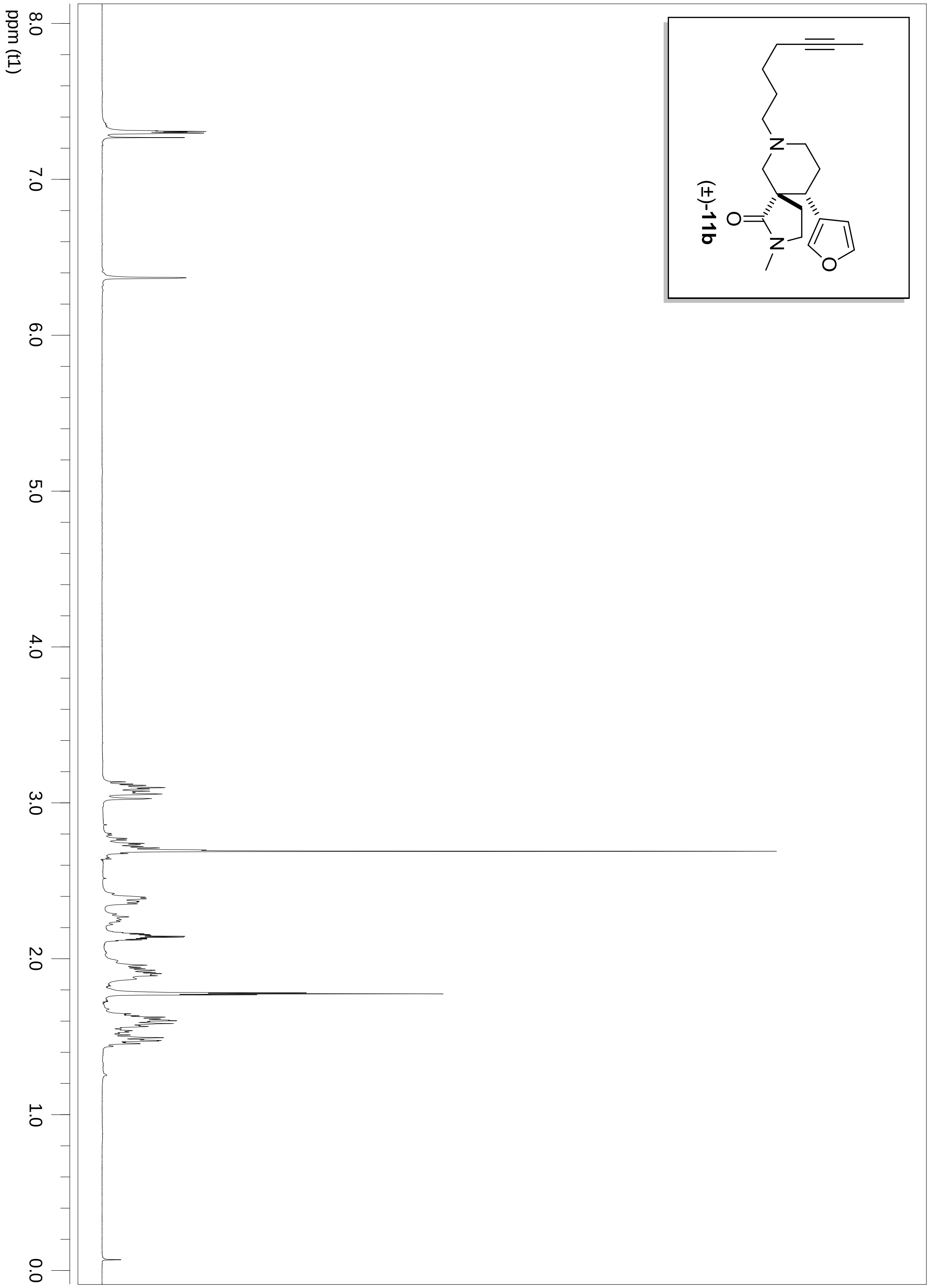

5.24. ^{13}C NMR spectra of 11b

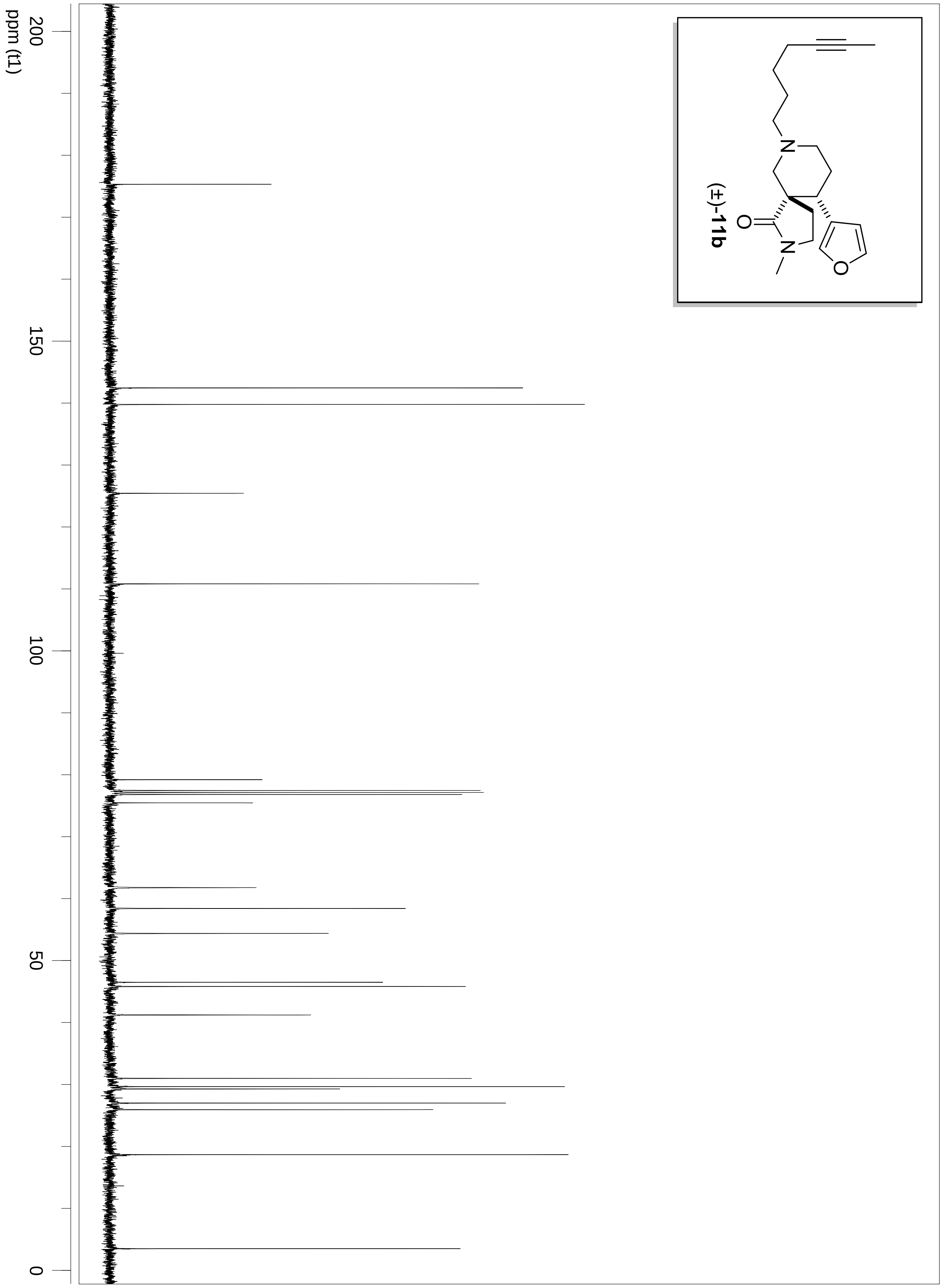

5.25. ^{1}H NMR spectra of 11c

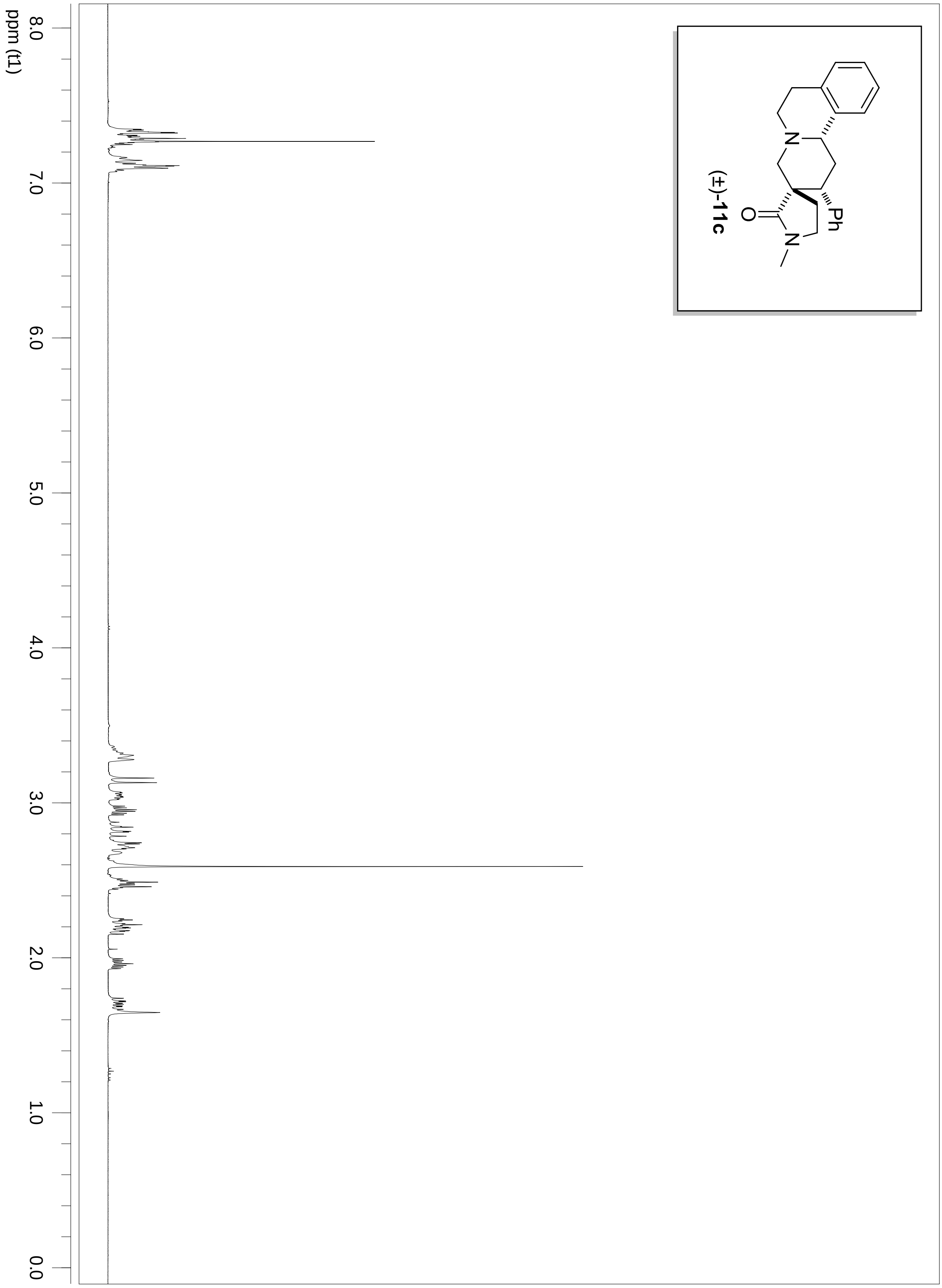

5.25. ^{13}C NMR spectra of 11c

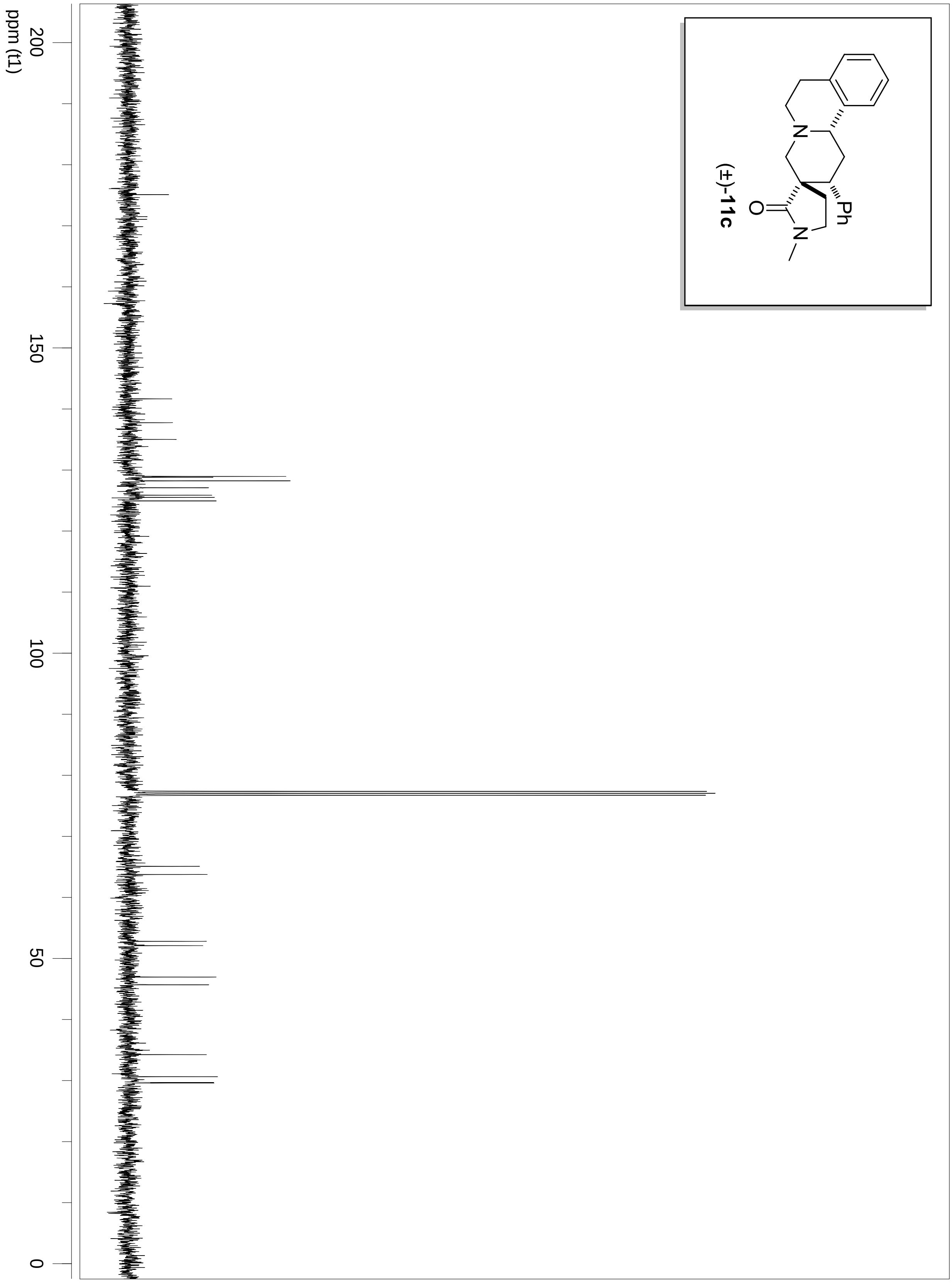

5.26. ^{1}H NMR spectra of 12a

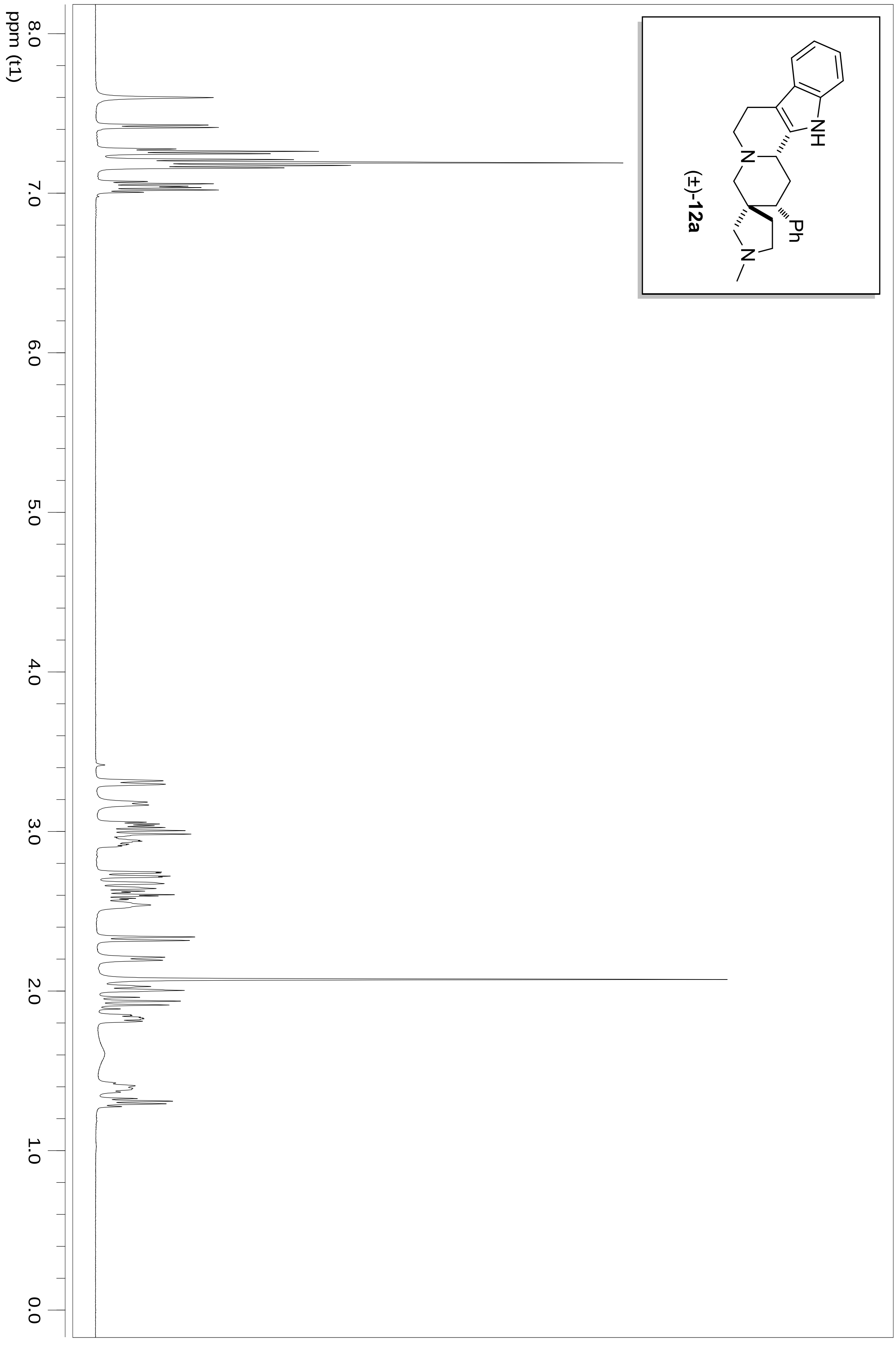

5.26. ^{13}C NMR spectra of 12a

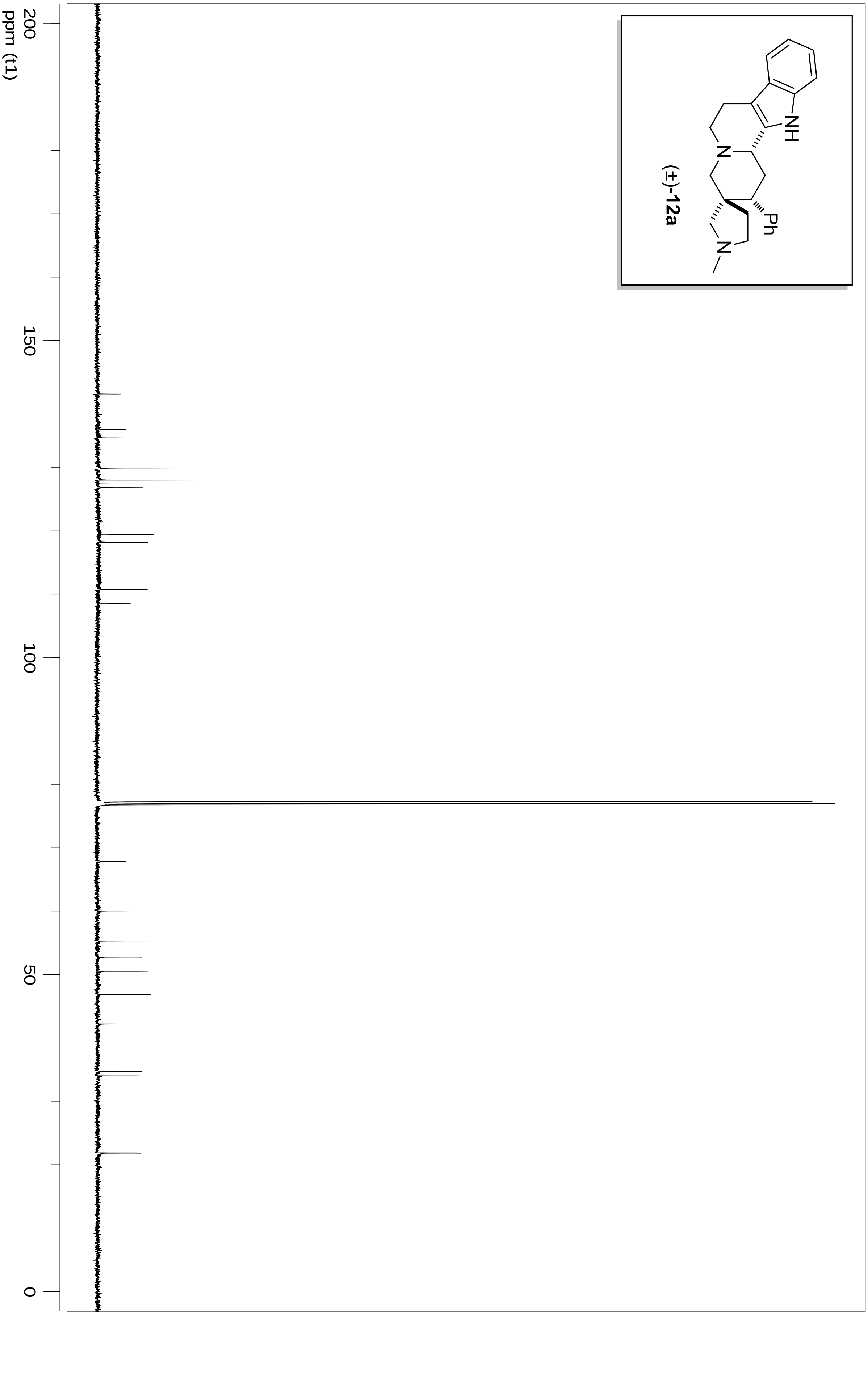

5.27 ^{1}H NMR spectra of 12b

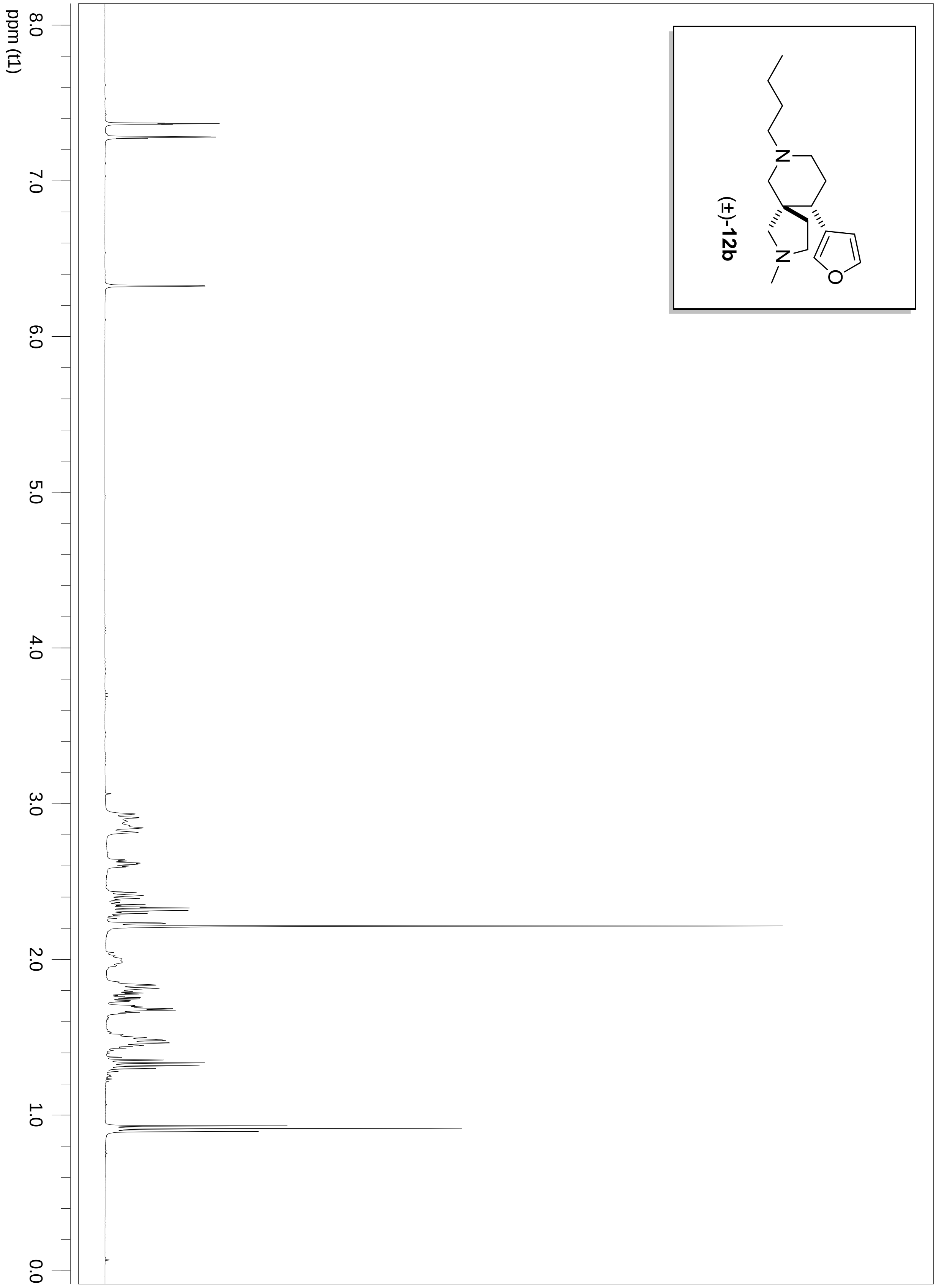

5.27. ^{13}C NMR spectra of 12b

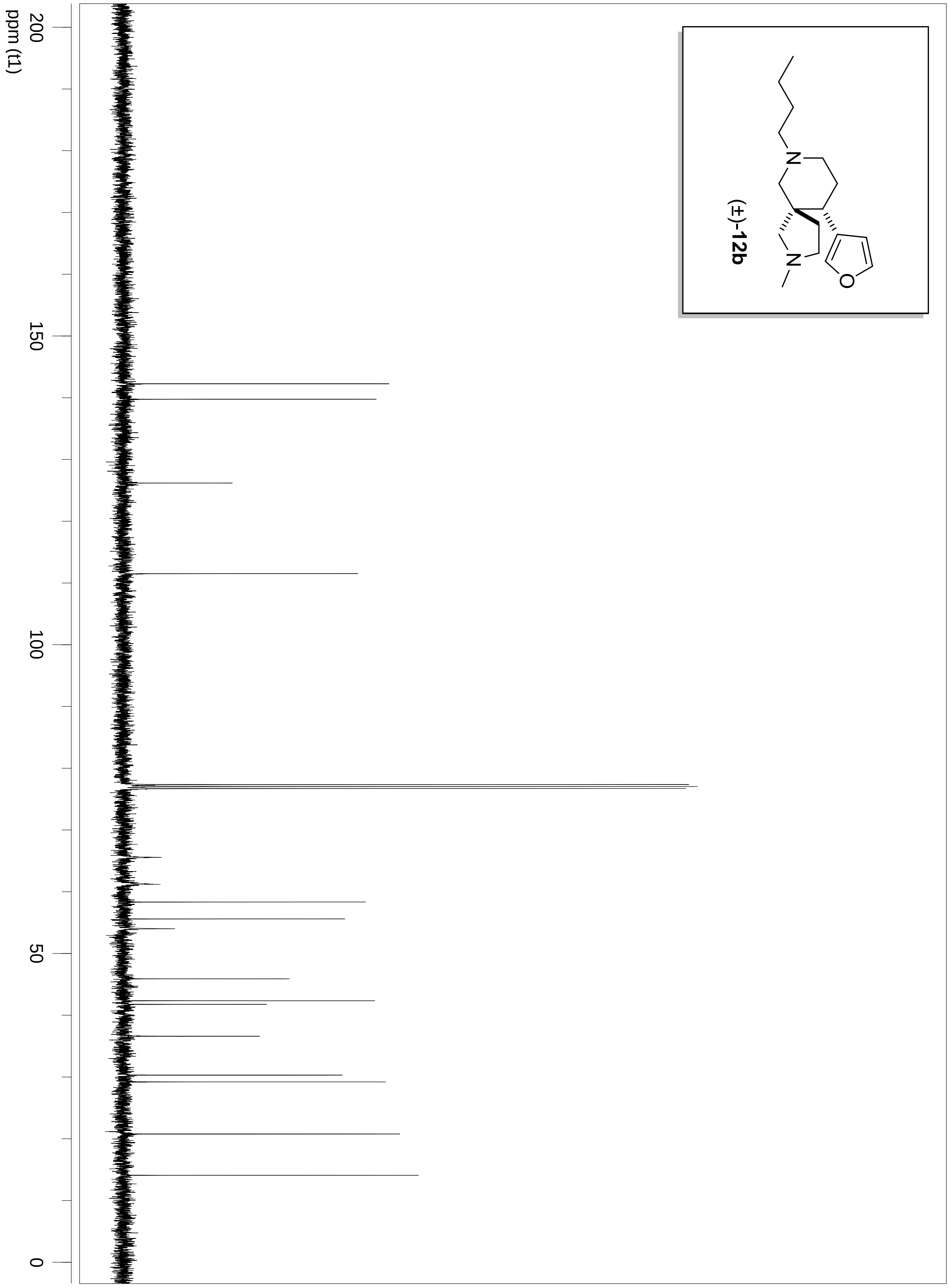

6. HPLC trace

6.1. HPLC trace of (±)-1a

Data File C:\HPCHEM\1\DATA\PAVOL\PAV00054.D   Sample Name: PJ 317 fr.11-21

PJ 317 fr. 11-21 cryst., 50 hex230, 0.6 ml/ml, press 49 barr.
injection 20 microliters, column OD-H

===

Injection Date : 06/10/2007 16:03:06 PM   Seq. Line : 1
Sample Name : PJ 317 fr.11-21   Location : Vial 16
Acq. Operator : Pavol   Inj : 1
Acq. Instrument : Instrument 1   Inj Volume : 20 µl
Acq. Method : C:\HPCHEM\1\METHODS\DJD26\50HEX230.M
Last changed : 06/10/2007 12:32:10 PM by Pavol
Analysis Method : C:\HPCHEM\1\METHODS\DJD26\90HEX220.M
Last changed : 05/10/2007 18:52:45 PM by Filippo
(modified after loading)

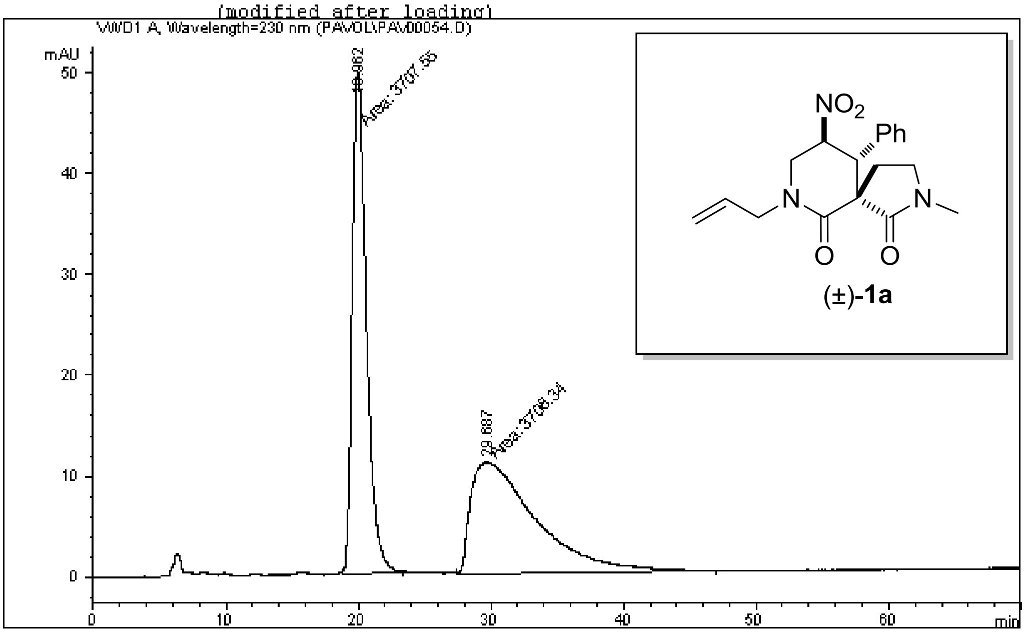

===
Area Percent Report
===

Sorted By : Signal
Multiplier : 1.0000
Dilution : 1.0000
Use Multiplier & Dilution Factor with ISTDs

Signal 1: VWD1 A, Wavelength=230 nm

Peak #	RetTime [min]	Type	Width [min]	Area [mAU*s]	Height [mAU]	Area %
1	19.962	MM	1.2393	3707.54761	49.86132	49.9947
2	29.687	MM	5.6334	3708.33813	10.97138	50.0053
Totals :				7415.88574	60.83270	

Results obtained with enhanced integrator!

===

*** End of Report ***

Instrument 1 07/10/2007 13:29:05 PM Filippo   Page 1 of 1

6.2. HPLC trace of (+)-1a.

Data File C:\HPCHEM\1\DATA\PAVOL\PAV00058.D Sample Name: PJ 407 fr.23-40

PJ 407 fr. 23-40 cryst., 50 hex230, 0.6 ml/ml, press 49 barr.
injection 20 microliters, column OD-H

===

Injection Date : 06/10/2007 20:46:07 PM Seq. Line : 5
Sample Name : PJ 407 fr.23-40 Location : Vial 20
Acq. Operator : Pavol Inj : 1
Acq. Instrument : Instrument 1 Inj Volume : 20 µl
Acq. Method : C:\HPCHEM\1\METHODS\DJD26\50HEX230.M
Last changed : 06/10/2007 12:32:10 PM by Pavol
Analysis Method : C:\HPCHEM\1\METHODS\DJD26\90HEX220.M
Last changed : 07/10/2007 13:29:08 PM by Filippo
(modified after loading)

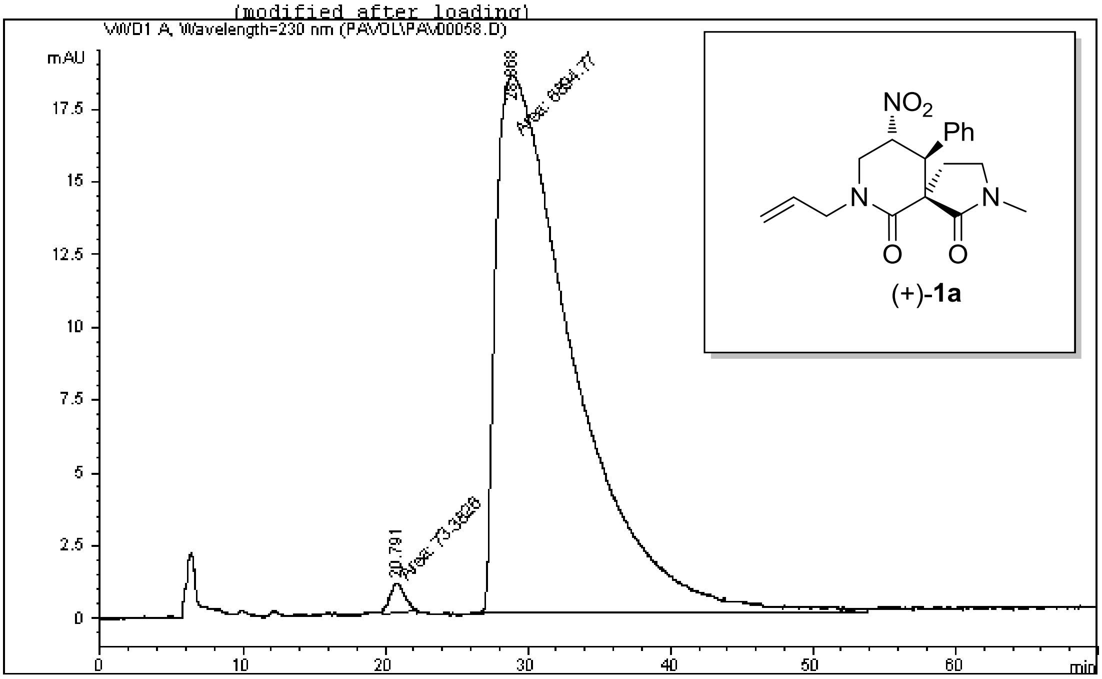

===

Area Percent Report

===

Sorted By : Signal
Multiplier : 1.0000
Dilution : 1.0000
Use Multiplier & Dilution Factor with ISTDs

Signal 1: VWD1 A, Wavelength=230 nm

Peak #	RetTime [min]	Type	Width [min]	Area [mAU*s]	Height [mAU]	Area %
1	20.791	MM	1.2085	73.38259	1.01207	1.0531
2	28.868	MM	6.2410	6894.76709	18.41252	98.9469
Totals :				6968.14968	19.42459	

Results obtained with enhanced integrator!

===

*** End of Report ***

Instrument 1 07/10/2007 13:30:37 PM Filippo Page 1 of 1
